# Novel carbazolyl–thiazolyl–chromone and carbazolyl–thiazolyl–pyrazole hybrids: synthesis, cytotoxicity evaluation and molecular docking studies†

**DOI:** 10.1039/d4ra03188a

**Published:** 2024-05-28

**Authors:** Noha M. Hassanin, Tarik E. Ali, Mohammed A. Assiri, Somaia M. Abdel-Kariem

**Affiliations:** a Department of Chemistry, Faculty of Education, Ain Shams University Cairo Egypt noha-mohamed@edu.asu.edu.eg; b Central Labs, King Khalid University, AlQuraa Abha Saudi Arabia; c Department of Chemistry, Faculty of Science, King Khalid University, AlQuraa Abha Saudi Arabia

## Abstract

A simple synthetic method was performed to design a novel series of polycyclic systems consisting of carbazole–thiazolidinone–chromone hybrids 4a–e and carbazole–thiazolidinone–pyrazole hybrids 5a–e in excellent yields. The methodology depended on the one-pot four-component reaction of 3-amino-9-ethylcarbazole, substituted isothiocyanates, ethyl bromoacetate and 6-methyl-3-formylchromone in ethanol under ultrasound waves at 50 °C to give the carbazole–thiazolidinone–chromone hybrids 4a–e. The latter isolated products were treated with hydrazine hydrate in ethanol under ultrasound waves at 50 °C affording the corresponding carbazole–thiazolidinone–pyrazole hybrids 5a–e. Spectral and analytical data confirmed the structures of all the synthesized compounds. The target compounds were screened for their *in vitro* anticancer activities against HCT116, PC3 and HepG2 cancer cell lines using the standard SRB method. Fortunately, both compounds 5dand5e were the most active against all cancer cell lines compared with doxorubicin and can be promising anticancer agents. Both bioactive products 5band5e were studied by the molecular docking to see how they bind with VEGFR-2 receptor. The results indicated that those compounds exhibited high affinities towards VEGFR-2 and established remarkably similar interactions to those of the powerful VEGFR-2-KDR.

## Abstract 2

A simple synthetic method was performed to design a novel series of polycyclic systems consisting of carbazole–thiazolidinone–chromone hybrids 4a–e and carbazole–thiazolidinone–pyrazole hybrids 5a–e in excellent yields.

## Graphical abstract

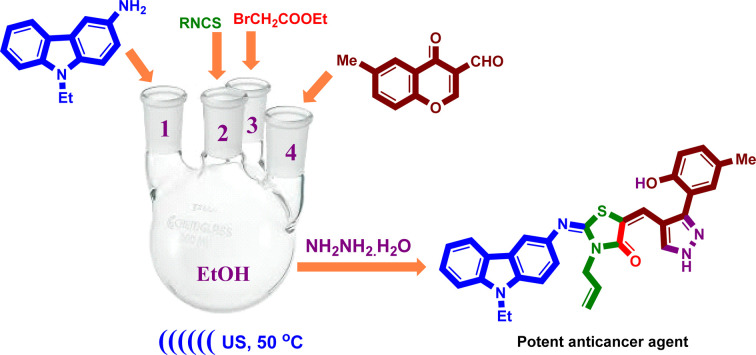

## Introduction

Cancer disease is the leading cause of morbidity and mortality in the world. Cancer is influenced by a number of factors such as genes, sex, age, food habits, stress, radiation and chemical agents.^1,2^ Recent advances in our understanding of cancer's molecular pathways have fueled a surge in cancer drug design research. This has led to the evaluation of diverse small molecule templates as potential cancer therapies.^3,4^ With these advances and findings, a number of pharmaceuticals are now often used to treat cancer. Although these medications have demonstrably extended patient lifespans, treatment success is not at the expected level. In recent years, researchers worked on specifically tumor-targeted substances to achieve the required levels. In the development of cancer drugs, design of a hybrid molecule has gained importance.^5^ The molecular hybridization methodology depends on the merging of pharmacophoric groups of multiple bioactive compounds in one molecular frame to create a unique hybrid product that is more active and less toxic than currently available pharmaceuticals. Hybrid molecules possess multiple implications in chemistry, pharmacology, and medicine. As cancer disease may have pathway cross-talks at certain phases, effective anticancer agents can be obtained through the synthesis of new hybrid compounds by integrating several hybrids.^6–10^

**Fig. 1:**
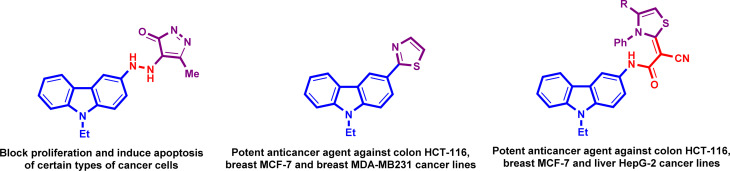
The chemical structures of some carbazole-based thiazole and pyrazole at the C-3 position.

Carbazoles are one of the most widespread substances in structures of bioactive compounds. They have several different pharmacological features, including anti-inflammatory, antiviral, antimicrobial, anticancer, analgesic, neuroprotective and inhibiting properties of pancreatic lipase.^11–17^ Drug researchers have demonstrated an abundance of interest in carbazole-based hybrid compounds because they have a considerable inhibitory effect on a number of cancer cell lines when possessing heterocyclic systems especially thiazole and pyrazole at the C-3 position (Fig. 1). The anticancer properties of carbazole moiety are well recognized, and its associations with other moieties can improve its bioactive properties, while one core may exhibit anticancer effects, and the other cores may inhibit the cross-talk the way.^18^

**Fig. 2:**
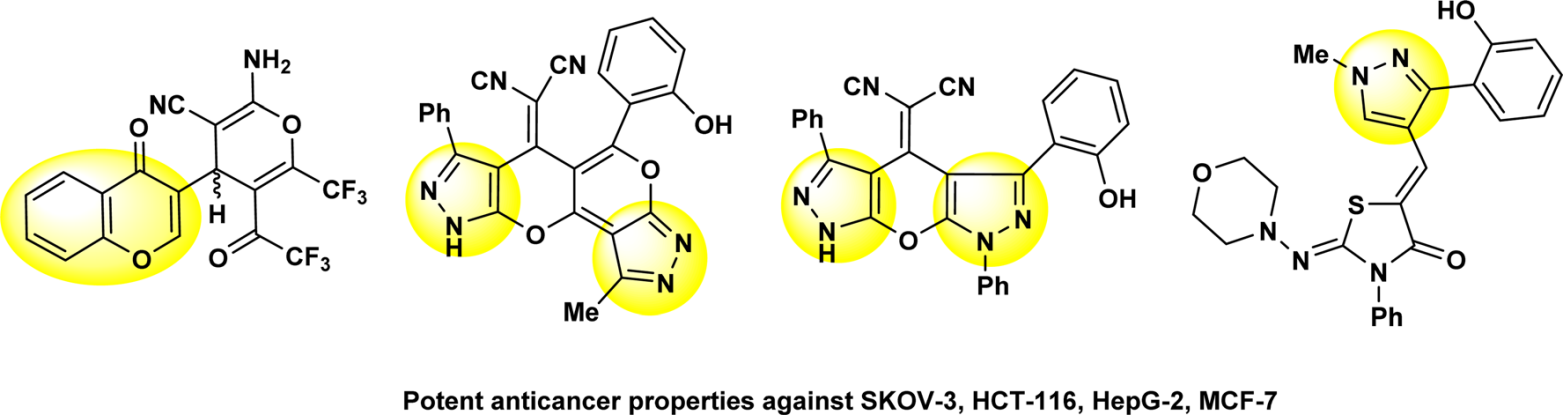
Some chemical structures of our published compounds that have chromone and pyrazole compounds with their potent anticancer activities.

On the other hand, thiazole derivatives have also gained increased attention due to their wide range of pharmacological uses and biological properties, including their analgesic, anti-HIV, antihypertensive, anti-inflammatory, antibacterial and herbicidal activity.^19–23^ Our previously investigations showed that chromone and pyrazole compounds have good anticancer activities when they were screened against some types of human cancer cells of breast (MCF-7), liver (HepG2), colon (HCT116) and ovary (SKOV-3) cancer (Fig. 2).^24–27^

In contrast to traditional methods, multicomponent reactions (MCRs) offer a powerful and efficient approach in organic synthesis. MCRs streamline the creation of medicinal drugs thanks to their simplicity, minimal waste, high productivity, excellent yields, and straightforward workup processes.^28–31^ Furthermore, ultrasound-assisted organic synthesis is an effective and has a number of advantages over traditional synthesis techniques, including the ability to decrease both time and electricity, and a shortened work-up procedure.^32–37^ In addition, ultrasound irradiation as a simple instrument and clean synthetic procedure can give a good yield of product.^38–43^

In the present study, we synthesized novel molecular frames by combining carbazole and thiazolidinone cores with either chromone or pyrazole core. The synthesis of the target compounds was designed in one-pot four-component reaction with the help of ultrasound waves. These compounds were then evaluated for their ability to tumor HCT116, PC3 and HepG2 cancer cell lines.

## Results and discussion

### Synthesis and characterization

**Scheme 1:**
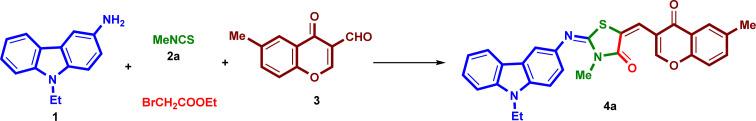
The model reaction of 3-amino-9-ethylcarbazole (1), methyl isothiocyanates 2a, ethyl bromoacetate and 6-methyl-3-formylchromone (3).

New molecular hybrids, including three heterocycles which are carbazole, thiazolidinone, and chromone or pyrazole, were designed and evaluated for their anticancer activities. These products were synthesized in one-pot four-component domino reaction with help of ultrasound irradiation. We checked different conditions to find a suitable and effective procedure for obtaining of 2-{[(9-ethyl-9*H*-carbazol-3-yl)imino]-5-[(6-methyl-4-oxo-4*H*-chromen-3-yl)methylene]}-3-substituted-thiazolidin-4-ones (4a–e). For optimization study, the reaction of 3-amino-9-ethylcarbazole (1), methyl isothiocyanates 2a, ethyl bromoacetate and 6-methyl-3-formyl-chromone (3) was selected as a model reaction (Scheme 1 and Table 1).

**Table 1:** Optimization of various catalysts and reaction conditions for the model reaction

Entry	Catalyst	Solvent	Time (min)	Temp. (^o^C)	Yield^a^ (%)
1	—	—	180	rt	—
2	—	—	180	75	—
3	—	Benzene	180	50	12%
4	—	MeOH	180	50	18%
5	—	EtOH	180	50	22%
6	—	EtOH	180	75	41%
7	DBU (1.0 mmol)	EtOH	180	75	42%
8	Pyridine (1.0 mmol)	EtOH	180	75	44%
9	Et_3_N (1.0 mmol)	EtOH	180	75	52%
10	Et_3_N (2.0 mmol)	EtOH	180	75	75%
11	Et_3_N (2.0 mmol), US	EtOH	25	25	80%
12	Et_3_N (2.0 mmol), US	EtOH	50	50	91%
13	Et_3_N (2.0 mmol), US	EtOH	75	75	91%

a Reagents: 3-amino-9-ethylcarbazole (1.0 mmol), methyl isothiocyanate (1.0 mmol), ethyl bromoacetate (1.0 mmol), and 6-methyl-3-formylchromone (1.0 mmol).

When the four-components reacted under catalyst- and solvent-free conditions for 3 h at room temperature or 75 °C, no product was isolated (Table 1, entries 1 and 2). When the reaction was performed without a catalyst in the presence of benzene or methanol or absolute ethanol at 50 °C, it gave the desired product 4a in 12%, 18% and 22% yields, respectively (Table 1, entries 3–5), while in the presence of absolute ethanol at 75 °C for 3 h, the product 4a was obtained in 41% yield (Table 1, entry 6). Thus, we decided to use some bases such as DBU, pyridine and Et_3_N (1.0 mmol) (Table 1, entries 7–9). It was found that Et_3_N as a catalyst led to the considerable product 4a in 52% yield after 3 h (Table 1, entry 9). Therefore, the effect of the quantity of Et_3_N was examined. Increasing of the amount of the catalyst into 2.0 mmol increased the yield of the product to 75% (Table 1, entry 10). Next, we tried to do this reaction in a shorter time with better yield by using ultrasonication effect. When the target reaction was carried out by four-components reaction in the presence of Et_3_N (2.0 mmol) under ultrasound irradiation at room temperature, it gave the product 4a in 80% yield after 25 minutes (Table 1, entry 11). But, when the reaction was repeated under the same reaction condition at 50 °C, it gave the final product 4a in 91% as excellent yield after 50 min (Table 1, entry 12). The yield did not progress above 91% although increasing the time into 75 minutes and temperature into 75 °C (Table 1, entry 13).

**Scheme 2:**
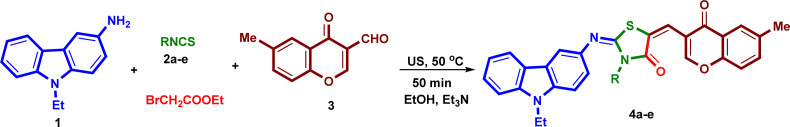
Reaction of 3-amino-9-ethylcarbazole (1), alkyl/aryl isothiocyanates 2a–e, ethyl bromoacetate and 6-methyl-3-formylchromone (3) in one-pot four-component domino reaction.

**Scheme 3:**
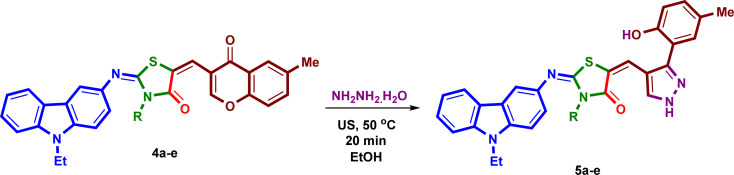
Reaction of the products 4a–e with hydrazine hydrate.

**Fig. 3:**
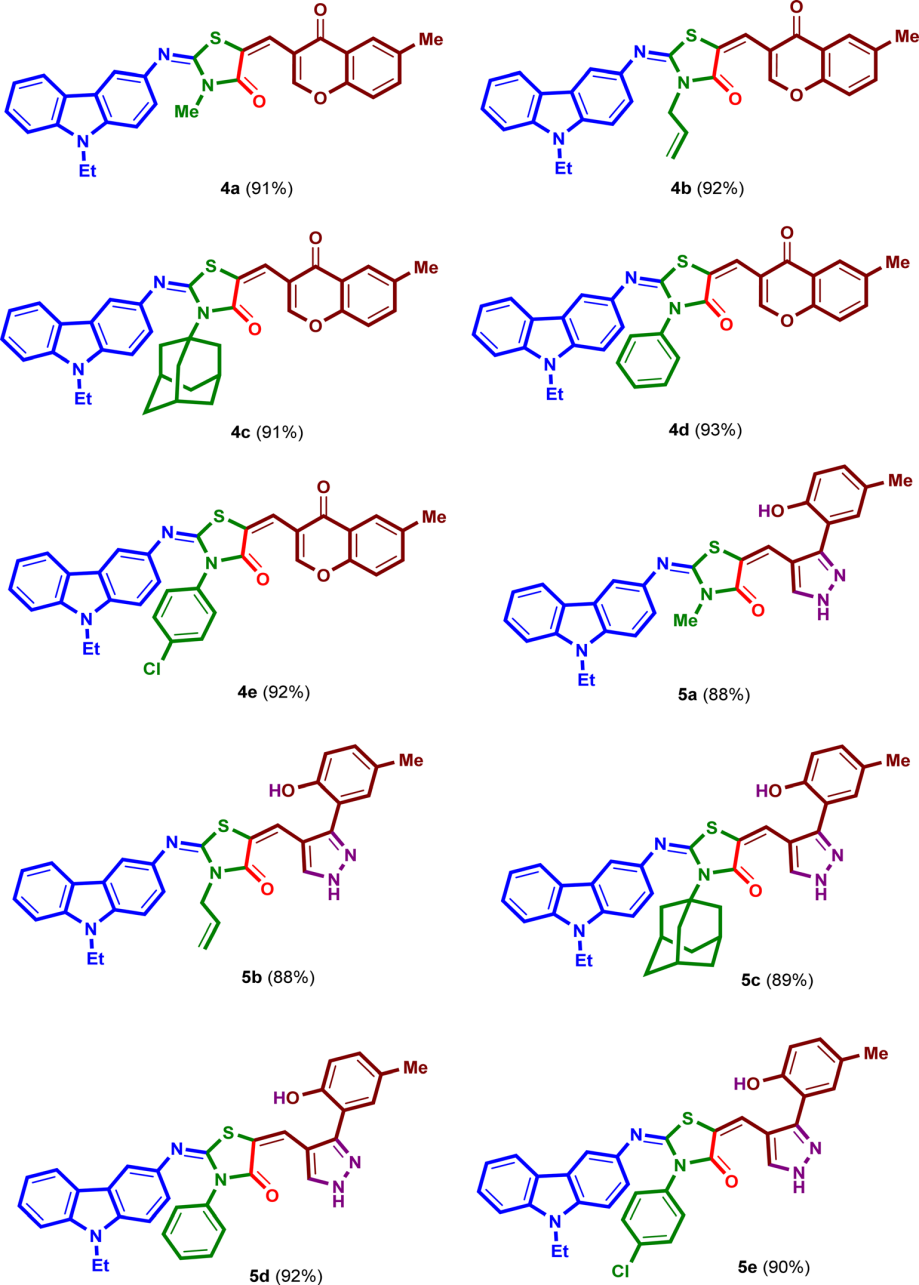
The chemical structures of the synthetic compounds 4a–e and 5a–e and their yields.

Under the optimized reaction conditions, a mixture of 3-amino-9-ethylcarbazole (1), alkyl/aryl isothiocyanates 2a–e, ethyl bromoacetate and 6-methyl-3-formyl-chromone (3), was irradiated under ultrasound at 50 °C for 50 minutes in absolute ethanol in the presence of triethylamine in three steps to yield the corresponding 2-{[(9-ethyl-9*H*-carbazol-3-yl)imino]-5-[(6-methyl-4-oxo-4*H*-chromen-3-yl)methylene]}-3-substituted-thiazolidin-4-ones (4a–e) in excellent yields 91–93% (Scheme 2). The isolated products 4a–e were reacted with hydrazine hydrate under ultrasound at 50 °C for 20 minutes in absolute ethanol to afford the corresponding 2-{[(9-ethyl-9*H*-carbazol-3-yl)imino]-5-[(3-(2-hydroxy-5-methylphenyl)-1*H*-pyrazol-4-yl)] methylene}-3-substituted-thiazolidin-4-ones (5a–e) in high yields 88–92% (Scheme 3). The structures and reaction yields of new compounds 4a–e and 5a–e are illustrated in Fig. 3.

The spectral data as described in the experimental section confirmed the structures of all isolated products. For instance, the IR spectrum of a carbazole–thiazolidine–chromone system 4b exhibited characteristic peaks at 1698 (C=O_thiazolidinone_) and 1654 (C=O_chromone_) cm^−1^. Additionally, weak absorption bands at 3052, 2974, and 2938 cm^−1^ were assigned to the stretching vibrations of aromatic and aliphatic groups in 4b. The C=N and C=C stretching bands were observed in the range of 1634–1560 cm^−1^. The ^1^H-NMR spectrum of compound 4b showed specific singlets at *δ* 8.72 and 7.55 ppm due to H-2_chromone_ and =CH_exocyclic_ protons, respectively. Also, its ^1^H-NMR spectrum displayed multiplets belonging to 10 aromatic protons of carbazole and chromone rings in the range of *δ* 7.09–8.14 ppm, while the allylic protons were resonated at *δ* 4.55 (d, *J* = 4.8 Hz, CH_2_), 5.27 (dd, *J* = 6.4 and 1.2 Hz, =CH_2_), and 5.97–6.06 (m, =CH).^44,45^ The signals of the methyl and ethyl groups appeared at *δ* 2.33 (NCH_3_) and 1.35, 4.44 (NCH_2_CH_3_) ppm. In the ^13^C-NMR spectrum of this compound, the signals of C=O_thiazolidinone_, C=O_chromone_, and C=N carbons were observed at *δ* 166.2, 175.1 and 160.6 ppm, respectively.^46,47^ The C-2_chromone_ and =CH_exocyclic_ carbons also resonated at *δ* 154.1 and 126.5 ppm, respectively.^48^ The allylic carbons were resonated at *δ* 45.0 (CH_2_), 117.5 (=CH_2_) and 140.4 (=CH). Besides, the signals of NCH_3_ and CH_3_CH_2_N groups were observed at *δ* 20.8 and 14.3, 37.6 ppm, respectively. The molecular ion peak of 4b was recorded at *m*/*z* 519 (M^+^, 27%) in its mass spectrum. In the same manner, the IR spectrum of the carbazole–thiazolidine–pyrazole system 5b showed the OH, NH and C=O_thiazolidinone_ functions at 3256, 3166 and 1695 cm^−1^, respectively. The absence of C=O_chromone_ and appearance of OH and NH groups confirmed the ring opening and ring closure. The ^1^H-NMR spectrum of 5b displayed four specific singlets at *δ* 13.36, 9.77, 7.83 (d, *J* = 2.4 Hz) and 7.45 ppm due to OH, NH, H-5_pyrazole_ and =CH_exocyclic_ protons, respectively.^49^ Further, in the ^1^H-NMR spectrum of this compound, the allylic protons was observed at *δ* 4.53 (d, *J* = 5.2 Hz, CH_2_), 5.25 (dd, *J* = 6.4 and 1.2 Hz, =CH_2_) and 5.95–6.04 (m, =CH). The signals of the methyl and ethyl groups appeared at *δ* 2.24 (NCH_3_) and 1.36, 4.46 (NCH_2_CH_3_) ppm. Furthermore, the ^13^C-NMR spectrum of 5b exhibited signals of C=O_thiazolidinone_, C-3_pyrazole_ and C-5_pyrazole_, carbon atoms at *δ* 166.4, 149.8 and 140.6 ppm, respectively.^50^ The C-2_thiazolidinone_ and =CH_exocyclic_ carbons resonated at *δ* 162.7 and 126.5 ppm, respectively. Besides, the allylic carbons were exhibited at *δ* 45.0 (CH_2_), 116.4 (=CH_2_) and 140.3 (=CH). Finally, its molecular ion peak M^+^ was displayed at *m*/*z* 533 (31%) in its mass spectrum.

**Scheme 4:**
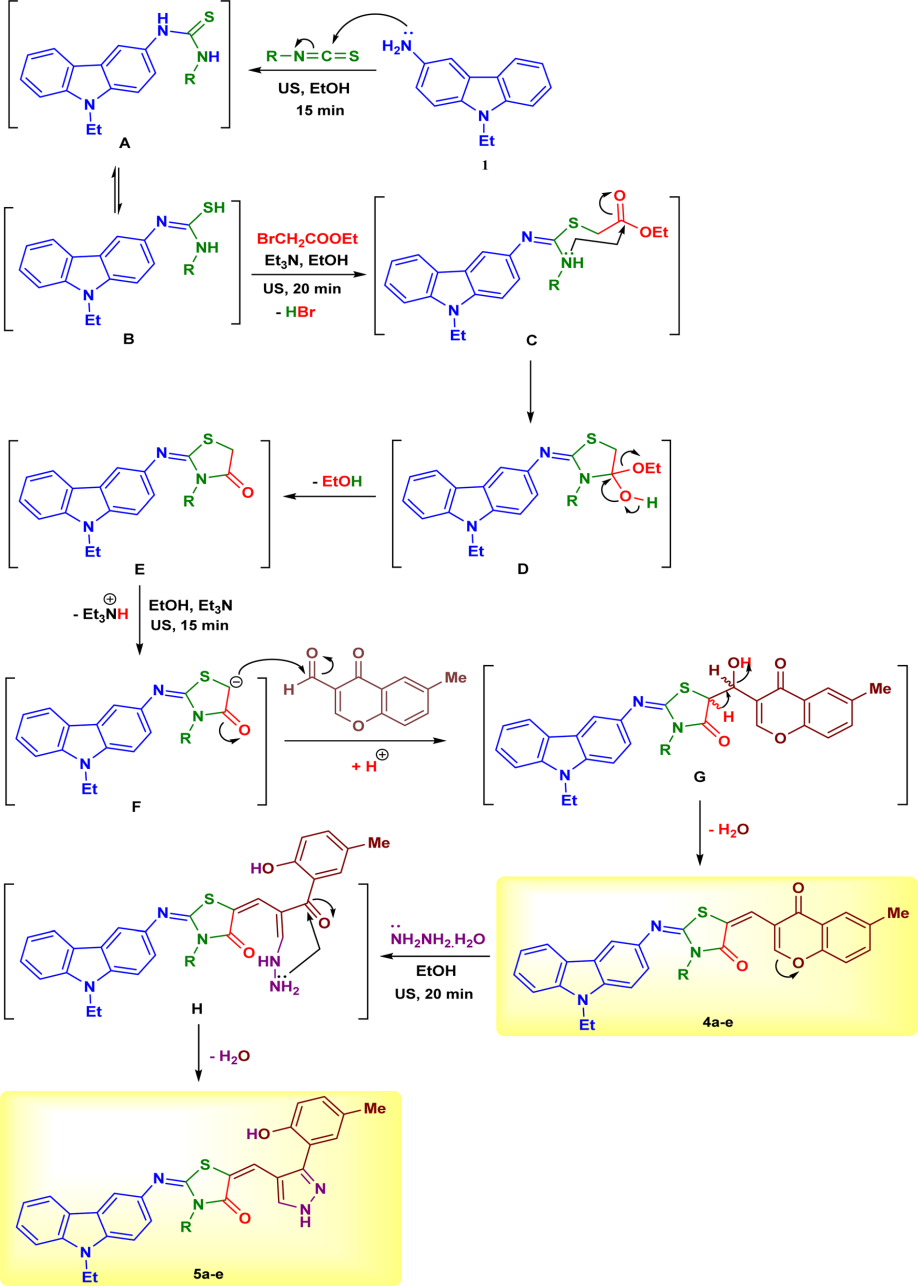
The proposed reaction mechanism for formation of the products 4a–e and 5a–e.

The steps and possible mechanisms for the formation of the target products were depicted in Scheme 4. 3-Amino-9-ethylcarbazole (1) underwent a nucleophilic addition to a series of isothiocyanates 2a–e in ethanol at room temperature for 15 min under ultrasound irradiation, to form the corresponding carbazolyl–thioureas A which can exist in the tautomeric form B. The thiol group SH attacked the CH_2_ group in ethyl bromoacetate to release HBr molecule to form the intermediate C. The later intermediate underwent cyclization through nucleophilic attack of NH group at ester group (intermediate D), followed by removal of ethanol molecule to give the carbazolyl–thiazolidinones E. In the next step, the intermediate E was deprotonated with the help of triethylamine to attack the formyl group of compound 3 (intermediate F), followed by removal of water molecule to isolate the carbazole–thiazolidinone–chromones 4a–e (Scheme 4). The C-2 positions of chromone ring are more electrophilic centers and can be attacked by hydrazine hydrate to undergo ring opening (intermediate H). The ring closure of the latter intermediate H by nucleophilic attack of NH_2_ group at the ketonic C=O and releasing of water molecule afforded the final carbazole–thiazolidinone–pyrazoles 5a–e (Scheme 4).

### Anticancer properties

#### Evaluation of cytotoxicity properties

We evaluated the effect of new compounds (4a–e and 5a–e) and the standard drug (doxorubicin) on the growth of various tumor cancer cells. We used the sulforhodamine B (SRB) assay to measure this effect on three different cancer cell lines (HCT116, PC3, and HepG2) and a normal healthy cell line from a mouse (3T3-L1).^51,52^ The IC_50_ values for all synthesized compounds and doxorubicin are shown in Table 2. As per National Cancer Institute guidelines, a IC_50_ value of ≤10 μg ml^−1^ was considered to demonstrate highly significant inhibitory activity. Among the tested compounds on HCT-116 cancer cells, compound 5b emerged as the most potent inhibitor of cell growth with the lowest IC_50_ value at 28.5 ± 1.5 μg ml^−1^. Conversely, compound 5a displayed the weakest effect by its highest IC_50_ value at 69.5 ± 1.5 μg ml^−1^. The other compounds, 5c and 5e exhibited moderate antiproliferative activity with IC_50_ values at 30.9 ± 2.3 and 39.9 ± 0.1 μg ml^−1^, respectively. As for PC3 cells, both compounds 5b and 5e showed the highest antiproliferative activities with the smallest IC_50_ values at 4.9 ± 0.8 and 9.2 ± 0.4 μg ml^−1^, respectively, while compounds 5a and 5c displayed acceptable activities with IC_50_ value at 12.9 ± 3.8 and 37.4 ± 1.6 μg ml^−1^, respectively. Furthermore, compound 5e seems to be quite versatile. It demonstrated the most potent activity against HepG-2 cells with an impressive IC_50_ value at 9.1 ± 0.5 μg ml^−1^. In addition, compounds 5a–c showed good activities against HepG-2 cells with IC_50_ values between 18.5 ± 0.4 and 25.3 ± 1.4 μg ml^−1^. The selectivity of these promising synthesized compounds (5a, 5b, 5c and 5e) in thier cytotoxicity between cancer and healthy cells is very important to be a chemotherapeutic agent. Here, their antiproliferative effects on the normal mouse fibroblast cell line, 3T3-L1 were evaluated. The order of toxicity power of these compounds on 3T3-L1 cells was 5c > 5a > 5e > 5b. This suggested that both compound 5b and 5e may be effective against a broader range of cancer cell lines.

**Table 2:** The *in vitro* anticancer activity of products 4a–e and 5a–e against HCT-116, PC3, HepG-2 and 3T3-L1 cancer line cells^b^

Compd.	IC_50_^a^ (μg mL^−1^)			
	HCT-116	PC3	HePG-2	3T3-L1
4a	>100	>100	>100	ND
4b	>100	>100	>100	ND
4c	>100	>100	>100	ND
4d	>100	>100	>100	ND
4e	>100	>100	>100	ND
5a	69.5 ± 1.5	12.9 ± 3.8	20.1 ± 0.2	153.13 ± 2.30
5b	28.5 ± 1.5	4.9 ± 0.8	18.5 ± 0.4	178.13 ± 2.42
5c	30.9 ± 2.3	37.4 ± 1.6	25.3 ± 1.4	115.13 ± 1.85
5d	>100	>100	>100	ND
5e	39.9 ± 0.1	9.2 ± 0.4	9.1 ± 0.5	162.34 ± 2.94
Doxorubicin	6.9 ± 0.5	6.2 ± 0.9	7.9 ± 1.3	ND

a IC_50_ values are the mean ± SD of three separate experiments.

b ND: not determined.

#### Structure–activity relationship (SAR)

The study also explored the relationship between the chemical structures of the compounds and their anticancer activity against the tested cell lines (HCT116, PC3, and HepG2). They found that the presence of a pyrazole ring compared to a chromone ring connected with the carbazole–thiazolidinone scaffold led to a more effective pharmacophore. In simpler terms, the specific arrangement of atoms including a pyrazole ring seemed to be more critical for the cytotoxic effects than the arrangement with a chromone ring. This may be due to the presence of the phenolic OH group and the pyrazolic NH group which played vital roles in interaction with cancer cells. The anticancer properties of compounds 5a–c and e differed according to the types of cancer lines. Interestingly, considering the contribution of the group attached to the thiazolidinone ring at position 3 affected on the anticancer activity against all cancer cell lines. For HCT116 and PC3 cancer cells, allyl group was found to be the most effective than the other groups. In contrast to PC3 and HepG2 cancer cells, the 4-chlorophenyl group caused significantly increased in the anticancer activity in comparison with the other groups. Further, the allyl and 4-chlorophenyl groups caused low cytotoxicity on the normal cell 3T3-L1 cells. This indicated great importance to understand both the type of cell and the structure of the compound that could be targeted by anticancer agent.

#### Molecular docking study

**Fig. 4:**
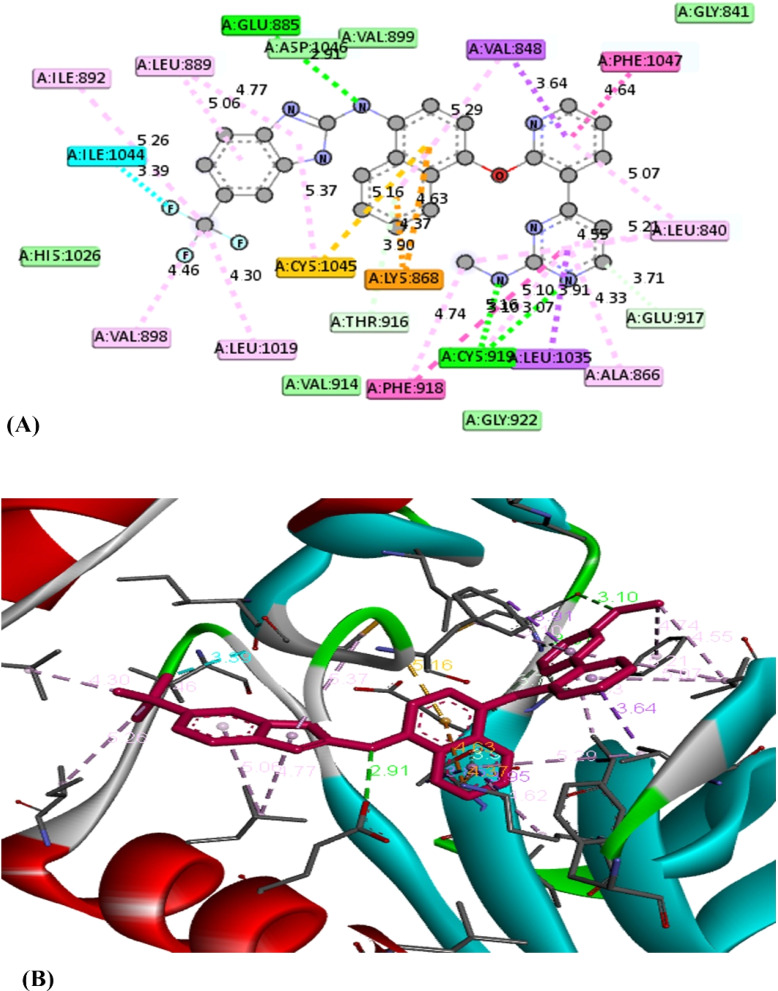
The 2D (A) and 3D (B) interactions of the K11 with VEGFR-2-KDR.

**Fig. 5:**
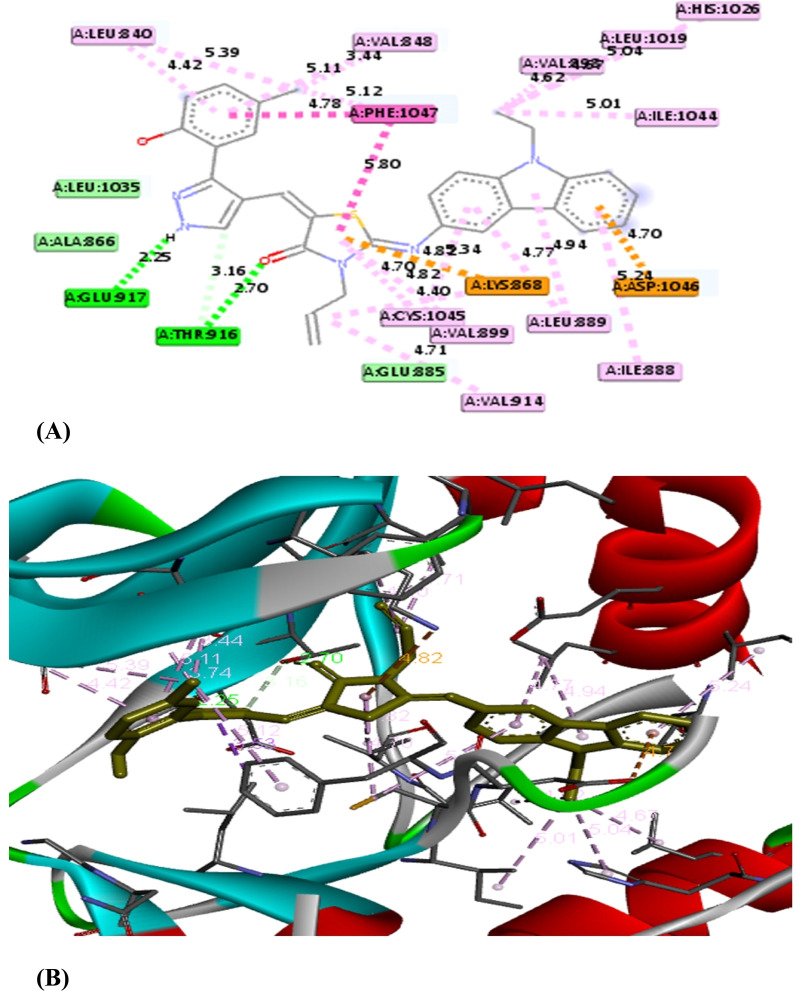
The 2D (A) and 3D (B) interactions of the compound 5b with VEGFR-2-KDR.

**Fig. 6:**
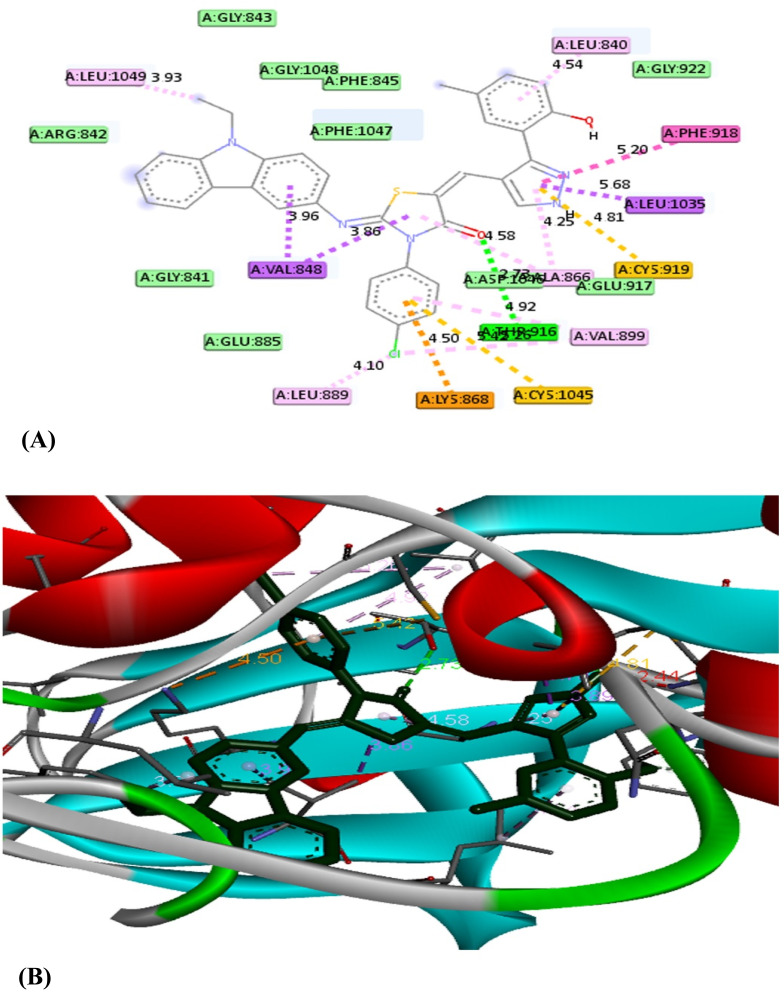
The 2D (A) and 3D (B) interactions of the compound 5e with VEGFR-2-KDR.

A molecular docking approach has become a crucial strategy that provides the most promising route for drug designing and discovery.^53^ It is used to predict the strength of binding affinity between a drug target and ligand molecule. Additionally, this computational tool is more economic, time saving and effective over conventional technologies. According to the cytotoxicity results, this study was applied for the most effective compounds 5b and 5e to explore their binding mode towards vascular endothelial growth factor Kinase Insert Domain Receptor. The molecular docking results suggested that the synthesized compounds bind to VEGFR-2 in a similar way to a known VEGFR-2 inhibitor called K11 as shown in Table 3.^54–59^Fig. 4 illustrates how K11 interacted with VEGFR-2. It formed two hydrogen bonds with key amino acids CYS 919 and GLU 885, and its fluorine atoms interacted with another key amino acid ILE 1044. In addition, it formed several Pi-interactions including Pi–sulfur (CYS 1045), two Pi–sigma (VAL 848, LEU 1035), Pi–cation (LYS 868), and Pi–Pi T shaped (PHE 1047). According to the molecular docking results, both compounds 5b and 5e interacted with the VEGFR-2 protein in very similar binding modes, while their binding are −11.2 and −10.1 kcal mol^−1^, respectively. Both 5b and 5e fitted into the same pocket on the VEGFR-2 protein. Compound 5b revealed several modes of interactions including hydrogen bonds with GLU 917, THR 916 through NH group of pyrazole ring and the C=O group of the thiazole ring, respectively. Moreover, some Pi-interactions were observed including Pi–cation with LYS 868 through the thiazole ring, Pi–anion with ASP 1046 through the phenyl ring of carbazole moiety and Pi–alkyl with carbazole or thiazole moieties through the amino acids VAL 848 and LEU 889. Also, Pi–Pi T shaped interaction was observed with residual key PHE 1047 through the thiazole ring. With respect to product 5e, it displayed seven modes of interactions involving hydrogen bond with the amino acid THR 916 through C=O group of thiazole ring. Besides, it formed Pi–sigma interactions through carbazole, thiazole and pyrazole moieties with amino acids LEU 1035 and VAL 848. In addition, it formed Pi–cation, Pi–sulfur and Pi–alkyl interactions with key residue LYS 868, CYS 919, CYS 1045 and LEU 889, respectively through 4-chlorophenyl ring. From the above mentioned, the bioactive synthesized compounds 5b and 5e showed high affinities towards VEGFR-2 which correlated well with the results obtained from experimental cytotoxicity. Therefore, these compounds can be considered promising anticancer agents Fig. 5 and 6

**Table 3:** The interactions of compounds 5b, 5e and K11 with VEGFR-2-KDR (3EWH)

Compd.	Affinity (kcal mol^−1^)	Amino acid	Interaction types	Distance (Å)
5b	−11.2	GLU 917	Hydrogen bond	2.25
		THR 916	Hydrogen bond	2.70
		LYS 868	Pi–cation	4.82
		ASP 1046	Pi–anion	4.70
		VAL 848	Pi–alkyl	3.44
		LEU 889	Pi–alkyl	4.77
		PHE 1047	Pi–Pi T shaped	4.52
5e	−10.1	THR 916	Hydrogen bond	2.73
		LYS 868	Pi–cation	4.50
		CYS 919	Pi–sulfur	4.81
		LEU 1035	Pi–sigma	5.68
		VAL 848	Pi–sigma	3.86
		LEU 889	Pi–alkyl	4.10
		CYS 1045	Pi–sulfur	5.42
K11	−12.6	CYS 919	Hydrogen bond	3.07
		GLU 885	Hydrogen bond	2.91
		CYS 1045	Pi–sulfur	5.16
		VAL 848	Pi–sigma	3.64
		LEU 1035	Pi–sigma	3.91
		PHE 1047	Pi–Pi T shaped	4.64
		LYS 868	Pi–cation	3.90
		ILE 1044	Halogen (fluorine)	3.39

## Experimental

### General marks

The melting points were determined in an open capillary tube on a digital Stuart SMP-3 apparatus. IR spectra were measured on FT-IR (Nicolet IS10) spectrophotometer using ATR technique. The ^1^H- and ^13^C-NMR spectra were measured a Bruker spectrometer (400 and 100 MHz), using DMSO-*d*_6_ as a solvent and TMS (*δ*) as an internal standard. Mass spectra were recorded on direct probe controller inlet part to single quadropole mass analyzer in (Thermo Scientific GCMS). Elemental microanalyses were performed PerkinElmer 2400II at the Chemical War department, Ministry of Defense. The purity of the synthesized compounds was checked by thin layer chromatography (TLC) and elemental microanalysis. All the used fine chemicals and solvents were purchased form Sigma-Aldrich company and used without further purification.

### Synthesis of 2-{[(9-ethyl-9*H*-carbazol-3-yl)imino]-5-[(6-methyl-4-oxo-4*H*-chromen-3-yl) methylene]}-3-substituted-1,3-thiazolidin-4-ones (4a–e)

A mixture of 3-amino-9-ethylcarbazole (1) (2.5 mmol, 0.525 g) and alkyl/aryl isothiocyanates 2a–e (2.5 mmol) was dissolved in absolute ethanol (20 ml) and irradiated under ultrasonication waves for 15 min at 50 °C. Then, adding a solution of ethyl bromoacetate (2.5 mmol, 0.276 ml) in absolute ethanol (5 ml) containing triethylamine (5 mmol, 0.7 ml) to the previous mixture and further irradiated under ultrasonication waves at 50 °C for 20 min. Next, a solution of 6-methyl-3-formylchromone (3) (2.5 mmol, 0.47 g) in absolute ethanol (15 ml), was added and heated at 50 °C for 15 min under ultrasonication waves. The mixture was left to cool into room temperature. The formed solid was filtrated off and washed with water. The pure products were obtained after crystallization from ethanol.

### 2-[(9-Ethyl-9*H*-carbazol-3-yl)imino]-3-methyl-5-[(6-methyl-4-oxo-4*H*-chromen-3-yl) methylene]-1,3-thiazolidin-4-one (4a)

Yellow solid in 91% yield, mp 230–231 °C, IR (KBr), (*v*_max_, cm^−1^): 3061 (C–H_arom_), 2926, 2852 (C–H_aliph_), 1710 (C=O_thiazolidinone_), 1631 (C=O_chromone_), 1595, 1512 (C=N, C=C). ^1^H-NMR (400 MHz, DMSO-*d*_6_): 1.34 (t, 3H, *J* = 6.8 Hz, CH_3_), 2.32 (s, 3H, CH_3_), 3.36 (s, 3H, CH_3_), 4.42 (q, 2H, *J* = 6.8 Hz, NCH_2_), 7.11 (dd, 1H, *J* = 8.4 and 1.6 Hz, H-8_chromone_), 7.16 (t, 1H, *J* = 7.6 Hz, H-7_chromone_), 7.43–7.52 (m, 3H, CH= and H–carbazole), 7.56–7.61 (m, 3H, H–carbazole), 7.76–7.78 (m, 2H, H-5_chromone_ and H–carbazole), 8.12 (d, 1H, *J* = 8.0 Hz, H–carbazole), 8.67 (s, 1H, H-2_chromone_). ^13^C-NMR (100 MHz, DMSO-*d*_6_): 13.6 (CH_3_), 20.3 (CH_3_), 29.3 (CH_3_), 37.1 (NCH_2_), 109.3 (C-4a_carbazole_), 111.2 (C-1_carbazole_), 118.6 (C-8_chromone_), 118.8 (C-8_carbazole_), 119.6 (C-3_chromone_), 120.5 (C-6_carbazole_), 122.3 (C-4_carbazole_), 122.7 (C-7_carbazole_), 123.1 (C-5_carbazole_), 123.4 (C-2_carbazole_), 123.5 (C-4a_chromone_), 125.8 (C-5_chromone_), 126.3 (C-4b_carbazole_), 126.6 (=CH), 127.7 (C-5_thiazolidinone_), 128.9 (C-8a_carbazole_), 129.6 (C-7_chromone_), 134.8 (C-6_chromone_), 139.6 (C-9a_carbazole_), 146.8 (C-3_carbazole_), 153.6 (C-8a_chromone_), 154.1 (C-2_chromone_), 162.1 (C-2_thiazolidinone_), 165.9 (C-4_thiazolidinone_), 174.0 (C-4_chromone_). MS (*m*/*z*, I%): 493 (M^+^, 16%). Anal. calcd for C_29_H_23_N_3_O_3_S (493.58): C, 70.57%; H, 4.70%; N, 8.51%; S, 6.50%. Found: C, 70.42%; H, 4.53%; N, 8.32%; S, 6.34%.

### 3-Allyl-2-[(9-ethyl-9*H*-carbazol-3-yl)imino]-5-[(6-methyl-4-oxo-4*H*-chromen-3-yl) methylene]-1,3-thiazolidin-4-one (4b)

Yellow solid in 92% yield, mp 197–199 °C, IR (KBr), (*v*_max_, cm^−1^): 3052 (C–H_arom_), 2974, 2938 (C–H_aliph_), 1698 (C=O_thiazolidinone_), 1654 (C=O_chromone_), 1634, 1620, 1560 (C=N, C=C). ^1^H-NMR (400 MHz, DMSO-*d*_6_): 1.35 (t, 3H, *J* = 7.2 Hz, CH_3_), 2.33 (s, 3H, CH_3_), 4.44 (q, 2H, *J* = 7.2 Hz, NCH_2_), 4.55 (d, 2H, *J* = 4.8 Hz, CH_2_), 5.27 (dd, 2H, *J* = 6.4 and 1.2 Hz, =CH_2_), 5.97–6.06 (m, 1H, CH=), 7.09 (dd, 1H, *J* = 8.4 and 2.0 Hz, H-8_chromone_), 7.17 (t, 1H, *J* = 7.2 Hz, H-7_chromone_), 7.46 (t, 1H, *J* = 7.6 Hz, H–carbazole), 7.50–7.55 (m, 2H, CH= and H–carbazole), 7.59–7.63 (m, 3H, H–carbazole), 7.77 (d, 1H, *J* = 1.6 Hz, H–carbazole), 7.80 (s, 1H, H-5_chromone_), 8.14 (d, 1H, *J* = 7.6 Hz, H–carbazole), 8.72 (s, 1H, H-2_chromone_). ^13^C-NMR (100 MHz, DMSO-*d*_6_): ^13^C-NMR (100 MHz, DMSO-*d*_6_): 14.3 (CH_3_), 20.8 (CH_3_), 37.6 (NCH_2_), 45.0 (CH_2_), 109.6 (C-4a_carbazole_), 110.2 (C-1_carbazole_), 117.5 (=CH_2_), 118.6 (C-8_chromone_), 118.7 (C-8_carbazole_), 119.2 (C-3_chromone_), 120.1 (C-6_carbazole_), 121.1 (C-4_carbazole_), 122.4 (C-7_carbazole_), 122.6 (C-5_carbazole_), 123.0 (C-2_carbazole_), 123.1 (C-4a_chromone_), 123.8 (C-5_chromone_), 125.2 (C-4b_carbazole_), 126.5 (=CH), 127.2 (C-5_thiazolidinone_), 128.7 (C-8a_carbazole_), 132.0 (C-7_chromone_), 136.5 (C-6_chromone_), 137.5 (C-9a_carbazole_), 140.4 (=CH), 148.1 (C-3_carbazole_), 150.9 (C-8a_chromone_), 154.1 (C-2_chromone_), 160.6 (C-2_thiazolidinone_), 166.2 (C-4_thiazolidinone_), 175.1 (C-4_chromone_). MS (*m*/*z*, I%): 519 (M^+^, 27%). Anal. calcd for C_31_H_25_N_3_O_3_S (519.62): C, 71.66%; H, 4.85%; N, 8.09%; S, 6.17%. Found: C, 71.52%; H, 4.69%; N, 7.97%; S, 5.99%.

### 3-(Adamantan-1-yl)-2-[(9-ethyl-9*H*-carbazol-3-yl)imino]-5-[(6-methyl-4-oxo-4*H*-chromen-3-yl)methylene]-1,3-thiazolidin-4-one (4c)

Beige solid in 91% yield, mp 242–244 °C, IR (KBr), (*v*_max_, cm^−1^): 3052 (C–H_arom_), 2978, 2904, 2855 (C–H_aliph_), 1697 (C=O_thiazolidinone_), 1645 (C=O_chromone_), 1621, 1598, 1561 (C=N, C=C). ^1^H-NMR (400 MHz, DMSO-*d*_6_): 1.34 (t, 3H, *J* = 6.8 Hz, CH_3_), 1.59–1.63 (m, 6H, CH_2_), 1.79–1.84 (m, 6H, CH_2_), 2.00–2.04 (m, 3H, CH_2_), 2.46 (s, 3H, CH_3_), 4.46 (q, 2H, *J* = 6.8 Hz, NCH_2_), 7.21 (t, 1H, *J* = 6.4 Hz, H-7_chromone_), 7.33 (dd, 1H, *J* = 8.4 and 1.6 Hz, H-8_chromone_), 7.46–7.51 (m, 1H, H–carbazole), 7.59 (s, 1H, CH=), 7.62–7.72 (m, 3H, H–carbazole), 7.96–7.98 (m, 2H, H–carbazole), 8.07 (s, 1H, H-5_chromone_), 8.14 (dd, 1H, *J* = 8.4 and 3.6 Hz, H–carbazole), 8.83 (s, 1H, H-2_chromone_). ^13^C-NMR (100 MHz, DMSO-*d*_6_): 14.2 (CH_3_), 20.7 (CH_3_), 29.4 (3CH_adamantyl_), 36.3 (3 CH_2adamantyl_), 37.5 (NCH_2_), 42.1 (3CH_2adamantyl_), 55.5 (C_adamantyl_), 109.4 (C-4a_carbazole_), 110.5 (C-1_carbazole_), 116.2 (C-8_carbazole_), 118.9 (C-8_chromone_), 119.6 (C-3_chromone_), 120.1 (C-6_carbazole_), 120.6 (C-7_carbazole_), 121.4 (C-4a_chromone_), 121.5 (C-5_carbazole_), 122.1 (C-4_carbazole_), 123.2 (C-2_carbazole_), 125.3 (C-5_chromone_), 126.0 (C-4b_carbazole_), 126.6 (=CH), 128.1 (C-5_thiazolidinone_), 128.4 (C-8a_carbazole_), 129.6 (C-7_chromone_), 135.8 (C-6_chromone_), 139.3 (C-9a_carbazole_), 141.4 (C-3_carbazole_), 154.9 (C-8a_chromone_), 155.8 (C-2_chromone_), 162.7 (C-2_thiazolidinone_), 165.5 (C-4_thiazolidinone_), 176.9 (C-4_chromone_). MS (*m*/*z*, I%): 613 (M^+^, 11%). Anal. calcd for C_38_H_35_N_3_O_3_S (613.78): C, 74.36%; H, 5.75%; N, 6.85%; S, 5.22%. Found: C, 74.14%; H, 5.65%; N, 6.68%; S, 5.09%.

### 2-[(9-Ethyl-9*H*-carbazol-3-yl)imino]-5-[(6-methyl-4-oxo-4*H*-chromen-3-yl)methylene]-3-phenyl-1,3-thiazolidin-4-one (4d)

Yellow solid in 93% yield, mp 216–218 °C, IR (KBr), (*v*_max_, cm^−1^): 3055 (C–H_arom_), 2979, 2914, 2852 (C–H_aliph_), 1719 (C=O_thiazolidinone_), 1624 (C=O_chromone_), 1595, 1560 (C=N, C=C). ^1^H-NMR (400 MHz, DMSO-*d*_6_): 1.36 (t, 3H, *J* = 6.8 Hz, CH_3_), 2.42 (s, 3H, CH_3_), 4.50 (q, 2H, *J* = 7.2 Hz, NCH_2_), 6.89 (d, 1H, *J* = 7.2 Hz, Ph–H), 6.96 (d, 1H, *J* = 7.6 Hz, Ph–H), 7.15 (t, 1H, *J* = 7.2 Hz, H-8_chromone_), 7.25 (t, 1H, *J* = 6.8 Hz, H-7_chromone_), 7.37 (t, 1H, *J* = 7.2 Hz, H–carbazole), 7.49–7.68 (m, 7H, Ph–H, CH= and H–carbazole), 7.75 (d, 1H, *J* = 8.4 Hz, H–carbazole), 7.91 (s, 1H, H-5_chromone_), 8.19 (d, 1H, *J* = 7.6 Hz, H–carbazole), 8.30 (d, 1H, *J* = 2.0 Hz, H–carbazole), 8.84 (s, 1H, H-2_chromone_). ^13^C-NMR (100 MHz, DMSO-*d*_6_): 14.8 (CH_3_), 21.4 (CH_3_), 38.4 (NCH_2_), 109.6 (C-4a_carbazole_), 111.1 (C-1_carbazole_), 118.6 (C-8_chromone_), 118.8 (C-8_carbazole_), 119.6 (C-3_chromone_), 121.1 (C-6_carbazole_), 122.3 (C-4_carbazole_), 122.7 (C-7_carbazole_), 123.1 (C-5_carbazole_), 123.5 (C-4a_chromone_), 123.9 (C-2_carbazole_), 125.3 (C-5_chromone_), 126.3 (C-4b_carbazole_), 126.6 (=CH), 127.4 (C-5_thiazolidinone_), 128.0 (C-2,6_phenyl_), 128.6 (C-3,5_phenyl_), 128.9 (C-8a_carbazole_), 129.6 (C-7_chromone_), 133.1 (C-4_phenyl_), 135.8 (C-6_chromone_), 139.6 (C-9a_carbazole_), 141.6 (C-1_phenyl_), 148.0 (C-3_carbazole_), 154.4 (C-8a_chromone_), 154.6 (C-2_chromone_), 160.7 (C-2_thiazolidinone_), 166.5 (C-4_thiazolidinone_), 175.7 (C-4_chromone_). MS (*m*/*z*, I%): 555 (M^+^, 16%). Anal. calcd for C_34_H_25_N_3_O_3_S (555.65): C, 73.49%; H, 4.54%; N, 7.56%; S, 5.77%. Found: C, 73.11%; H, 4.31%; N, 7.34%; S, 5.59%.

### 3-(4-Chlorophenyl)-2-[(9-ethyl-9*H*-carbazol-3-yl)imino]-5-[(6-methyl-4-oxo-4*H*-chromen-3-yl)methylene]-1,3-thiazolidin-4-one (4e)

Yellow solid in 92% yield, mp 275–276 °C, IR (KBr), (*v* max, cm^−1^): 3056 (C–H_arom_), 2983 (C–H_aliph_), 1707 (C=O_thiazolidinone_), 1654 (C=O_chromone_), 1618, 1598, 1556 (C=N, C=C). ^1^H-NMR (400 MHz, DMSO-*d*_6_): 1.34 (t, 3H, *J* = 7.2 Hz, CH_3_), 2.41 (s, 3H, CH_3_), 4.48 (q, 2H, *J* = 7.2 Hz, NCH_2_), 6.99 (d, 2H, *J* = 8.4 Hz, Ar–H), 7.22 (t, 1H, *J* = 7.2 Hz, H-8_chromone_), 7.41 (d, 2H, *J* = 8.4 Hz, Ar–H), 7.49 (t, 1H, *J* = 8.0 Hz, H-7_chromone_), 7.54–7.65 (m, 5H, CH= and H–carbazole), 7.72 (d, 1H, *J* = 8.4 Hz, H–carbazole), 7.89 (s, 1H, H-5_chromone_), 8.15 (d, 1H, *J* = 8.0 Hz, H–carbazole), 8.28 (s, 1H, H–carbazole), 8.83 (s, 1H, H-2_chromone_). ^13^C-NMR (100 MHz, DMSO-*d*_6_): 14.2 (CH_3_), 20.9 (CH_3_), 37.6 (NCH_2_), 109.9 (C-4a_carbazole_), 112.2 (C-1_carbazole_), 118.6 (C-8_chromone_), 118.8 (C-8_carbazole_), 119.6 (C-3_chromone_), 121.1 (C-6_carbazole_), 122.3 (C-4_carbazole_), 122.7 (C-7_carbazole_), 123.1 (C-5_carbazole_), 123.3 (C-3,5_aryl_), 123.5 (C-4a_chromone_), 123.9 (C-2_carbazole_), 125.3 (C-5_chromone_), 126.3 (C-4b_carbazole_), 126.6 (=CH), 126.7 (C-5_thiazolidinone_), 128.9 (C-8a_carbazole_), 129.6 (C-7_chromone_),129.8 (C-2,6_aryl_), 136.5 (C-6_chromone_), 136.6 (C-4_aryl_), 139.6 (C-9a_carbazole_), 140.5 (C-1_aryl_), 147.6 (C-3_carbazole_), 154.1 (C-8a_chromone_), 154.2 (C-2_chromone_), 161.2 (C-2_thiazolidinone_), 166.8 (C-4_thiazolidinone_), 175.3 (C-4_chromone_). MS (*m*/*z*, I%): 589 (M^+^, 31%), 591 (M+2, 14%). Anal. calcd for C_34_H_24_ClN_3_O_3_S (590.09): C, 69.20%; H, 4.10%; N, 7.12%; S, 5.43%. Found: C, 69.06%; H, 3.99%; N, 7.01%; S, 5.28%.

### Synthesis of 2-{[(9-ethyl-9*H*-carbazol-3-yl)imino]-5-[(3-(2-hydroxy-5-methylphenyl)-1*H*-pyrazol-4-yl)]methylene}-3-substituted-1,3-thiazolidin-4-ones (5a–e)

A mixture of each one of the isolated products 4a–e (2.5 mmol) and hydrazine hydrate 99% (3.0 mmol, 0.15 ml) was dissolved in absolute ethanol (25 ml) and irradiated under ultrasonication waves for 20 min at 50 °C. The formed solid on heating was filtrated off and crystallized from a mixture of DMF : EtOH (1 : 1).

### 2-[(9-Ethyl-9*H*-carbazol-3-yl)imino]-5-{[3-(2-hydroxy-5-methylphenyl)-1*H*-pyrazol-4-yl] methylene}-3-methyl-1,3-thiazolidin-4-one (5a)

Yellow solid in 88% yield, mp 271–273 °C, IR (KBr), (*v*_max_, cm^−1^): 3264 (OH), 3102 (NH), 3055 (C–H_arom_), 2984, 2902 (C–H_aliph_), 1688 (C=O_thiazolidinone_), 1636 (C=C_exocyclic_), 1604, 1557 (C=N, C=C). ^1^H-NMR (400 MHz, DMSO-*d*_6_): 1.36 (t, 3H, *J* = 7.2 Hz, CH_3_), 2.23 (s, 3H, CH_3_), 3.36 (s, 3H, CH_3_), 4.46 (q, 2H, *J* = 6.8 Hz, NCH_2_), 6.91 (d, 1H, *J* = 7.6 Hz, Ar–H), 7.03 (s, 1H, Ar–H), 7.11 (d, 1H, *J* = 5.2 Hz, Ar–H), 7.16–7.21 (m, 2H, H–carbazole), 7.45–7.49 (m, 2H, CH= and H–carbazole), 7.60–7.66 (m, 3H, H–carbazole), 7.83 (d, 1H, *J* = 2.0 Hz, H-5_pyrazole_), 8.16 (d, 1H, *J* = 7.6 Hz, H–carbazole), 9.79 (brs, 1H, NH), 13.37 (brs, 1H, OH). ^13^C-NMR (100 MHz, DMSO-*d*_6_): 14.2 (CH_3_), 20.3 (CH_3_), 30.1 (CH_3_), 37.5 (NCH_2_), 109.2 (C-4a_carbazole_), 110.1 (C-1_carbazole_), 112.6 (C-3_aryl_), 114.6 (C-4_pyrazole_), 119.1 (C-8_carbazole_), 120.1 (C-1_aryl_), 121.1 (C-6_carbazole_), 121.6 (C-2_carbazole_), 122.2 (C-4_carbazole_), 122.4 (C-7_carbazole_), 123.2 (C-5_carbazole_), 126.5 (C-4b_carbazole_), 126.9 (=CH), 127.5 (C-5_thiazolidinone_), 128.3 (C-8a_carbazole_), 131.6 (C-4_aryl_), 131.8 (C-5_aryl_), 131.9 (C-6_aryl_), 137.5 (C-9a_carbazole_), 140.3 (C-5_pyrazole_), 140.5 (C-3_carbazole_), 149.8 (C-3_pyrazole_), 153.2 (C-2_aryl_), 162.7 (C-2_thiazolidinone_), 166.1 (C-4_thiazolidinone_). MS (*m*/*z*, I%): 507 (M^+^, 29%). Anal. calcd for C_29_H_25_N_5_O_2_S (507.61): C, 68.62%; H, 4.96%; N, 13.80%; S, 6.32%. Found: C, 68.49%; H, 4.83%; N, 13.65%; S, 6.19%.

### 3-Allyl-2-[(9-ethyl-9*H*-carbazol-3-yl)imino]-5-{[3-(2-hydroxy-5-methylphenyl)-1*H*-pyrazol-4-yl]methylene}-1,3-thiazolidin-4-one (5b)

Yellow solid in 88% yield, mp 243–245 °C, IR (KBr), (*v*_max_, cm^−1^): 3256 (OH), 3166 (NH), 3032 (C–H_arom_), 2966, 2929 (C–H_aliph_), 1695 (C=O_thiazolidinone_), 1630 (C=C_exocyclic_), 1602, 1573 (C=N, C=C). ^1^H-NMR (400 MHz, DMSO-*d*_6_): 1.36 (t, 3H, *J* = 7.2 Hz, CH_3_), 2.24 (s, 3H, CH_3_), 4.46 (q, 2H, *J* = 7.2 Hz, NCH_2_), 4.53 (d, 2H, *J* = 5.2 Hz, CH_2_), 5.25 (dd, 2H, *J* = 6.4 and 1.2 Hz, =CH_2_), 5.95–6.04 (m, 1H, CH=), 6.90 (d, 1H, *J* = 8.4 Hz, Ar–H), 7.03 (s, 1H, Ar–H), 7.11 (d, 1H, *J* = 8.0 Hz, Ar–H), 7.15–7.21 (m, 2H, H–carbazole), 7.45–7.49 (m, 2H, CH= and H–carbazole), 7.61–7.66 (m, 3H, H–carbazole), 7.83 (d, 1H, *J* = 2.4 Hz, H-5_pyrazole_), 8.17 (d, 1H, *J* = 7.6 Hz, H–carbazole), 9.77 (brs, 1H, NH), 13.36 (brs, 1H, OH). ^13^C-NMR (100 MHz, DMSO-*d*_6_): ^13^C-NMR (100 MHz, DMSO-*d*_6_): 14.2 (CH_3_), 20.3 (CH_3_), 37.5 (NCH_2_), 45.0 (CH_2_), 109.6 (C-4a_carbazole_), 110.1 (C-1_carbazole_), 112.6 (C-3_aryl_), 114.6 (C-4_pyrazole_), 116.4 (=CH_2_), 119.1 (C-8_carbazole_), 120.1 (C-1_aryl_), 121.1 (C-6_carbazole_), 121.4 (C-4_carbazole_), 122.2 (C-2_carbazole_), 122.4 (C-7_carbazole_), 123.2 (C-5_carbazole_), 125.4 (C-4b_carbazole_), 126.5 (=CH), 128.4 (C-8a_carbazole_), 129.8 (C-5_thiazolidinone_), 131.5 (C-4_aryl_), 131.8 (C-5_aryl_), 131.9 (C-6_aryl_), 137.5 (C-9a_carbazole_), 140.3 (=CH), 140.5 (C-3_carbazole_), 140.6 (C-5_pyrazole_), 149.8 (C-3_pyrazole_), 153.2 (C-2_aryl_), 162.7 (C-2_thiazolidinone_), 166.4 (C-4_thiazolidinone_). MS (*m*/*z*, I%): 533 (M^+^, 31%). Anal. calcd for C_31_H_27_N_5_O_2_S (533.65): C, 69.77%; H, 5.10%; N, 13.12%; S, 6.01%. Found: C, 69.61%; H, 4.96%; N, 13.02%; S, 5.86%.

### 3-(Adamantan-1-yl)-2-[(9-ethyl-9*H*-carbazol-3-yl)imino]-5-{[3-(2-hydroxy-5-methylphenyl)-1*H*-pyrazol-4-yl]methylene}-1,3-thiazolidin-4-one (5c)

Yellow solid in 89% yield, mp 285–287 °C, IR (KBr), (*v* max, cm^−1^): 3319 (OH), 3232 (NH), 3048 (C–H_arom_), 2978, 2918, 2851 (C–H_aliph_), 1689 (C=O_thiazolidinone_), 1646 (C=C_exocyclic_), 1611, 1558 (C=N, C=C). ^1^H-NMR (400 MHz, DMSO-*d*_6_): 1.35 (t, 3H, *J* = 7.2 Hz, CH_3_), 1.59–1.68 (m, 6H, CH_2_), 1.79–1.85 (m, 6H, CH_2_), 2.00–2.05 (m, 3H, CH_2_), 2.25 (s, 3H, CH_3_), 4.46 (q, 2H, *J* = 7.2 Hz, NCH_2_), 6.90 (d, 1H, *J* = 8.0 Hz, Ar–H), 7.08 (s, 1H, Ar–H), 7.10 (d, 1H, *J* = 8.4 Hz, Ar–H), 7.21 (t, 1H, *J* = 8.0 Hz, H–carbazole), 7.15–7.21 (dd, 1H, *J* = 8.4 and 1.6 Hz, H–carbazole), 7.44–7.49 (m, 2H, CH= and H–carbazole), 7.62–7.65 (m, 2H, H–carbazole), 8.04 (d, 1H, *J* = 2.0 Hz, H-5_pyrazole_), 8.09 (s, 1H, H–carbazole), 8.14 (d, 1H, *J* = 7.6 Hz, H–carbazole), 10.19 (brs, 2H, NH and OH). ^13^C-NMR (100 MHz, DMSO-*d*_6_): 14.1 (CH_3_), 20.4 (CH_3_), 29.5 (3 CH_adamantyl_), 36.2 (3 CH_2adamantyl_), 42.2 (3 CH_2adamantyl_), 37.5 (NCH_2_), 55.5 (C_adamantyl_), 109.2 (C-4a_carbazole_), 109.6 (C-1_carbazole_), 112.2 (C-3_aryl_), 114.8 (C-4_pyrazole_), 116.4 (C-8_carbazole_), 119.4 (C-1_aryl_), 120.8 (C-6_carbazole_), 120.9 (C-7_carbazole_), 121.5 (C-5_carbazole_), 122.3 (C-4_carbazole_), 122.4 (C-2_carbazole_), 126.4 (C-4b_carbazole_), 126.6 (=CH), 127.7 (C-5_thiazolidinone_), 128.2 (C-8a_carbazole_), 130.8 (C-4_aryl_), 131.5 (C-5_aryl_), 131.8 (C-6_aryl_), 139.1 (C-9a_carbazole_), 140.2 (C-5_pyrazole_), 141.3 (C-3_carbazole_), 146.5 (C-3_pyrazole_), 153.3 (C-2_aryl_), 162.7 (C-2_thiazolidinone_), 165.6 (C-4_thiazolidinone_). MS (*m*/*z*, I%): 627 (M^+^, 16%). Anal. calcd for C_38_H_37_N_5_O_2_S (627.81): C, 72.70%; H, 5.94%; N, 11.16%; S, 5.11%. Found: C, 72.59%; H, 5.82%; N, 11.02%; S, 5.01%.

### 2-[(9-Ethyl-9*H*-carbazol-3-yl)imino]-5-{[3-(2-hydroxy-5-methylphenyl)-1*H*-pyrazol-4-yl] methylene}-3-phenyl-1,3-thiazolidin-4-one (5d)

Yellow solid in 92% yield, mp 270–271 °C, IR (KBr), (*v*_max_, cm^−1^): 3555 (OH), 3143 (NH), 3055 (C–H_arom_), 2967, 2937 (C–H_aliph_), 1704 (C=O_thiazolidinone_), 1645 (C=C_exocyclic_), 1612, 1592 (C=N, C=C). ^1^H-NMR (400 MHz, DMSO-*d*_6_): 1.35 (t, 3H, *J* = 7.2 Hz, CH_3_), 2.24 (s, 3H, CH_3_), 4.46 (q, 2H, *J* = 7.2 Hz, NCH_2_), 6.90 (d, 1H, *J* = 8.4 Hz, Ar–H), 6.99 (d, 2H, *J* = 7.6 Hz, Ph–H), 7.06 (s, 1H, Ar–H), 7.11 (d, 1H, *J* = 8.0 Hz, Ar–H), 7.21 (t, 1H, *J* = 7.6 Hz, H–carbazole), 7.24 (t, 1H, *J* = 7.6 Hz, Ph–H), 7.39 (t, 2H, *J* = 8.0 Hz, Ph–H), 7.48–7.55 (m, 2H, H–carbazole), 7.57 (s, 1H, CH=), 7.66 (d, 1H, *J* = 8.0 Hz, H–carbazole), 7.74 (d, 1H, *J* = 8.4 Hz, H–carbazole), 7.82 (s, 1H, H–carbazole), 8.18 (d, 1H, *J* = 8.0 Hz, H–carbazole), 8.27 (d, 1H, *J* = 2.0 Hz, H–5_pyrazole_), 10.21 (brs, 2H, NH and OH). ^13^C-NMR (100 MHz, DMSO-*d*_6_): 14.6 (CH_3_), 20.7 (CH_3_), 36.2 (NCH_2_), 108.7 (C-4a_carbazole_), 110.8 (C-1_carbazole_), 112.2 (C-3_aryl_), 115.6 (C-4_pyrazole_), 119.4 (C-8_carbazole_), 120.1 (C-1_aryl_), 121.5 (C-6_carbazole_), 122.0 (C-4_carbazole_), 122.3 (C-7_carbazole_), 122.4 (C-5_carbazole_), 123.9 (C-2_carbazole_), 126.4 (C-4b_carbazole_), 126.6 (=CH), 127.7 (C-5_thiazolidinone_), 128.2 (C-2,6_phenyl_), 128.9 (C-8a_carbazole_), 129.4 (C-3,5_phenyl_), 130.1 (C-4_phenyl_), 131.0 (C-4_aryl_), 131.8 (C-5_aryl_), 132.2 (C-6_aryl_), 139.3 (C-1_phenyl_), 139.5 (C-9a_carbazole_), 140.3 (C-5_pyrazole_), 140.9 (C-3_carbazole_), 148.2 (C-3_pyrazole_), 152.9 (C-2_aryl_), 161.7 (C-2_thiazolidinone_), 165.1 (C-4_thiazolidinone_). MS (*m*/*z*, I%): 569 (M^+^, 22%). Anal. calcd for C_34_H_27_N_5_O_2_S (569.68): C, 71.68%; H, 4.78%; N, 12.29%; S, 5.63%. Found: C, 71.52%; H, 4.62%; N, 12.11%; S, 5.51%.

### 3-(4-Chlorophenyl)-2-[(9-ethyl-9*H*-carbazol-3-yl)imino]-5-{[3-(2-hydroxy-5-methylphenyl)-1*H*-pyrazol-4-yl]methylene}-1,3-thiazolidin-4-one (5e)

Beige solid in 90% yield, mp > 300 °C IR (KBr), (*v*_max_, cm^−1^): 3330 (OH), 3162 (NH), 3044 (C–H_arom_), 2982, 2938 (C–H_aliph_), 1699 (C=O_thiazolidinone_), 1642 (C=C_exocyclic_), 1622, 1589 (C=N, C=C). ^1^H-NMR (400 MHz, DMSO-*d*_6_): 1.35 (t, 3H, *J* = 7.2 Hz, CH_3_), 2.24 (s, 3H, CH_3_), 4.50 (q, 2H, *J* = 6.8 Hz, NCH_2_), 6.90 (d, 1H, *J* = 8.0 Hz, Ar–H), 7.02 (d, 2H, *J* = 8.4 Hz, Ar–H), 7.07 (s, 1H, Ar–H), 7.11 (d, 1H, *J* = 8.4 Hz, Ar–H), 7.24 (t, 1H, *J* = 7.6 Hz, H–carbazole), 7.43 (d, 2H, *J* = 8.4 Hz, Ar–H), 7.48–7.55 (m, 2H, H–carbazole), 7.59 (s, 1H, CH=), 7.65 (d, 1H, *J* = 8.4 Hz, H–carbazole), 7.73 (d, 1H, *J* = 8.4 Hz, H–carbazole), 7.88 (s, 1H, H–carbazole), 8.17 (d, 1H, *J* = 7.6 Hz, H–carbazole), 8.27 (d, 1H, *J* = 1.6 Hz, H-5_pyrazole_), 9.97 (brs, 1H, NH), 12.75 (brs, 1H, OH). ^13^C-NMR (100 MHz, DMSO-*d*_6_): 14.1 (CH_3_), 20.4 (CH_3_), 37.6 (NCH_2_), 109.8 (C-4a_carbazole_), 110.4 (C-1_carbazole_), 113.0 (C-3_aryl_), 114.6 (C-4_pyrazole_), 116.5 (C-8_carbazole_), 119.6 (C-1_aryl_), 122.3 (C-6_carbazole_), 122.4 (C-4_carbazole_), 121.1 (C-7_carbazole_), 122.7 (C-5_carbazole_), 123.3 (C-3,5_aryl`_), 124.8 (C-2_carbazole_), 126.3 (C-4b_carbazole_), 126.7 (=CH), 128.3 (C-5_thiazolidinone_), 129.8 (C-2,6_aryl`_), 129.2 (C-8a_carbazole_), 
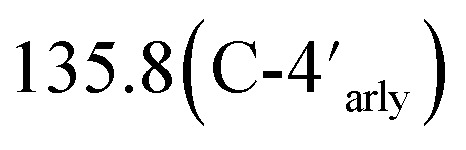
, 131.1 (C-4_aryl_), 131.6 (C-5_aryl_), 131.9 (C-6_aryl_), 140.0 (C-1_aryl`_), 139.5 (C-9a_carbazole_), 140.5 (C-5_pyrazole_), 141.2 (C-3_carbazole_), 147.5 (C-3_pyrazole_), 153.3 (C-2_aryl_), 161.9 (C-2_thiazolidinone_), 166.6 (C-4_thiazolidinone_). MS (*m*/*z*, I%): 603 (M^+^, 57%), 605 (M+2, 19%). Anal. calcd for C_34_H_26_ClN_5_O_2_S (604.13): C, 67.60%; H, 4.34%; N, 11.59%; S, 5.31%. Found: C, 67.49%; H, 4.21%; N, 11.42%; S, 5.24%.

### *In vitro* cytotoxicity

The American type of culture collection (ATCC) provided human cell lines for human prostate cancer cell line (PC3), liver cancer cells (HepG2), and colon cancer cells (HCT116). A humidified, 5% (v/v) CO_2_ atmosphere was used to culture the cells at 37 °C in RPMI-1640 supplemented with (100 μg ml^−1^); penicillin (100 units per ml); and heat-inactivated fetal bovine serum (10% v/v). Using the sulforhodamine B (SRB) assay, the cytotoxicity of the synthesized compounds against (PC3, HepG2, and HCT116) human tumor cells was assessed. Before being treated with the synthesized compounds, cells growing at 80% confluency, trypsinized and cultured in a 96-well tissue culture plate for 24 h. Cells were subjected to six different doses of each chemical (0.01, 0.1, 1, 10, and 1000 μg ml^−1^), with untreated cells added as a control. Before the cells were fixed with TCA (10% w/v) for an hour at 4 °C, they were exposed to the concentrations for 72 h. After multiple washings, cells were stained with a 0.4% (w/v) SRB solution for 10 min in the dark. The surplus stain was eliminated using 1% (v/v) glacial acetic acid. The SRB-stained cells were dissolved in Tris–HCl buffer after drying overnight. A microplate reader was used to gauge the color intensity at 540 nm. Sigma Plot 12.0 software was used to examine the association between each tumor cell line's viability percentage and compound concentrations to determine the IC_50_ (drug dose that reduces survival to 50%).^51,52^

### Molecular docking

The bioactive compounds were subject to docking study to explore their binding mode towards vascular endothelial growth factor kinase insert domain receptor (KDR) (PDB ID: 3EWH) protein which was downloaded from protein data bank. The ligand and receptor were prepared for docking with rigid protein geometry using Auto Dock Tools version 1.5.6.^54–57^ The docking cavities were defined according to the interactions of protein with the co-crystalized ligands which are also used as reference ligands. The grid box with dimensions of 14 × 16  ×  14, with 1.0 Å spacing were placed to make the entire binding cavities involved. The co-crystalized ligands were redocked to the receptor to validate the docking parameters. Docking was performed using AutoDockVina.^58^ The 2D and 3D images were generated by Discovery Studio and Chimera.^59^

## Conclusion

We successfully reported to synthesize a novel series of polycyclic systems consisting of carbazole–thiazolidinone–chromone and carbazole–thiazolidinone–pyrazole hybrids as new promising antiproliferative agents. The suggested method was very effective and produced pure products in excellent yields, short times, and in simple laboratory method. Among all the tested products, both products compounds 5b (R = CH_2_–CH=CH_2_) and 5e (R = 4-ClC_6_H_4_) which belong to carbazole–thiazolidinone–pyrazole hybrids were found to be the most active against HCT116, PC3 and HepG2 cancer cells. These results were further confirmed by the molecular docking studies, which indicated that the three products have good binding with bind with VEGFR-2 receptor. These bioactive products require further biological studies in the hope that they will be effective and promising anticancer substances.

## Author contributions

Data curation: NMH and MAA. Formal analysis: TEA and SMA. Investigation: NMH, MAA, TEA and SMA. Writing – original draft: TEA, NMH and SMA. Conceptualization: TEA, NMH and SMA. Supervision: TEA. Resources: NMH, MAA, TEA and SMA. Software: TEA and NMH. Methodology: NMH and SMA. Writing – review & editing: TEA, NMH and SMA. All authors read and approved the final version of the manuscript.

## Conflicts of interest

Authors declare that they have no conflict of interest.

## Supplementary Material

RA-014-D4RA03188A-s001
