# Supplementary material for: Novel carbazolyl–thiazolyl–chromone and carbazolyl–thiazolyl–pyrazole hybrids: synthesis, cytotoxicity evaluation and molecular docking studies
Source: RSC Adv. 2024 May 28;14(24):17245–60. doi: 10.1039/d4ra03188a (PMC11132180; doi:10.1039/d4ra03188a)
Supplement: RA-014-D4RA03188A-s001 [file RA-014-D4RA03188A-s001.pdf]

Electronic Supplementary Material (ESI) for RSC Advances.
This journal is © The Royal Society of Chemistry 2024

Supplemental Material

Novel Carbazolyl-thiazolyl-chromone and Carbazolyl-thiazolyl-pyrazole Hybrids: Synthesis, Cytotoxicity Evaluation and Molecular Docking Studies

Noha M. Hassanin[1*], Tarik E. Ali[2,3], Mohammed A. Assiri[3], and Somaia M. Abdel-Kariem[1]

[1]Department of Chemistry, Faculty of Education, Ain Shams University, Cairo, Egypt

[2]Central Labs, King Khalid University, AlQuraa, Abha, Saudi Arabia

[3]Department of Chemistry, Faculty of Science, King Khalid University, AlQuraa, Abha, Saudi Arabia

*E-mail: noha-mohamed@edu.asu.edu.eg

Copies of IR, NMR and MS Spectra for all the synthesized Compounds

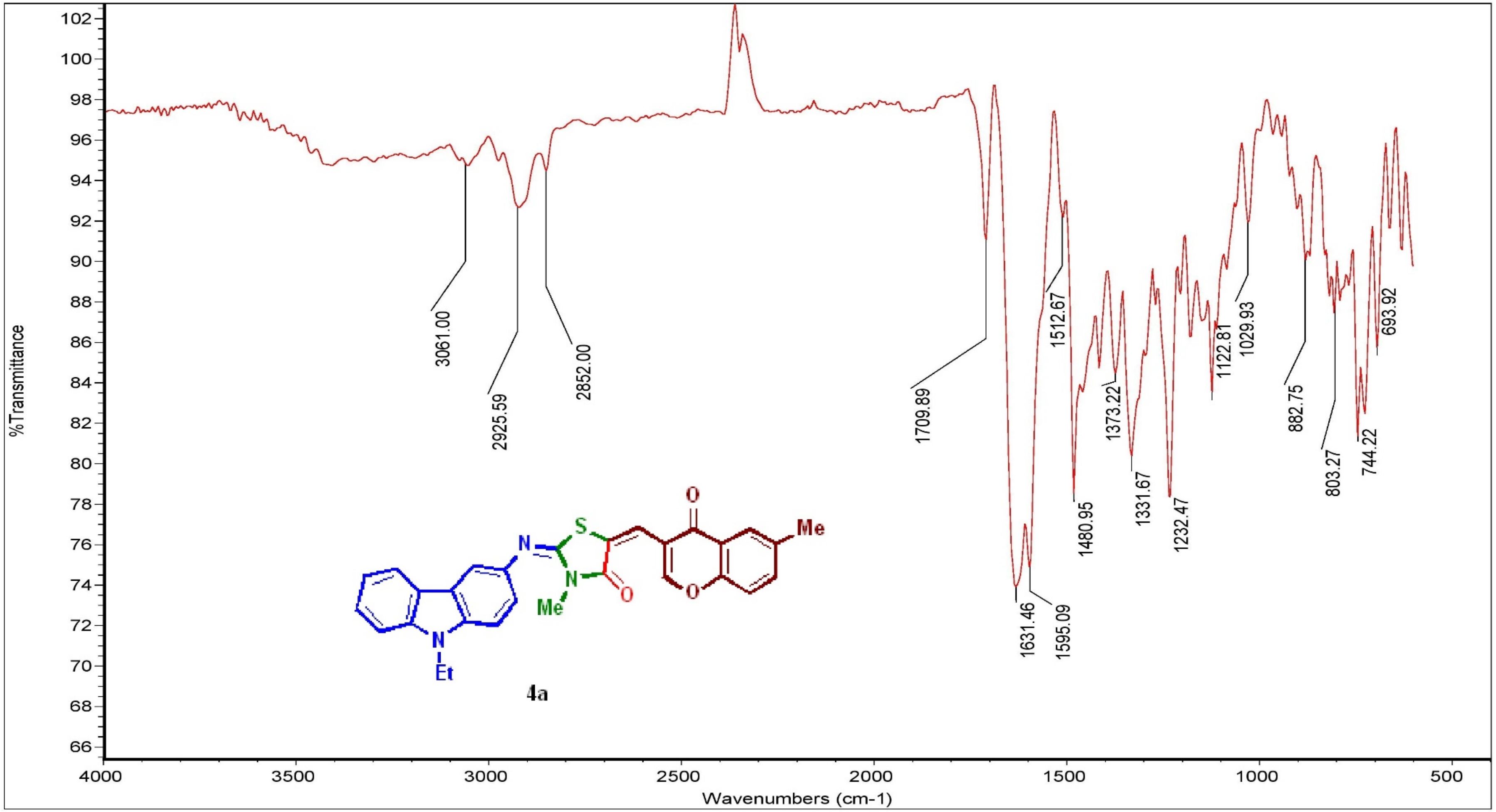

Figure S1: The IR spectrum of compound **4a**.

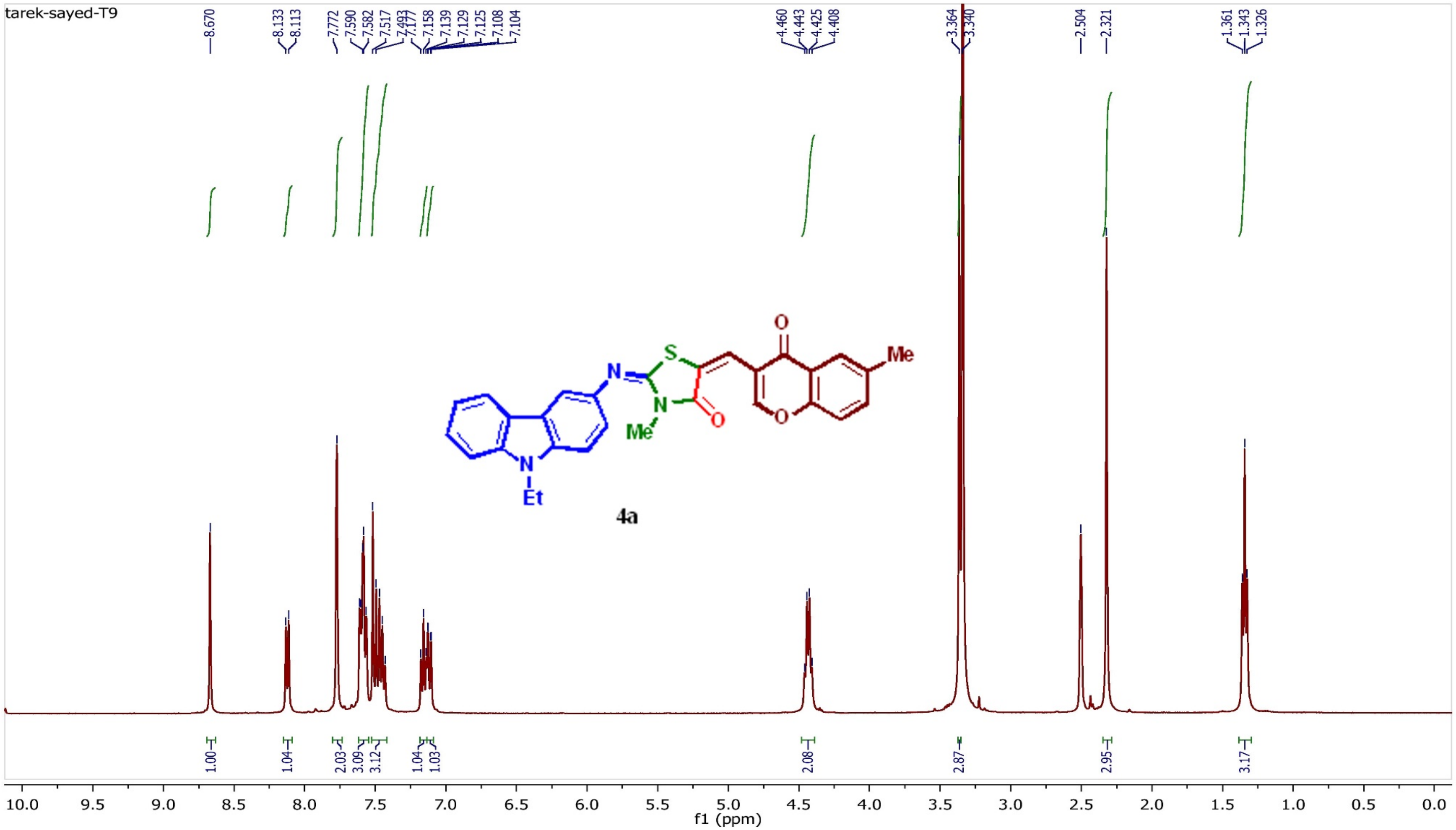

Figure S2: The ^{1}H-NMR spectrum of compound **4a**.

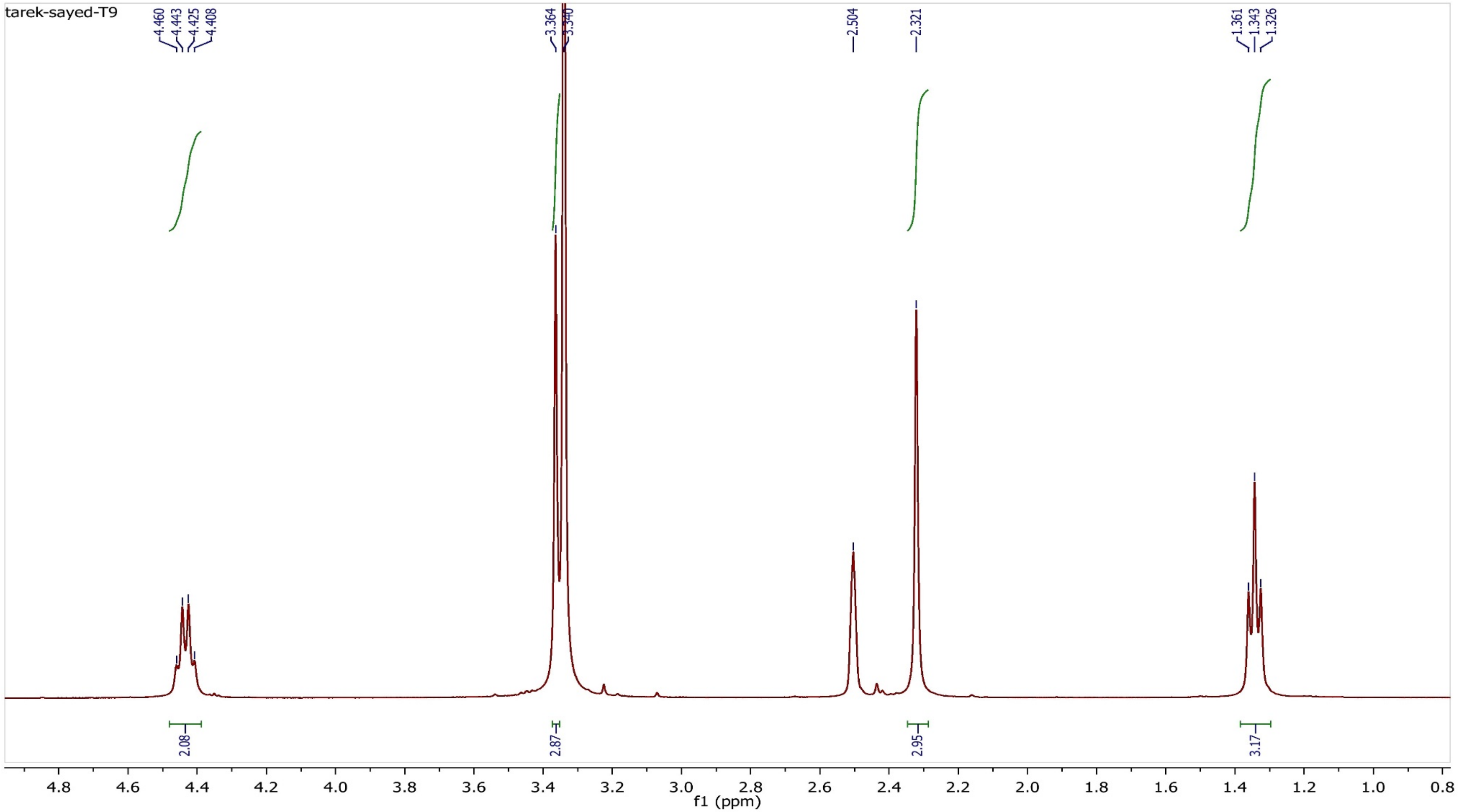

Figure S3: The enlargement of upfield region in ^{1}H-NMR spectrum of compound **4a**.

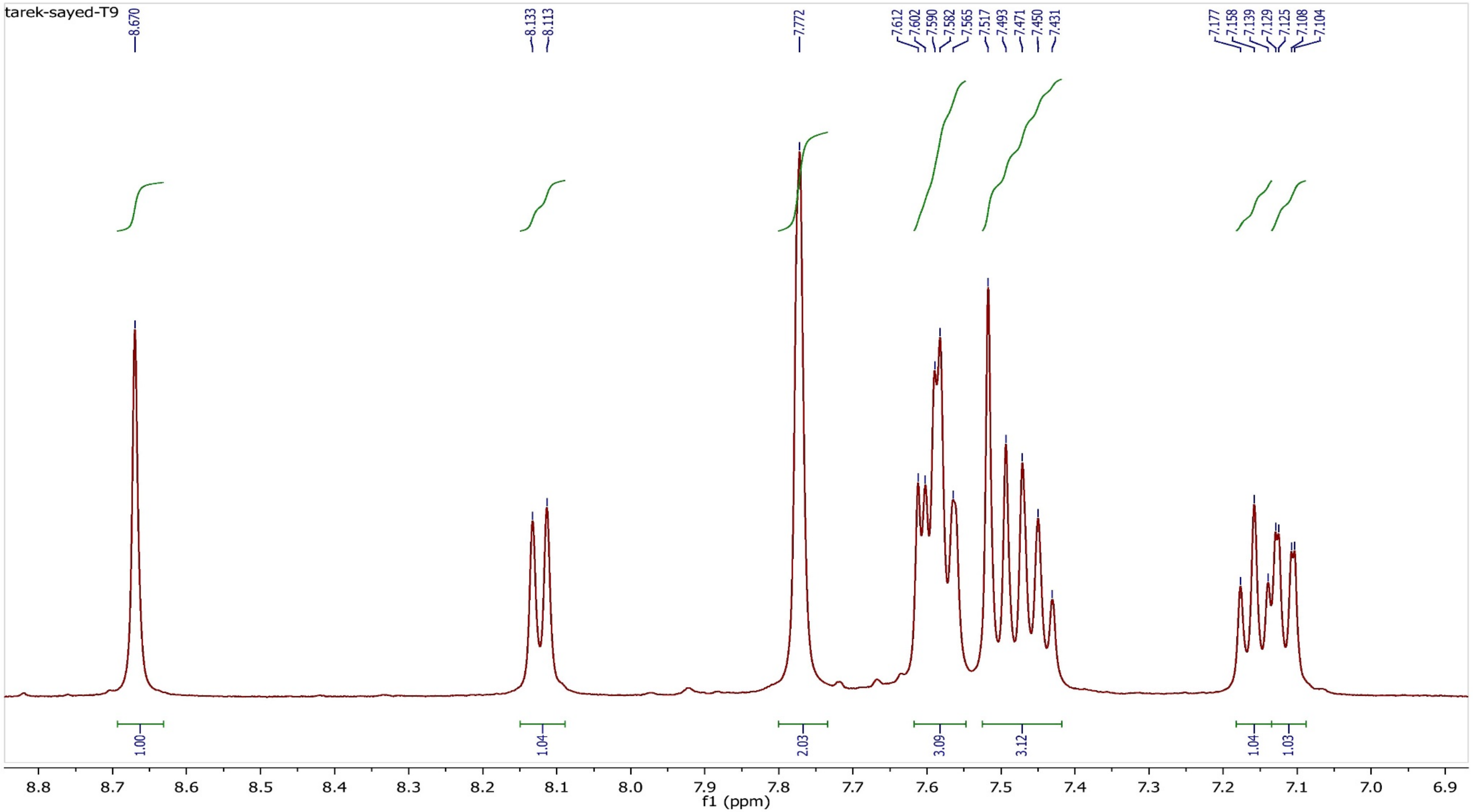

Figure S4: The enlargement of downfield region in ^{1}H-NMR spectrum of compound **4a**.

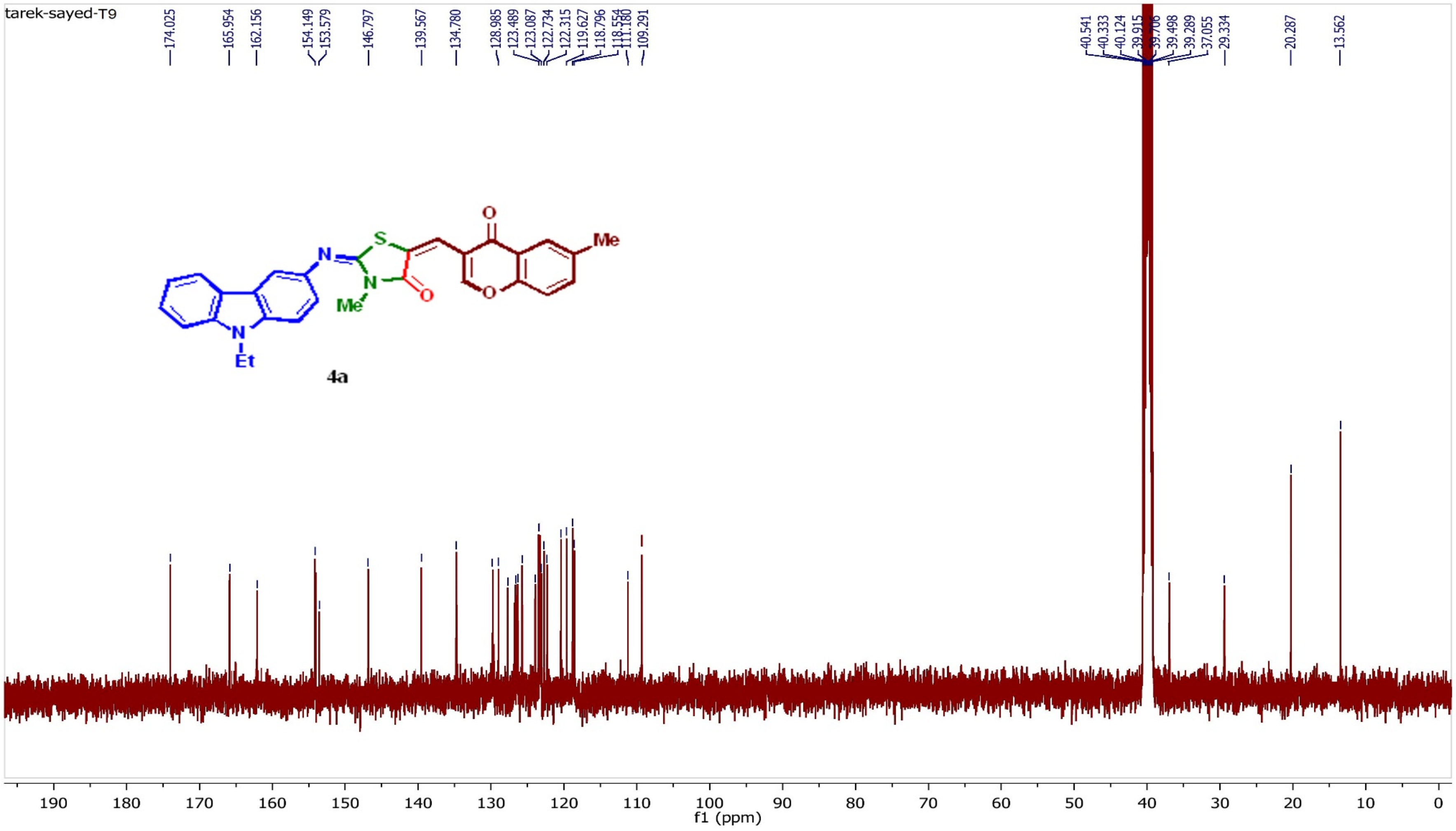

Figure S5: The ^{13}C-NMR spectrum of compound **4a**.

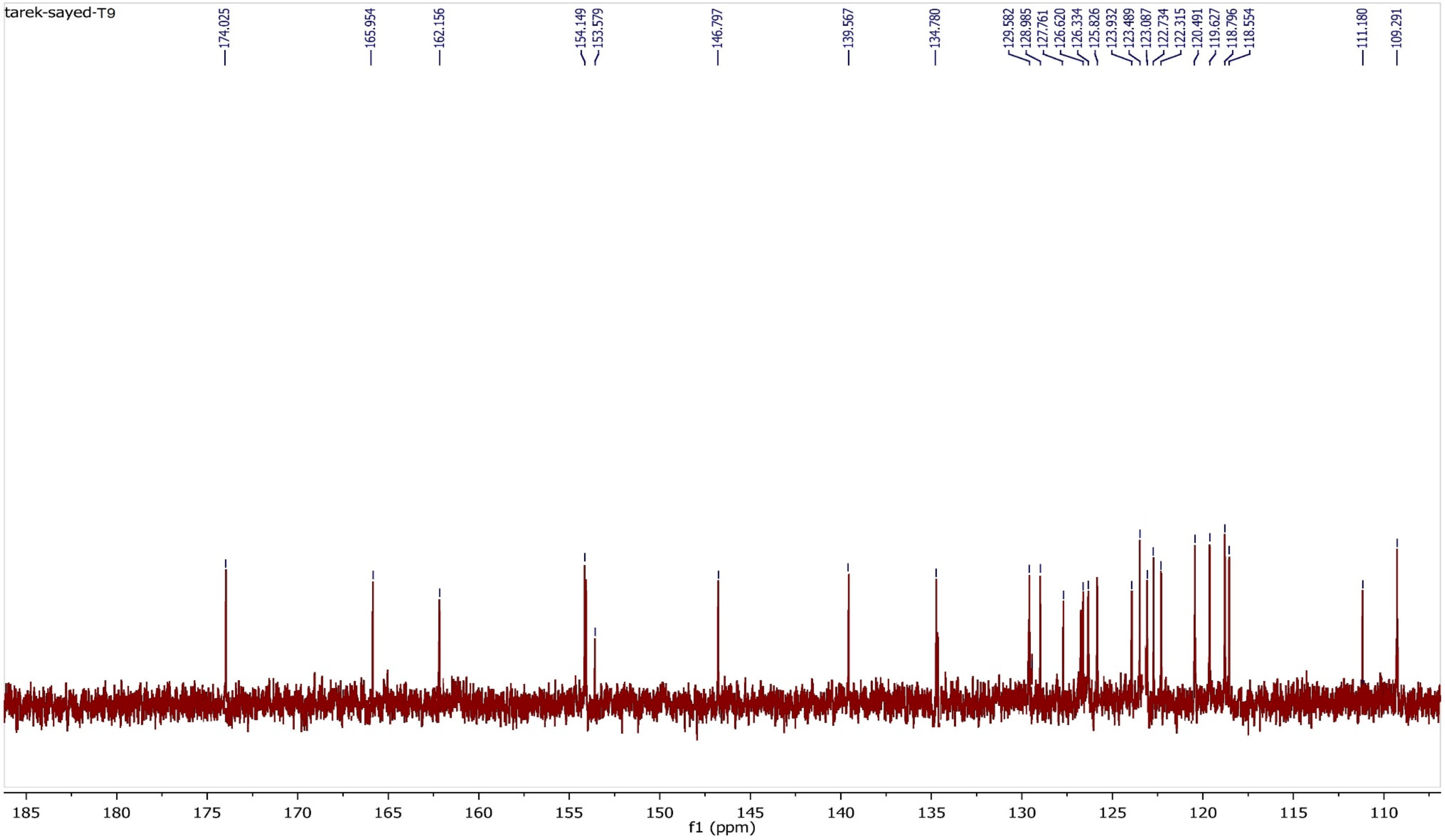

Figure S6: The enlargement of downfield region in ^{13}C-NMR spectrum of compound **4a**.

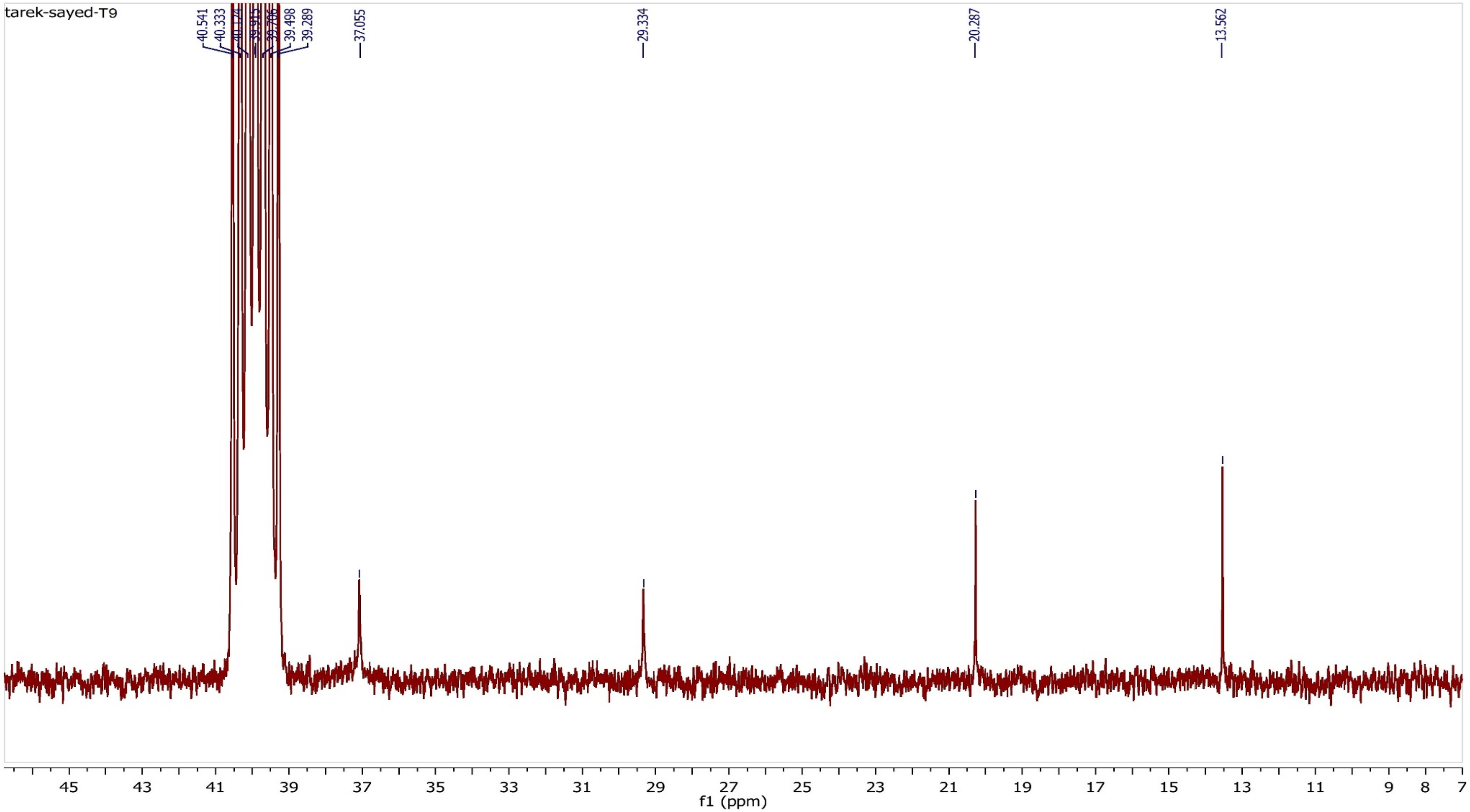

Figure S7: The enlargement of upfield region in ^{13}C-NMR spectrum of compound **4a**.

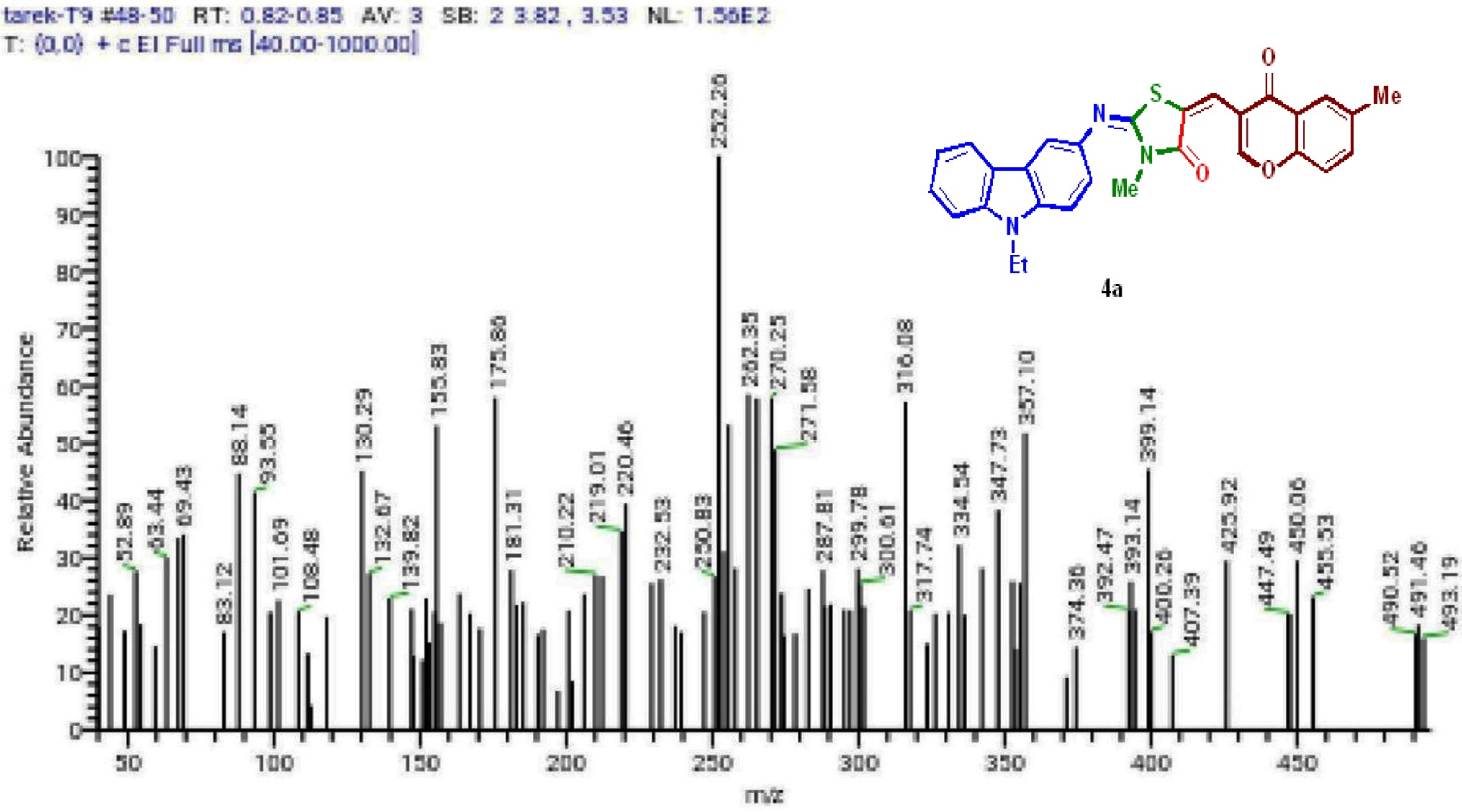

Figure S8: The mass spectrum of compound **4a**.

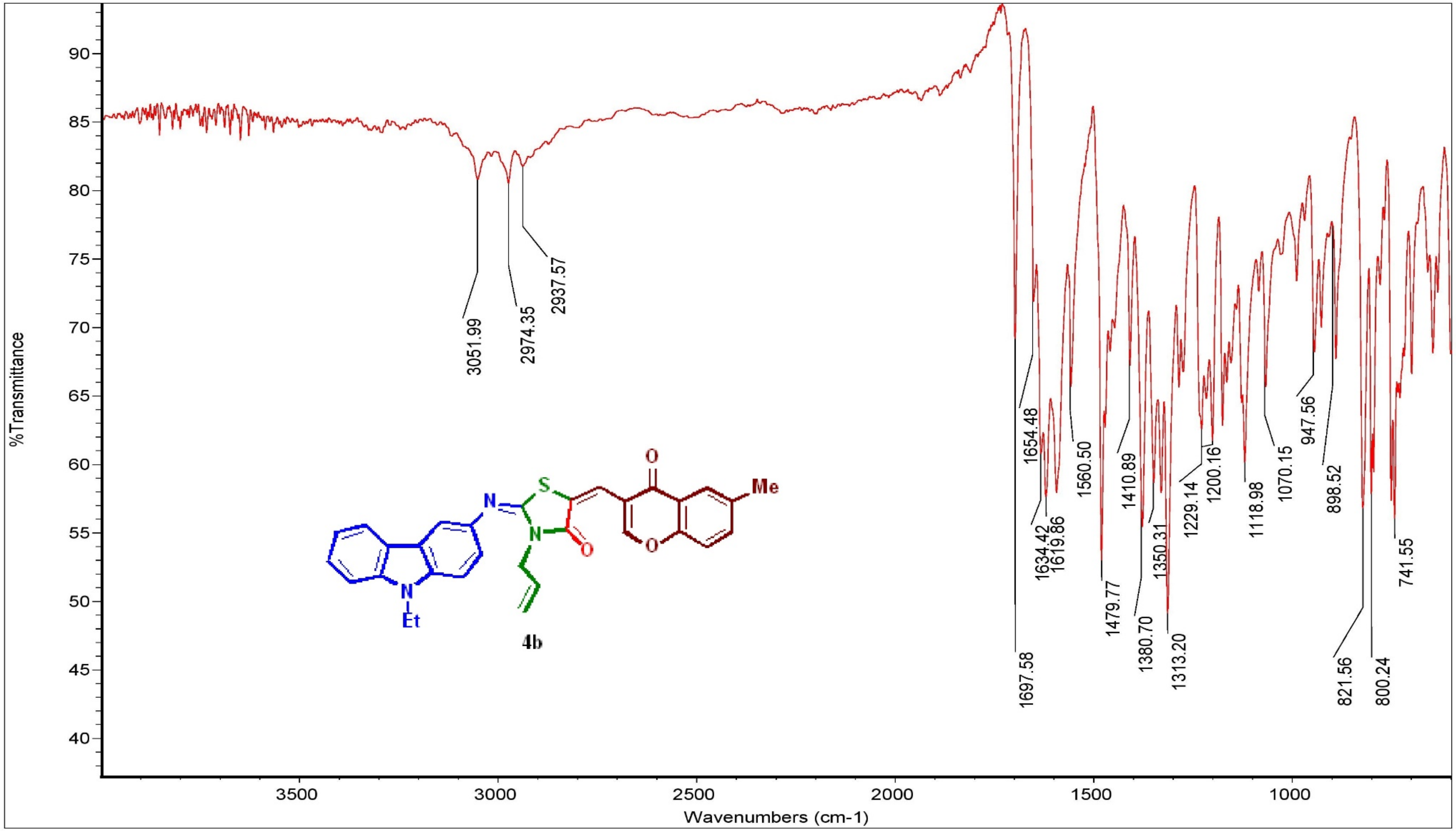

Figure S9: The IR spectrum of compound **4b**.

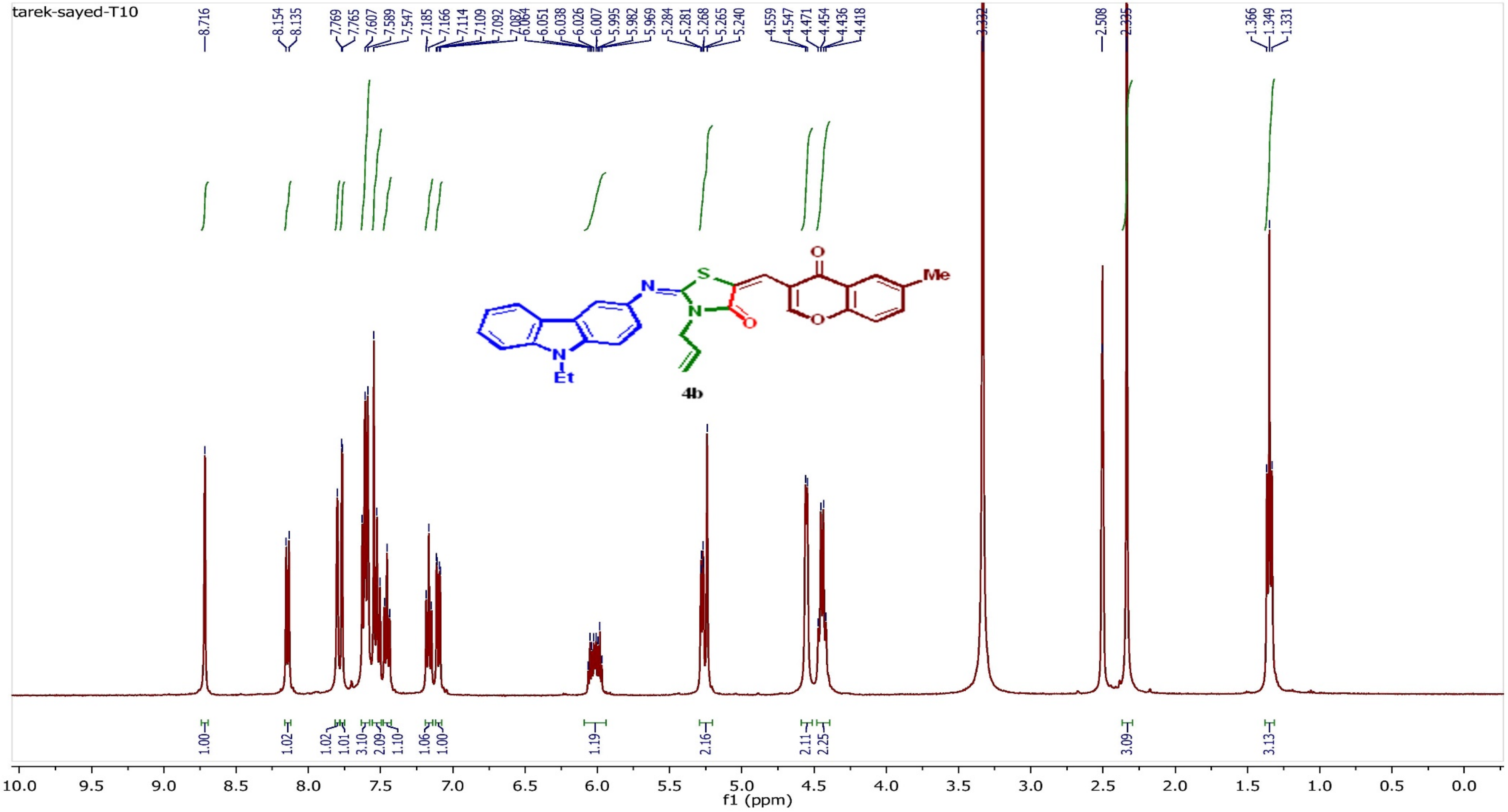

Figure S10: The ^{1}H-NMR spectrum of compound **4b**.

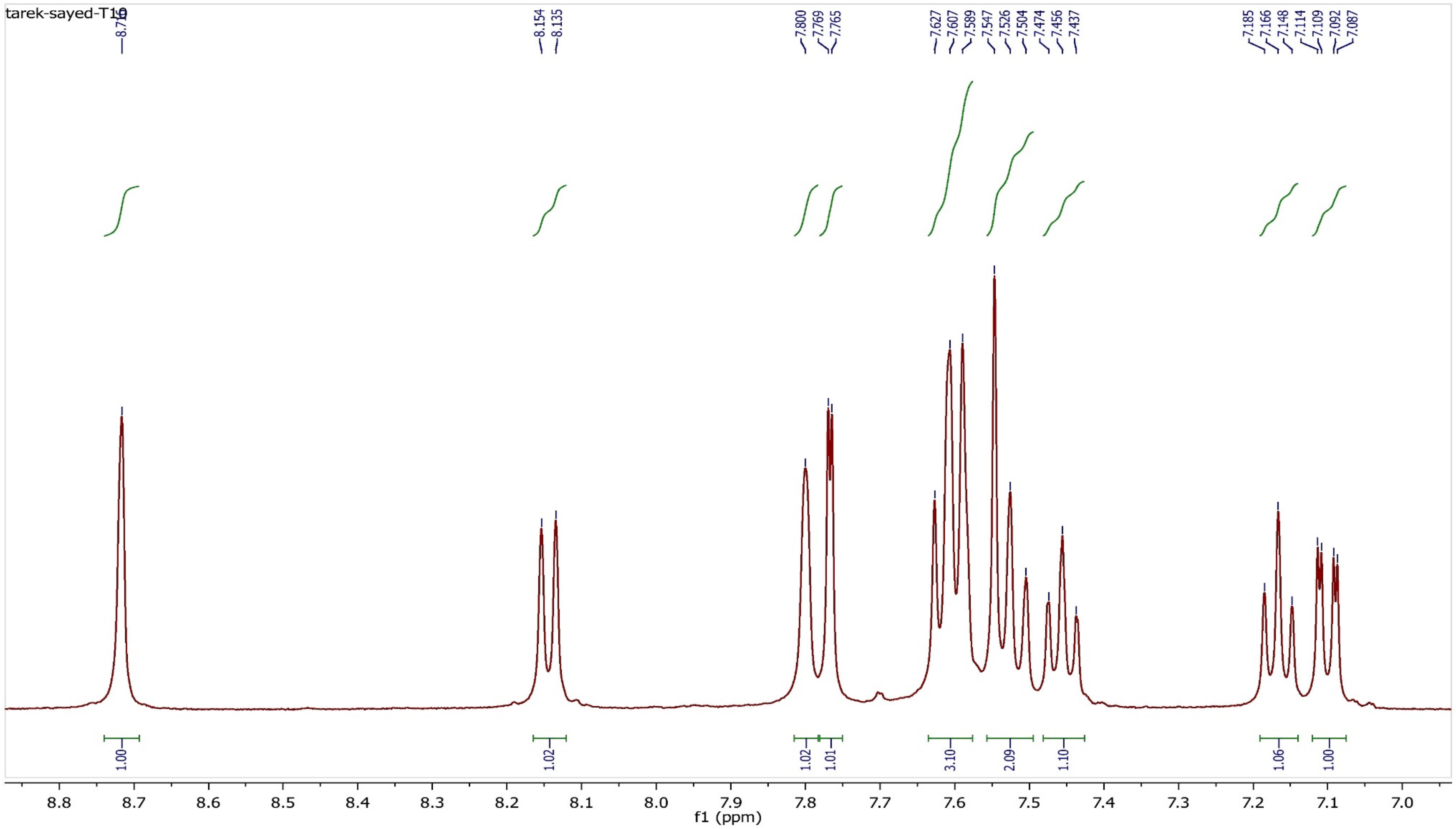

Figure S11: The enlargement of downfield region in ^{1}H-NMR spectrum of compound **4b**.

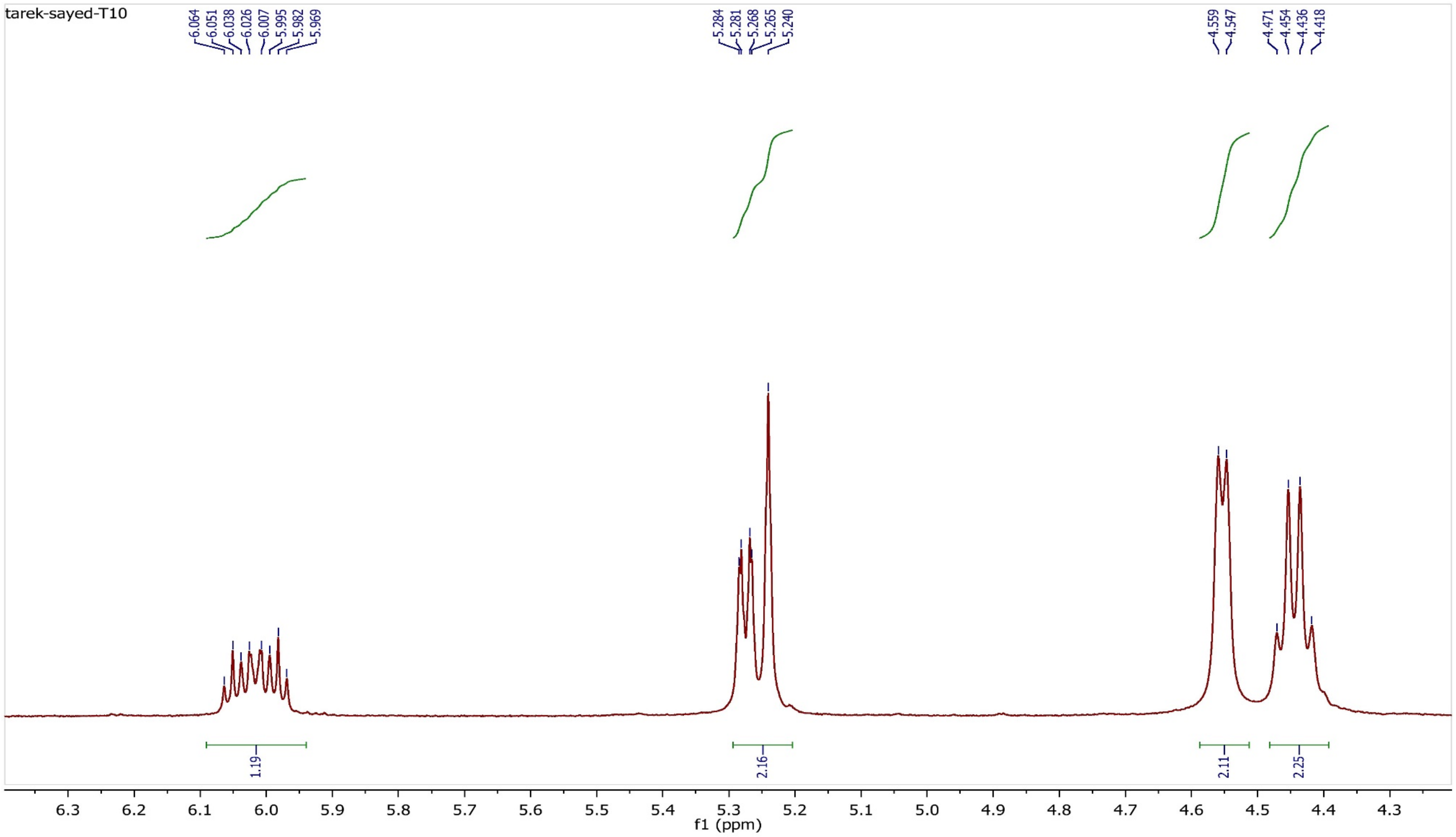

Figure S12: The enlargement of upfield region in ^{1}H-NMR spectrum of compound **4b**.

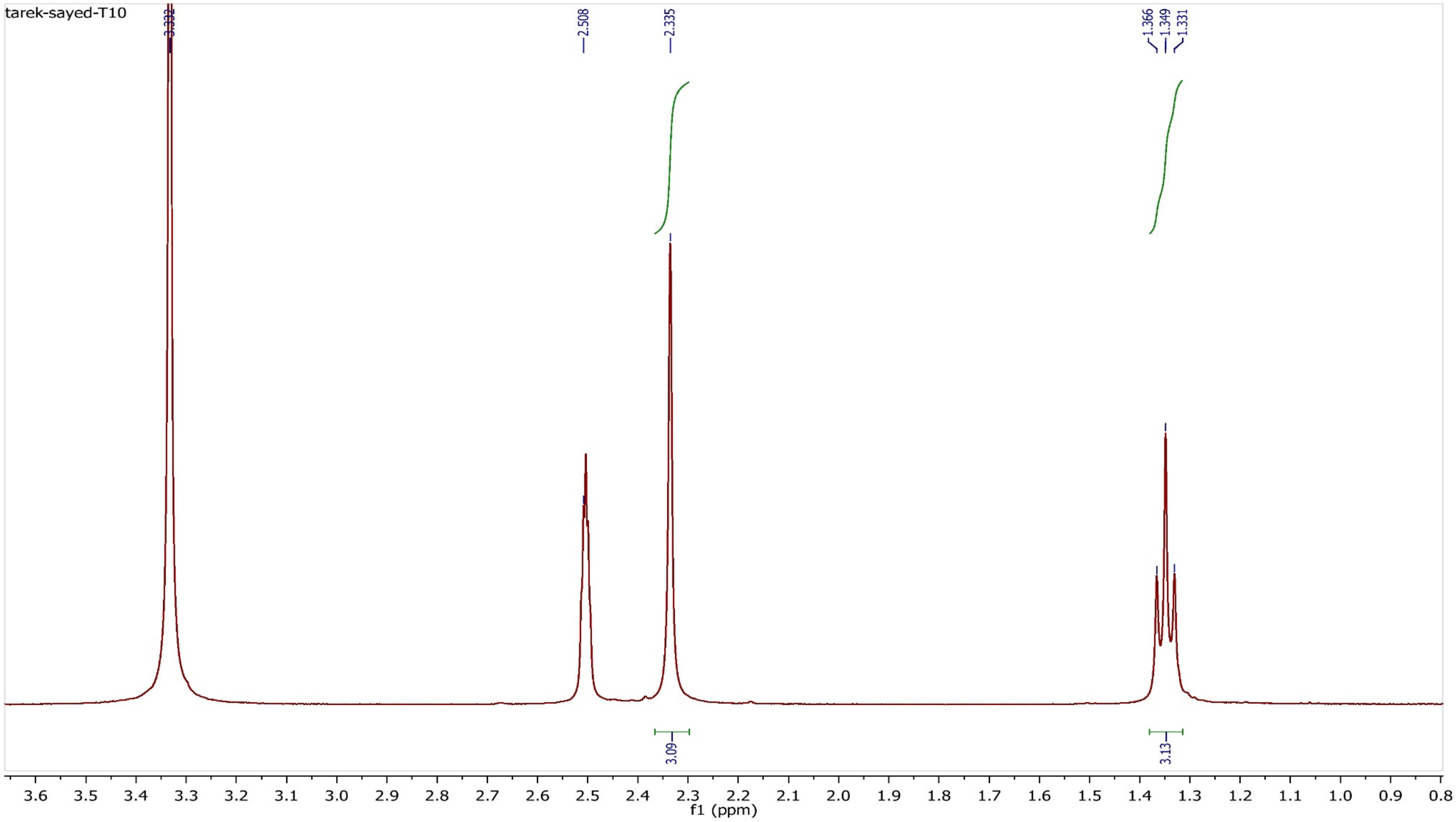

Figure S13: The enlargement of upfield region in ^{1}H-NMR spectrum of compound **4b**.

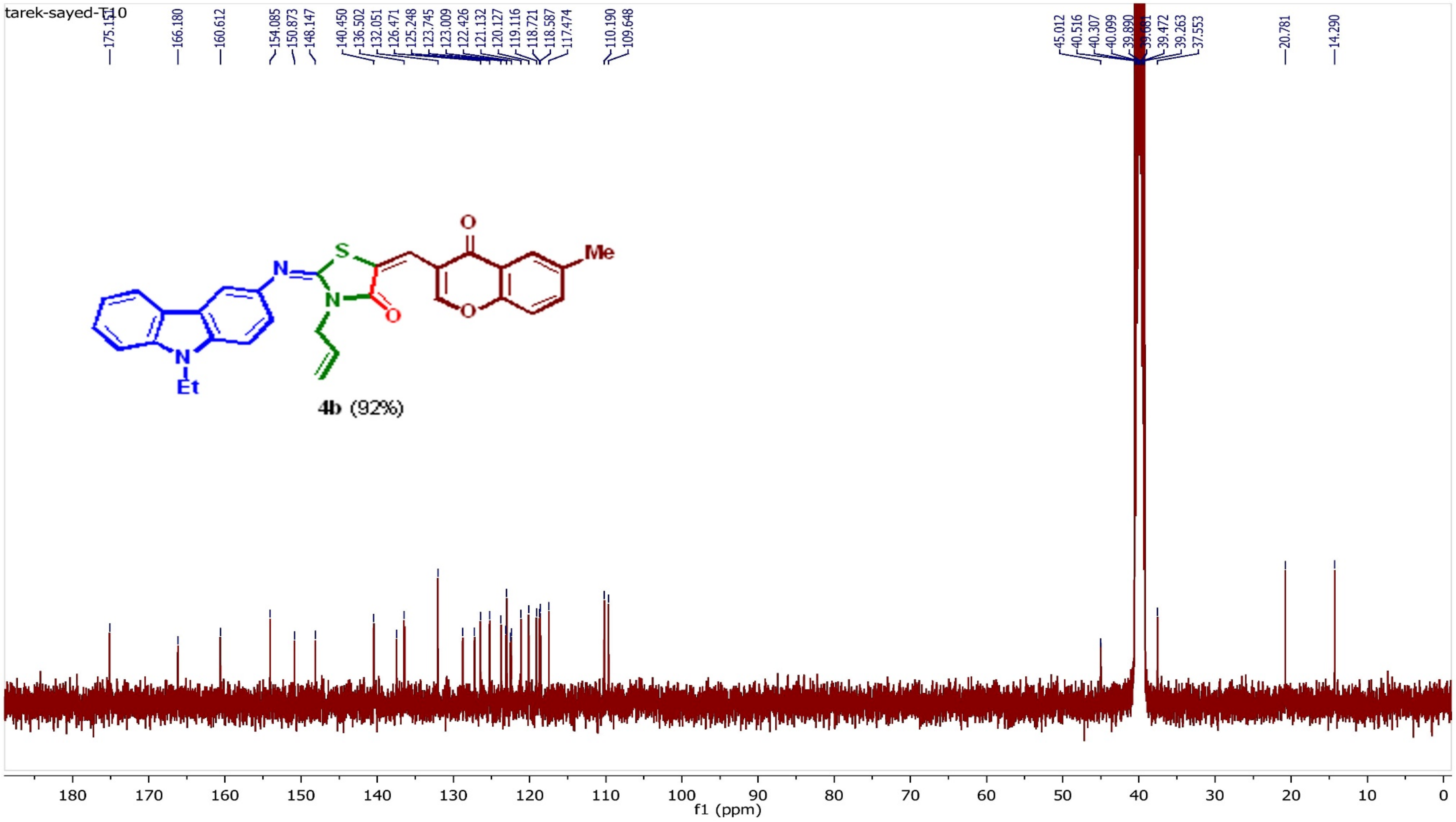

Figure S14: The ^{13}C-NMR spectrum of compound **4b**.

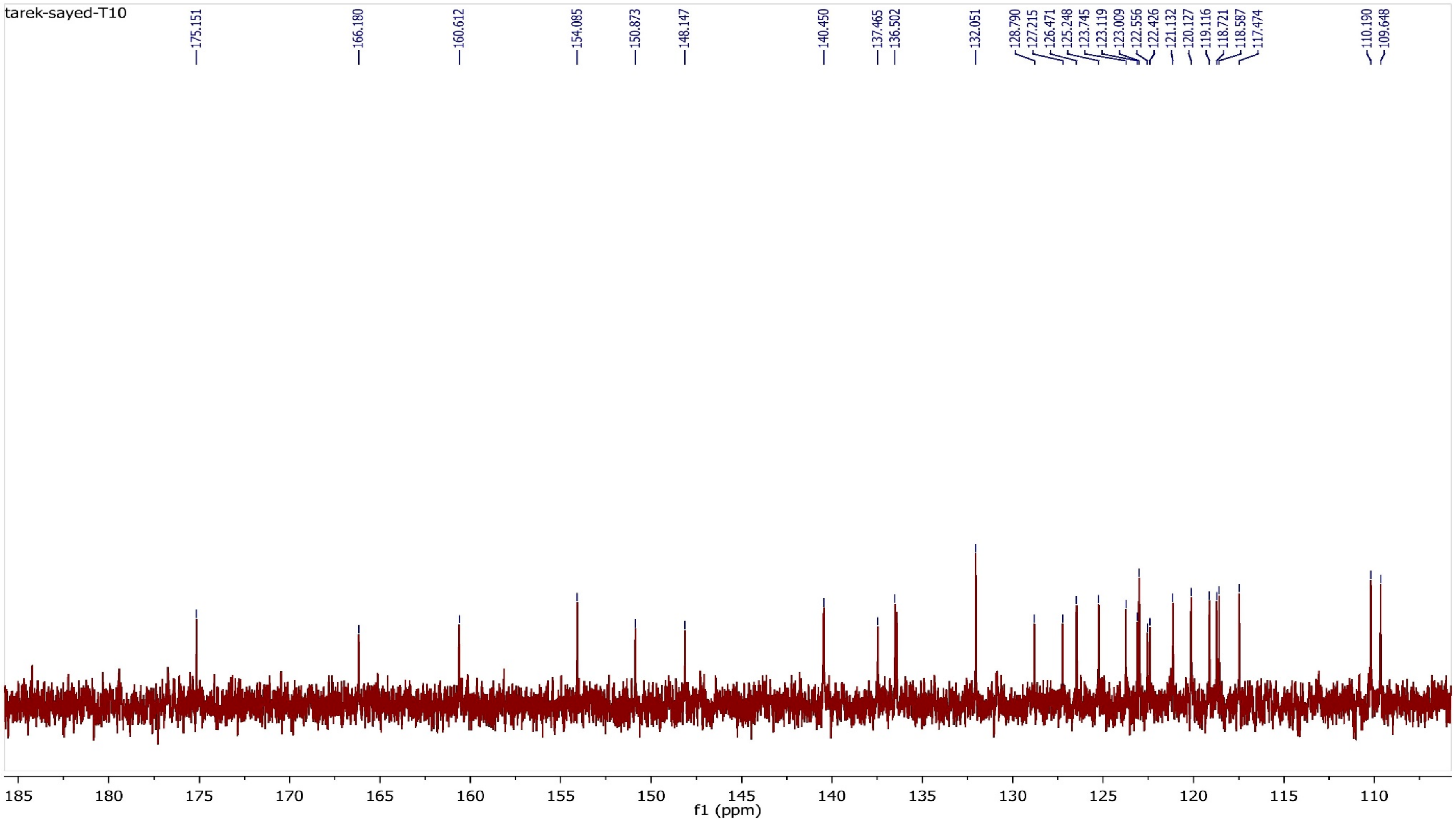

Figure S15: The enlargement of downfield region in ^{13}C-NMR spectrum of compound **4b**.

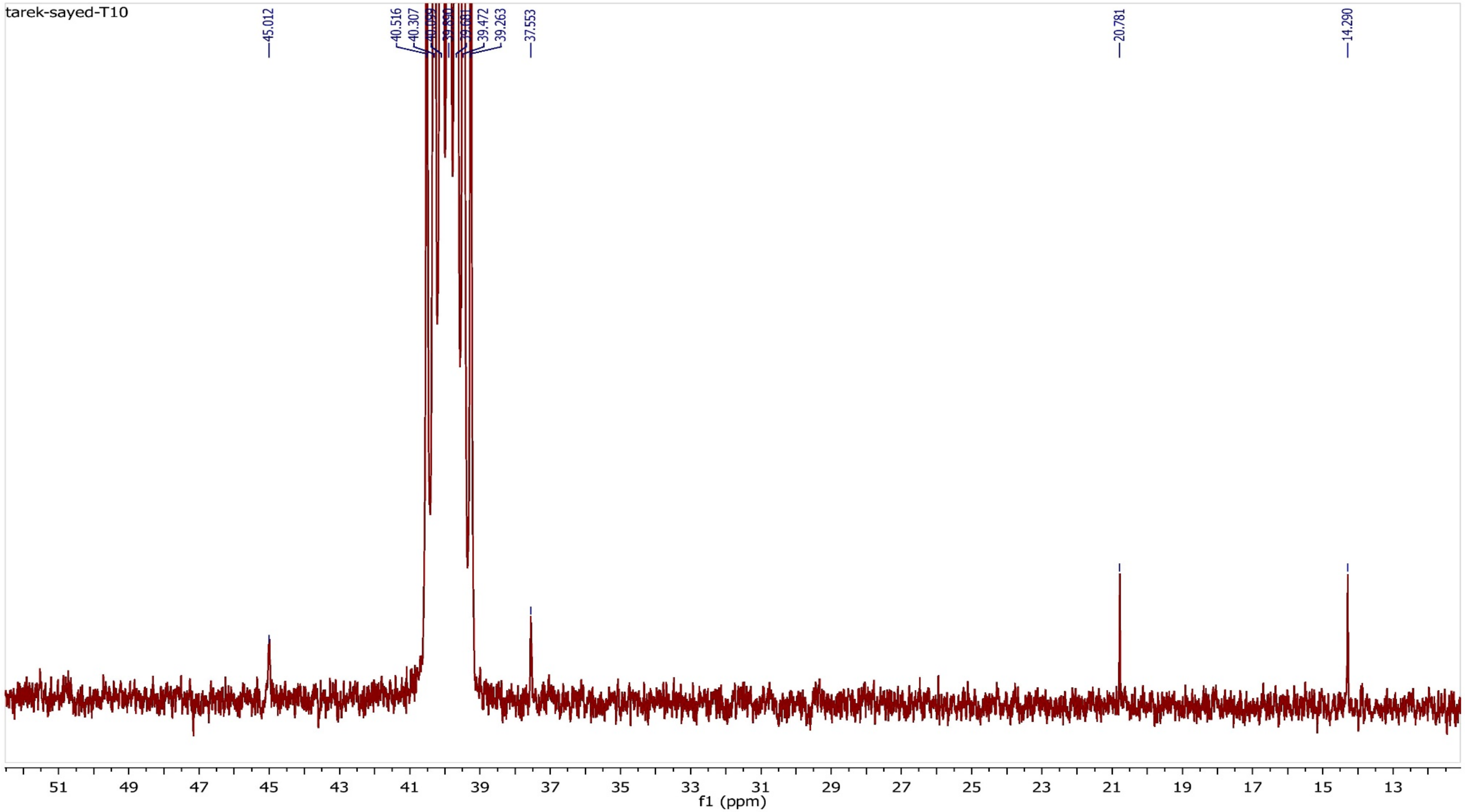

Figure S16: The enlargement of upfield region in ^{13}C-NMR spectrum of compound **4b**.

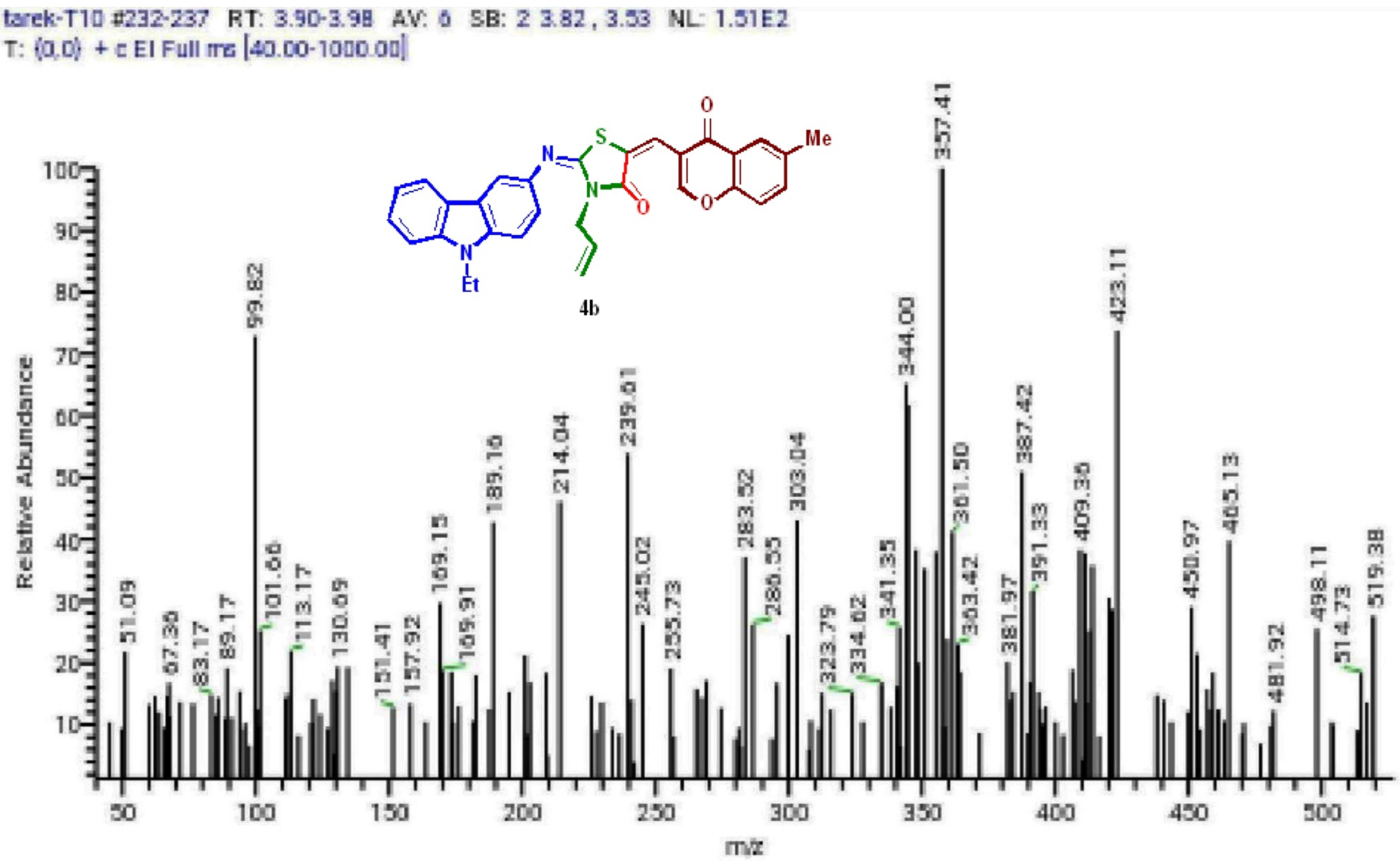

Figure S17: The mass spectrum of compound **4b**.

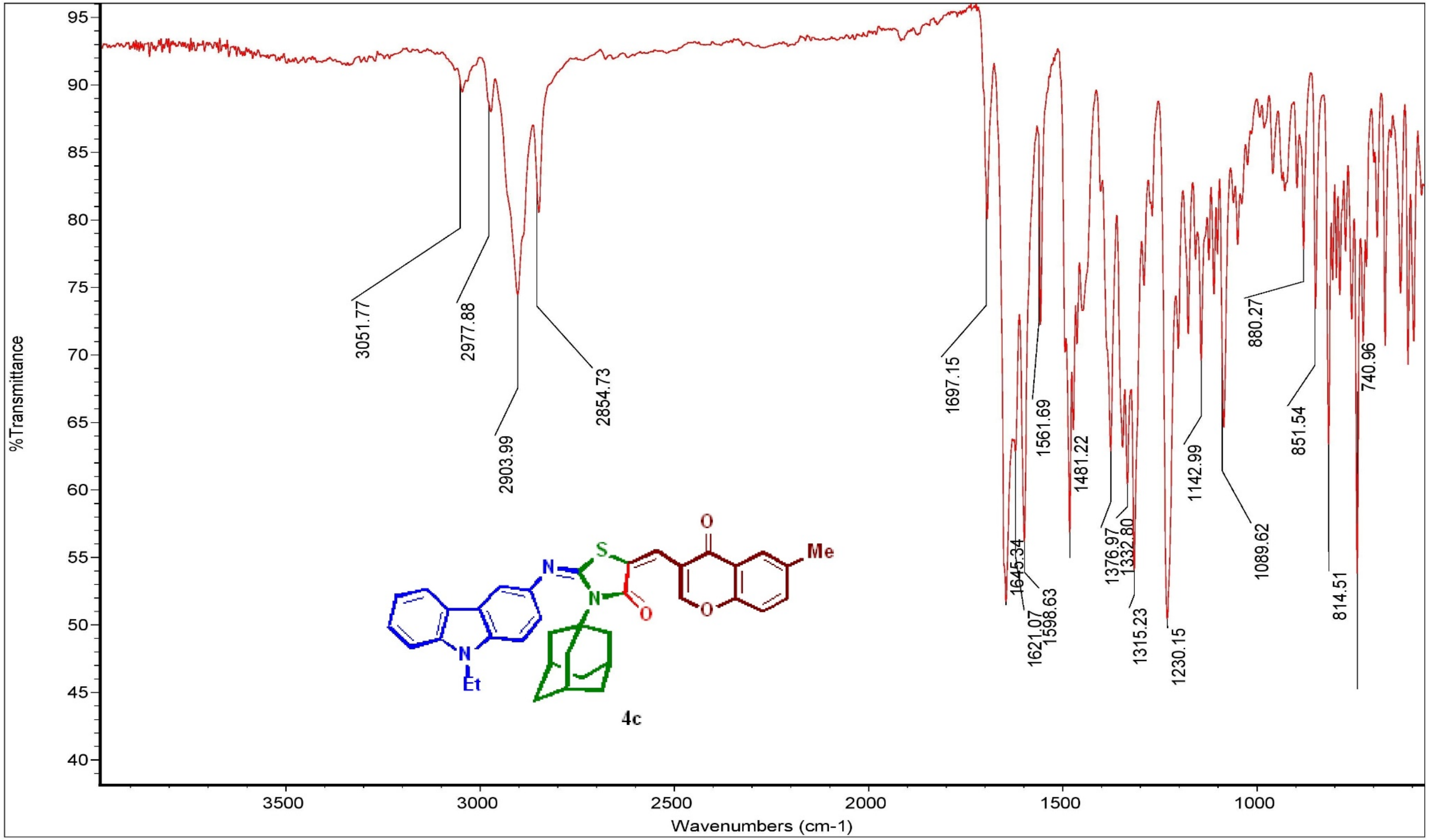

Figure S18: The IR spectrum of compound **4c**.

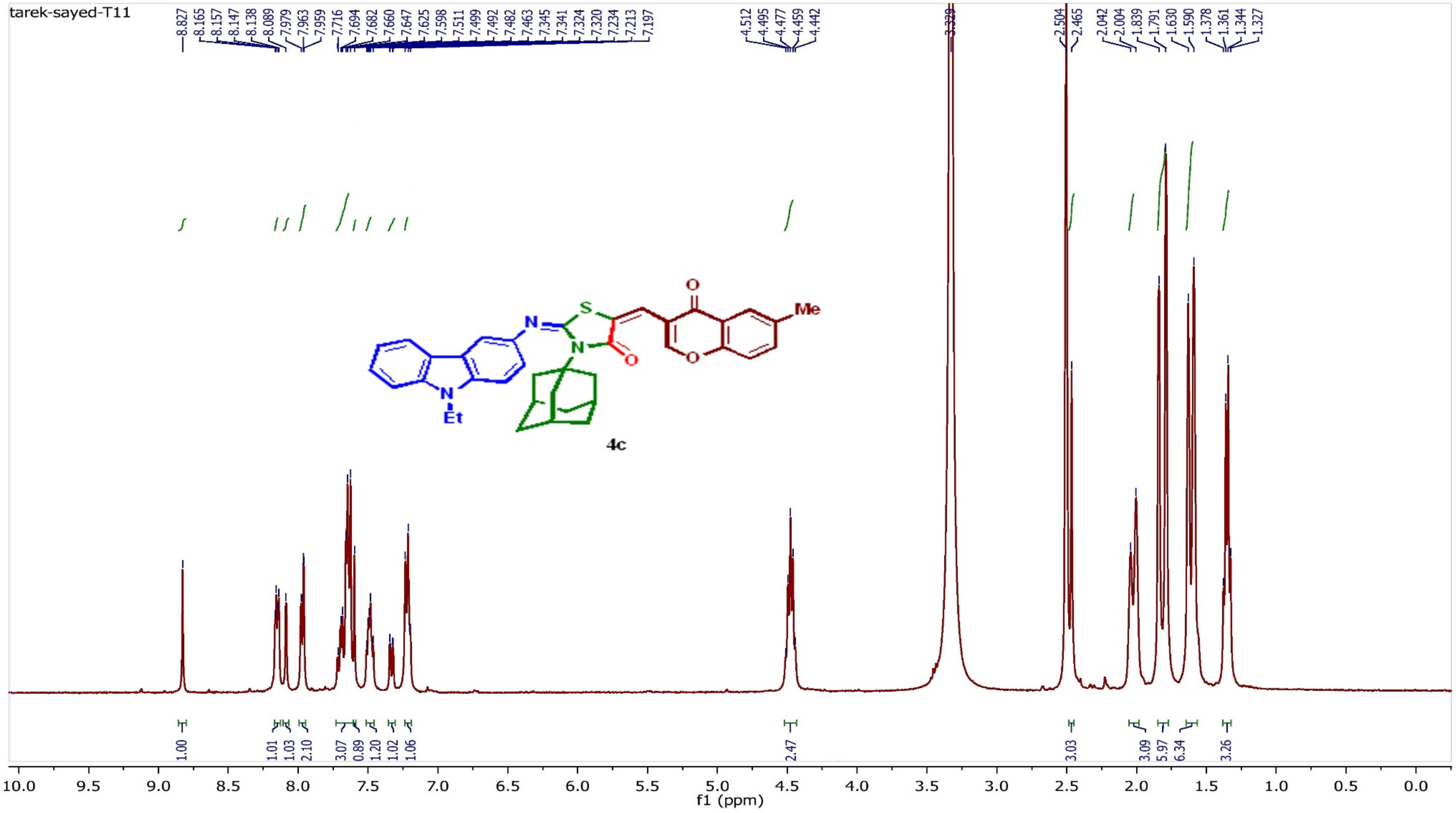

Figure S19: The ^{1}H-NMR spectrum of compound **4c**.

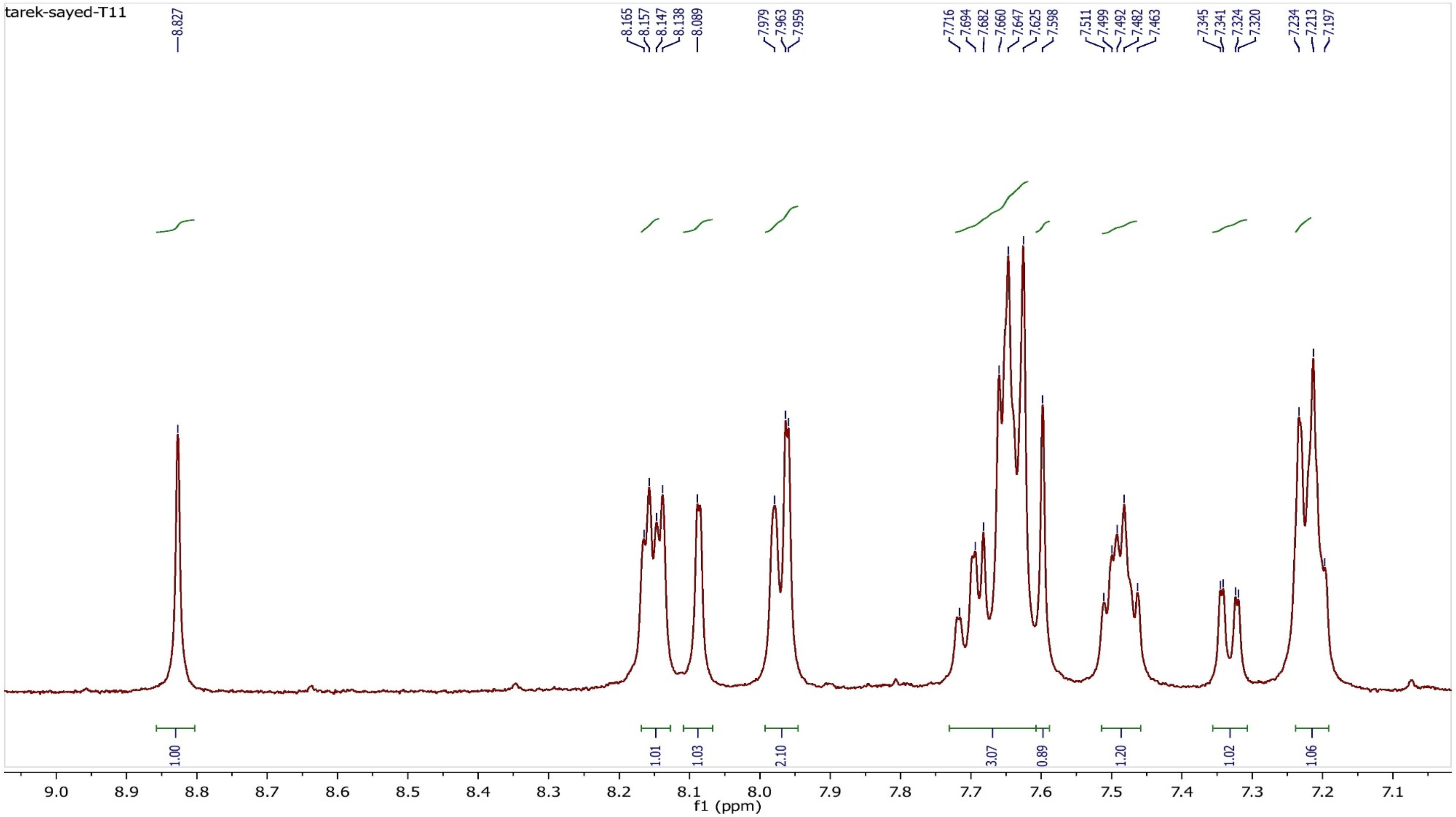

Figure S20: The enlargement of downfield region in ^{1}H-NMR spectrum of compound **4c**.

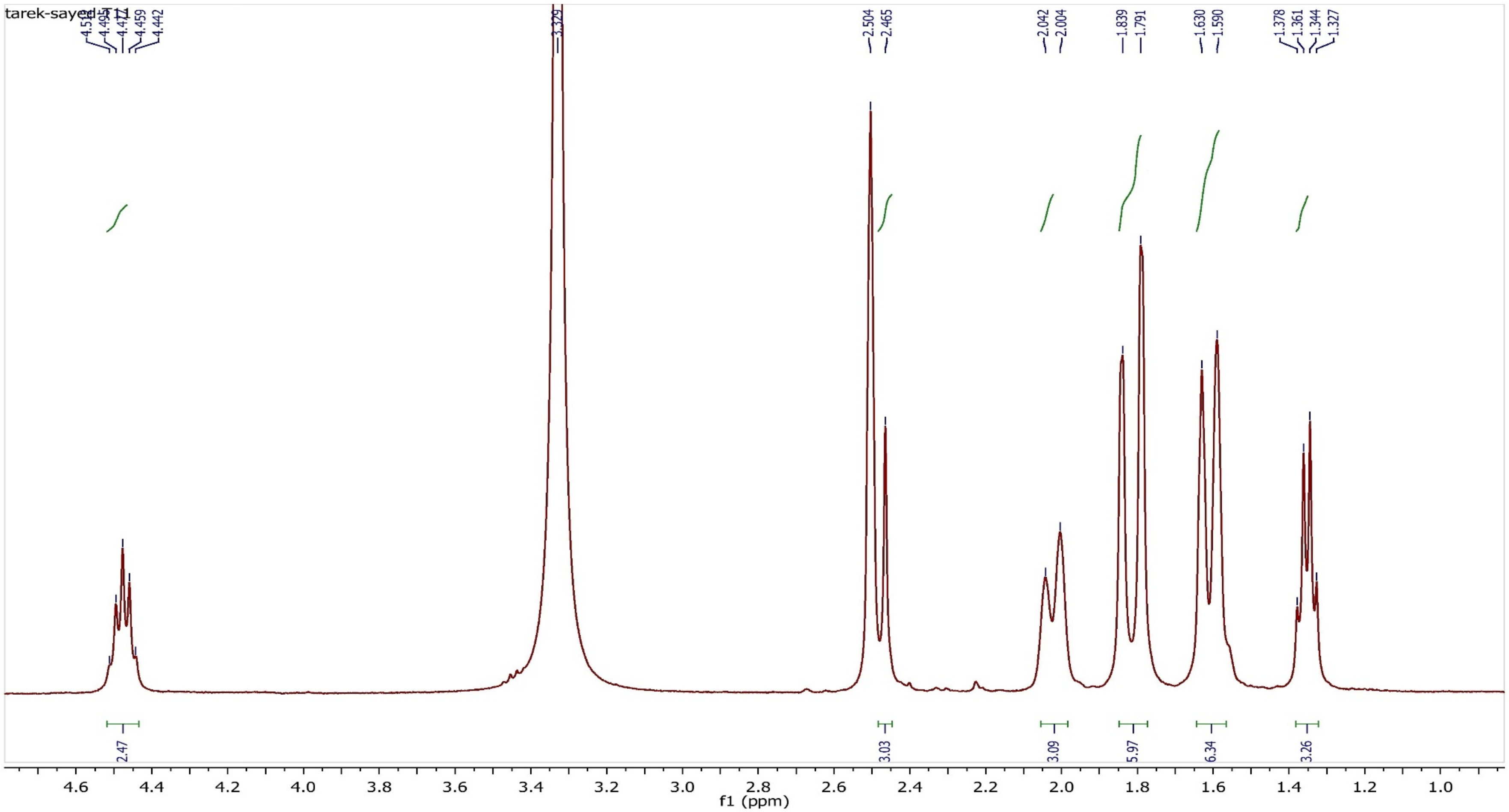

Figure S21: The enlargement of upfield region in ^{1}H-NMR spectrum of compound **4c**.

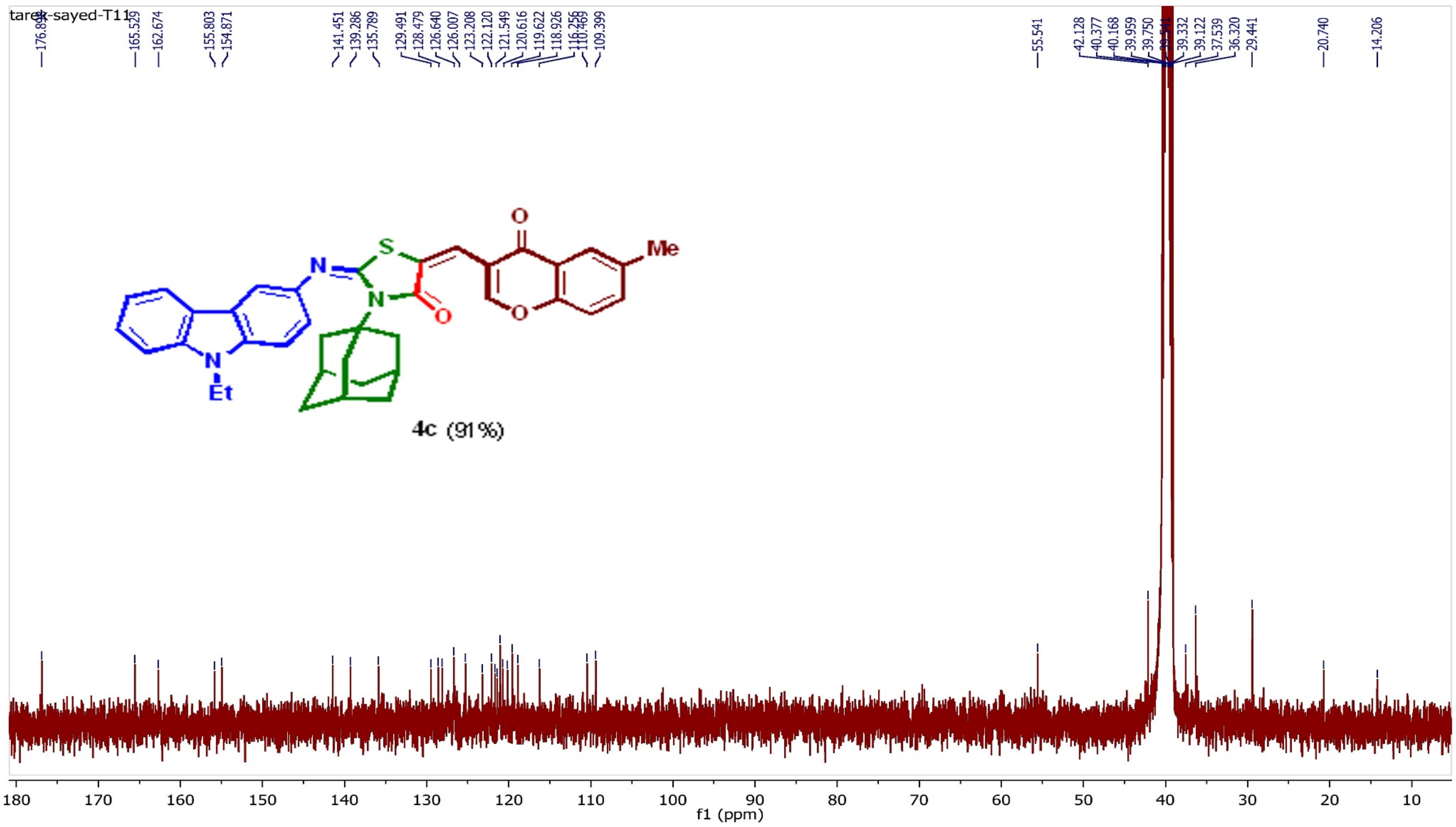

Figure S22: The ^{13}C-NMR spectrum of compound **4c**.

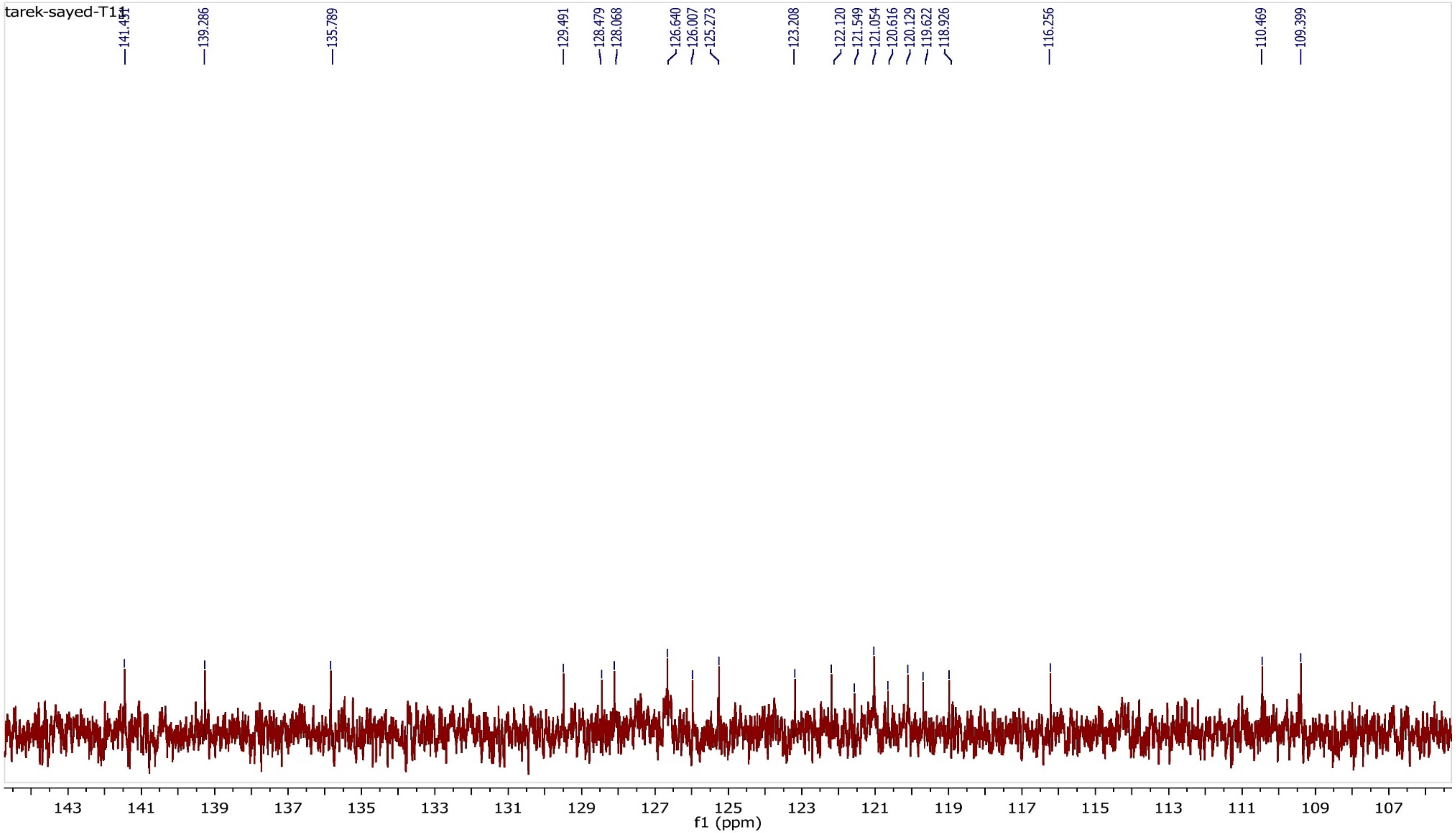

Figure S23: The enlargement of downfield region in ^{13}C-NMR spectrum of compound **4c**.

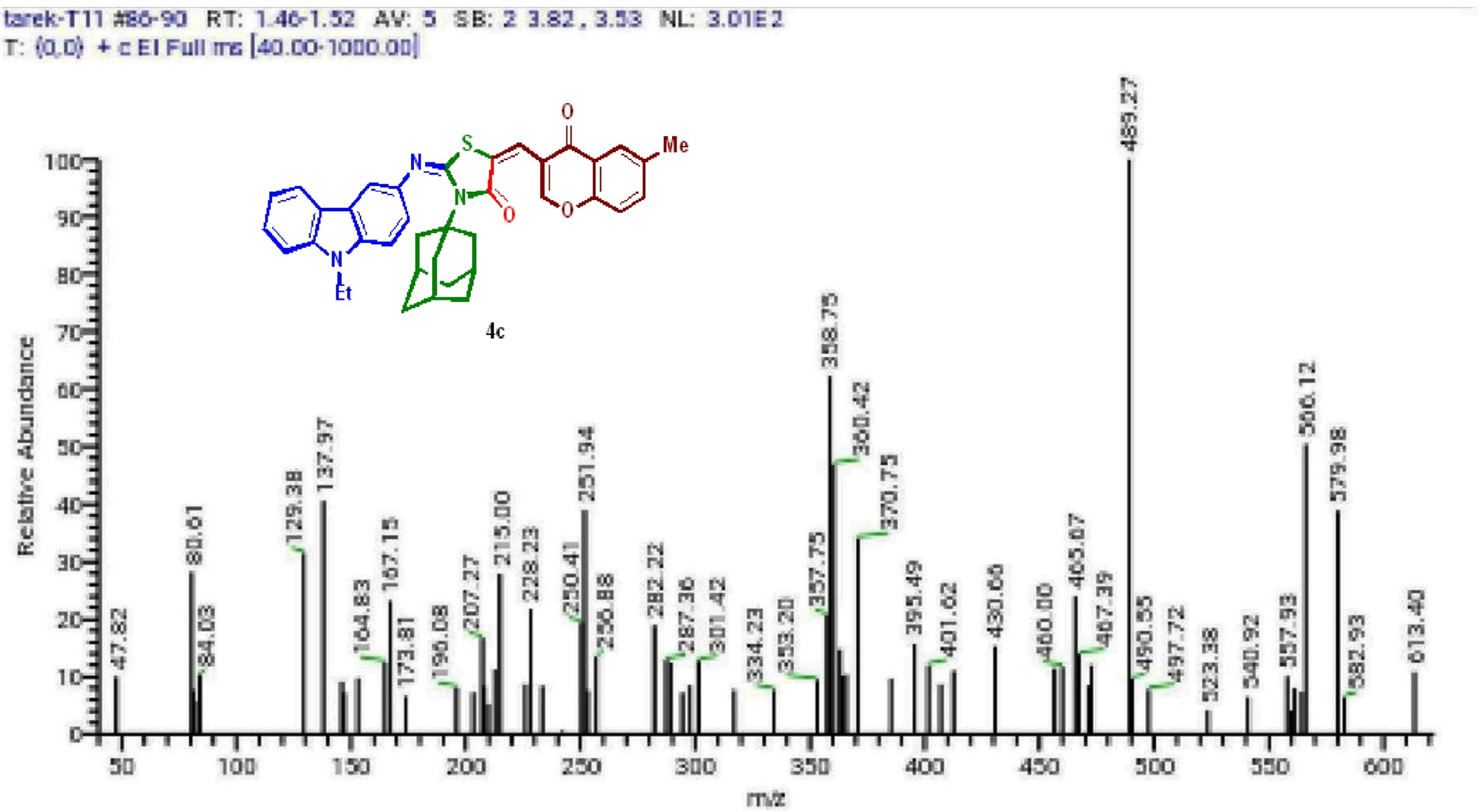

Figure S24: The mass spectrum of compound **4c**.

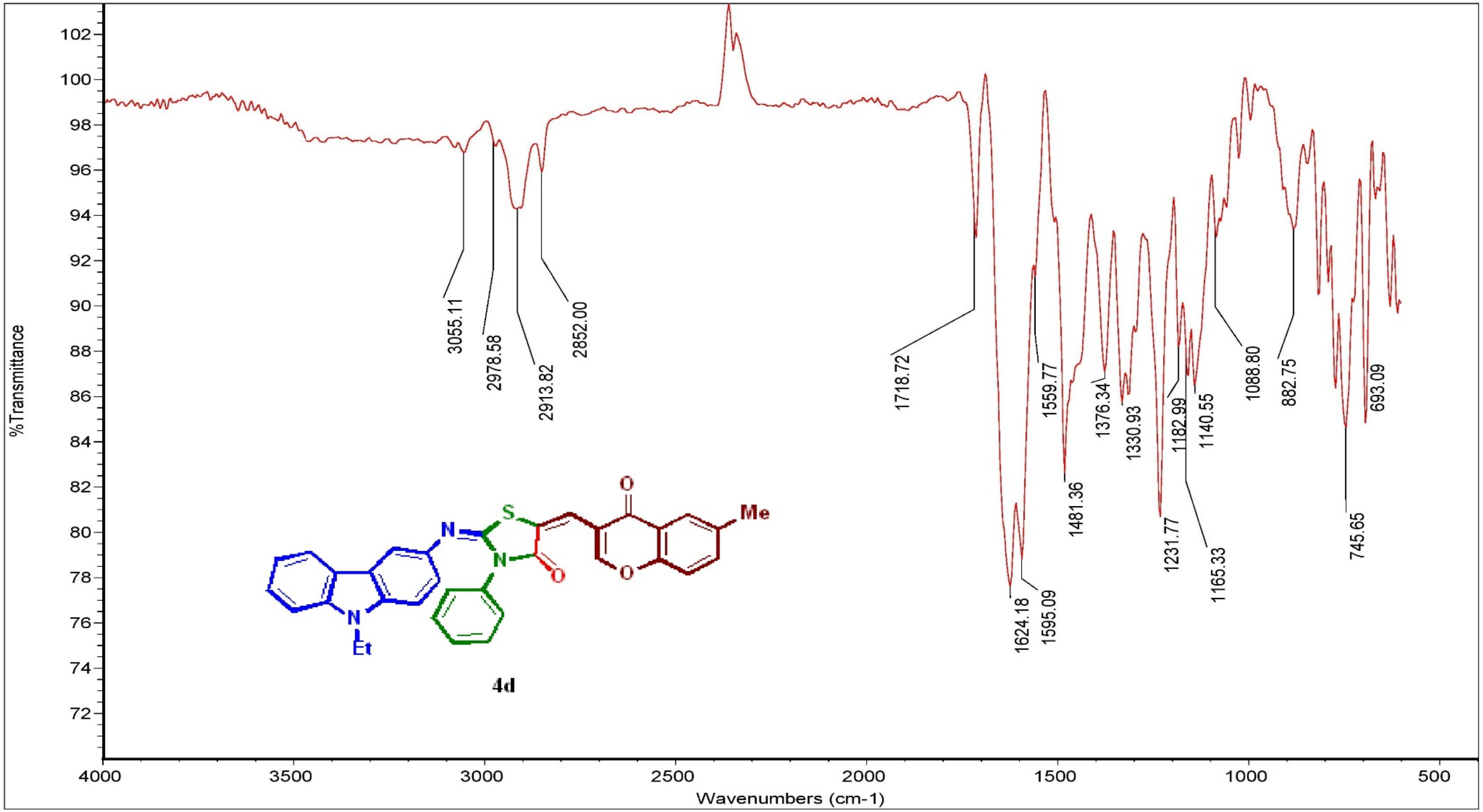

Figure S25: The IR spectrum of compound **4d**.

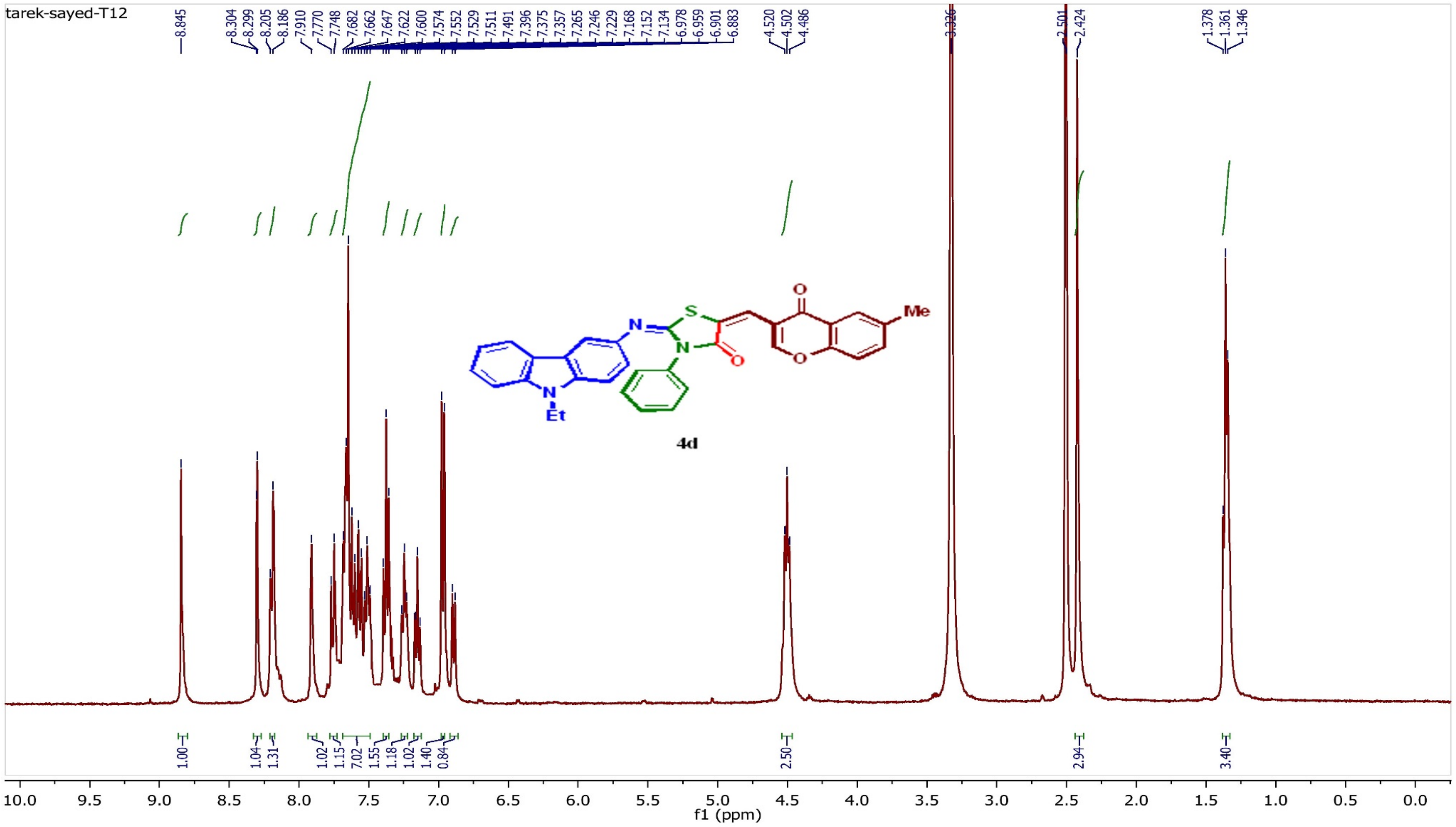

Figure S26: The ^{1}H-NMR spectrum of compound **4d**.

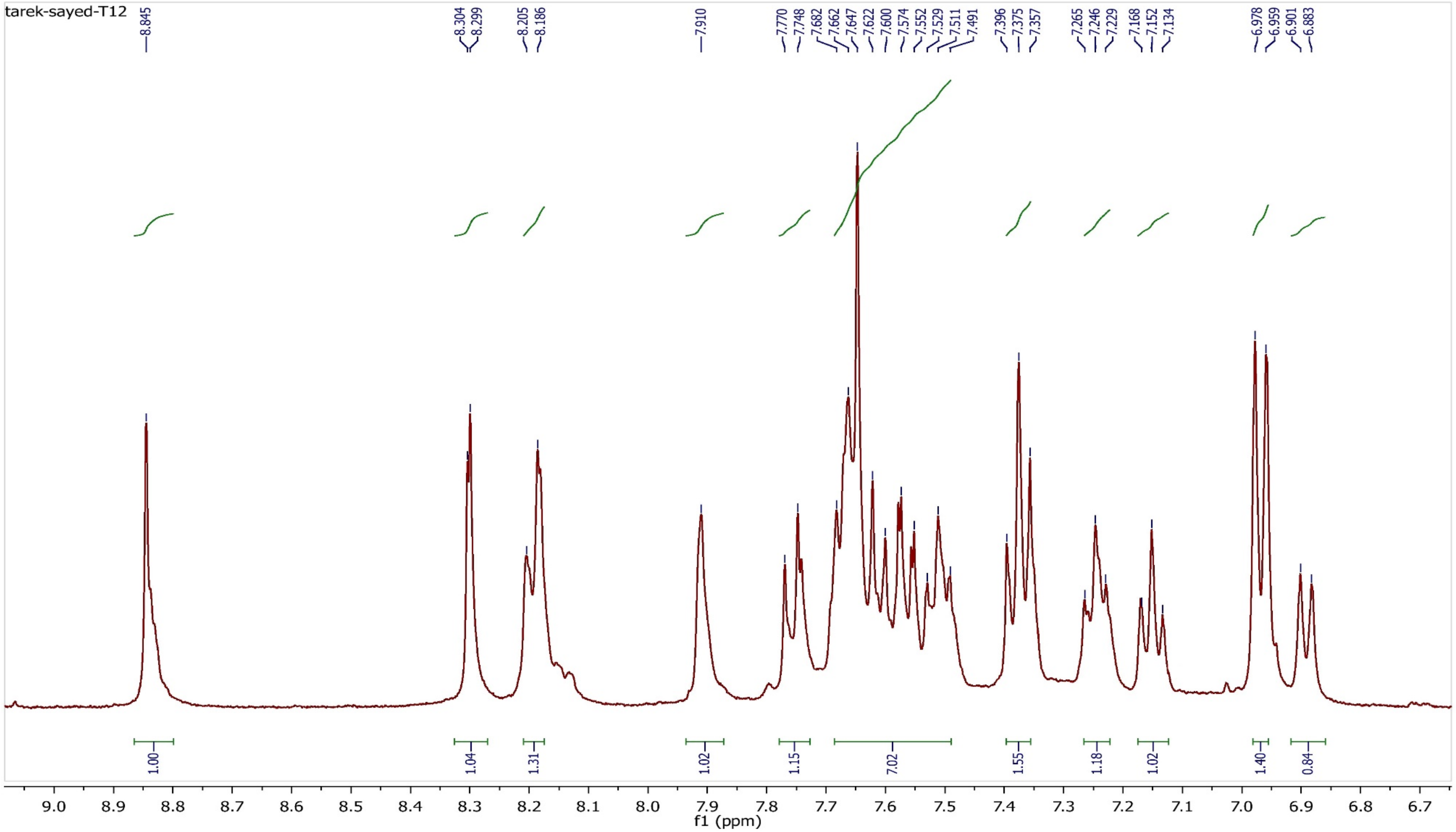

Figure S27: The enlargement of downfield region in ^{1}H-NMR spectrum of compound **4d**.

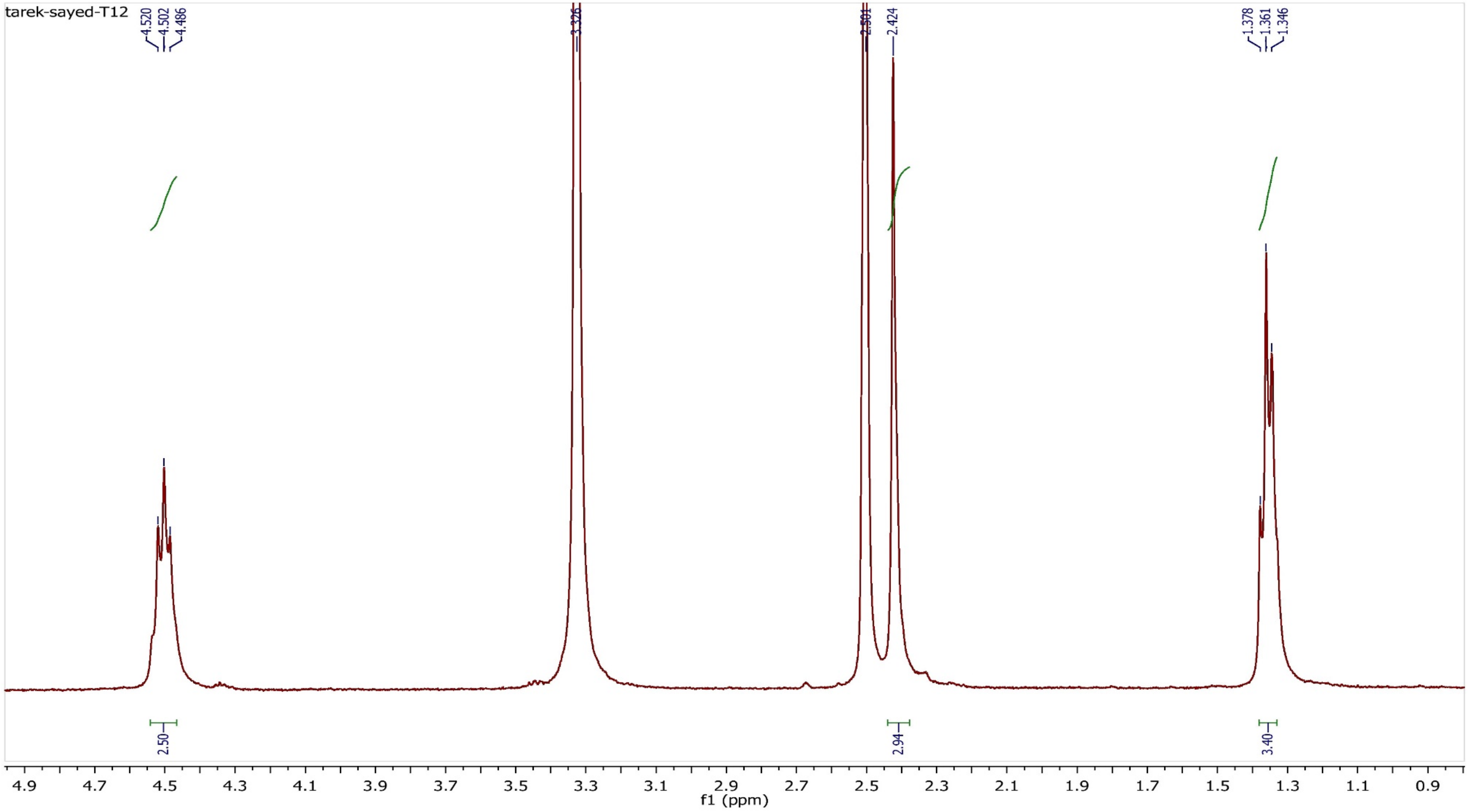

Figure S28: The enlargement of upfield region in ^{1}H-NMR spectrum of compound **4d**.

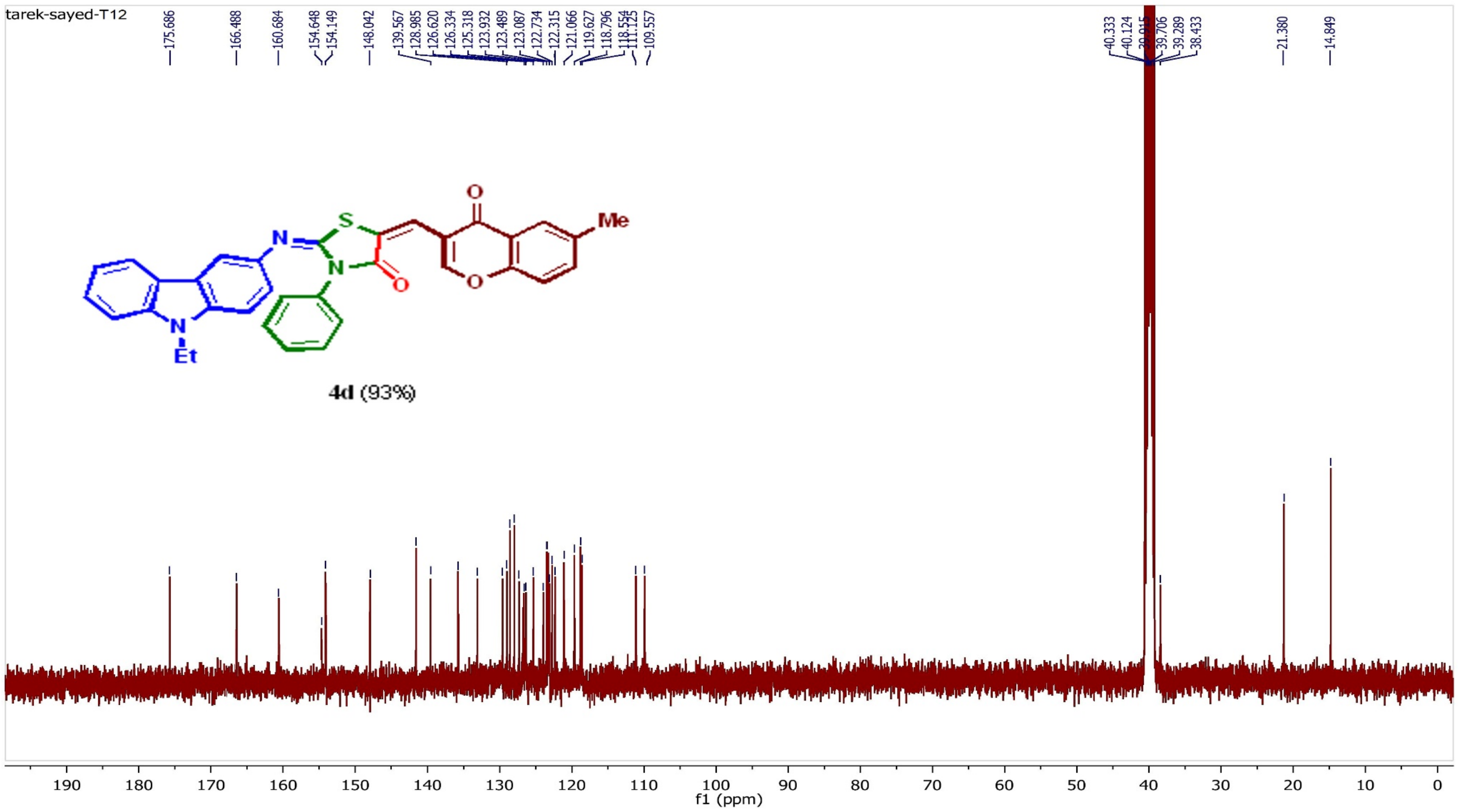

Figure S29: The ^{13}C-NMR spectrum of compound **4d**.

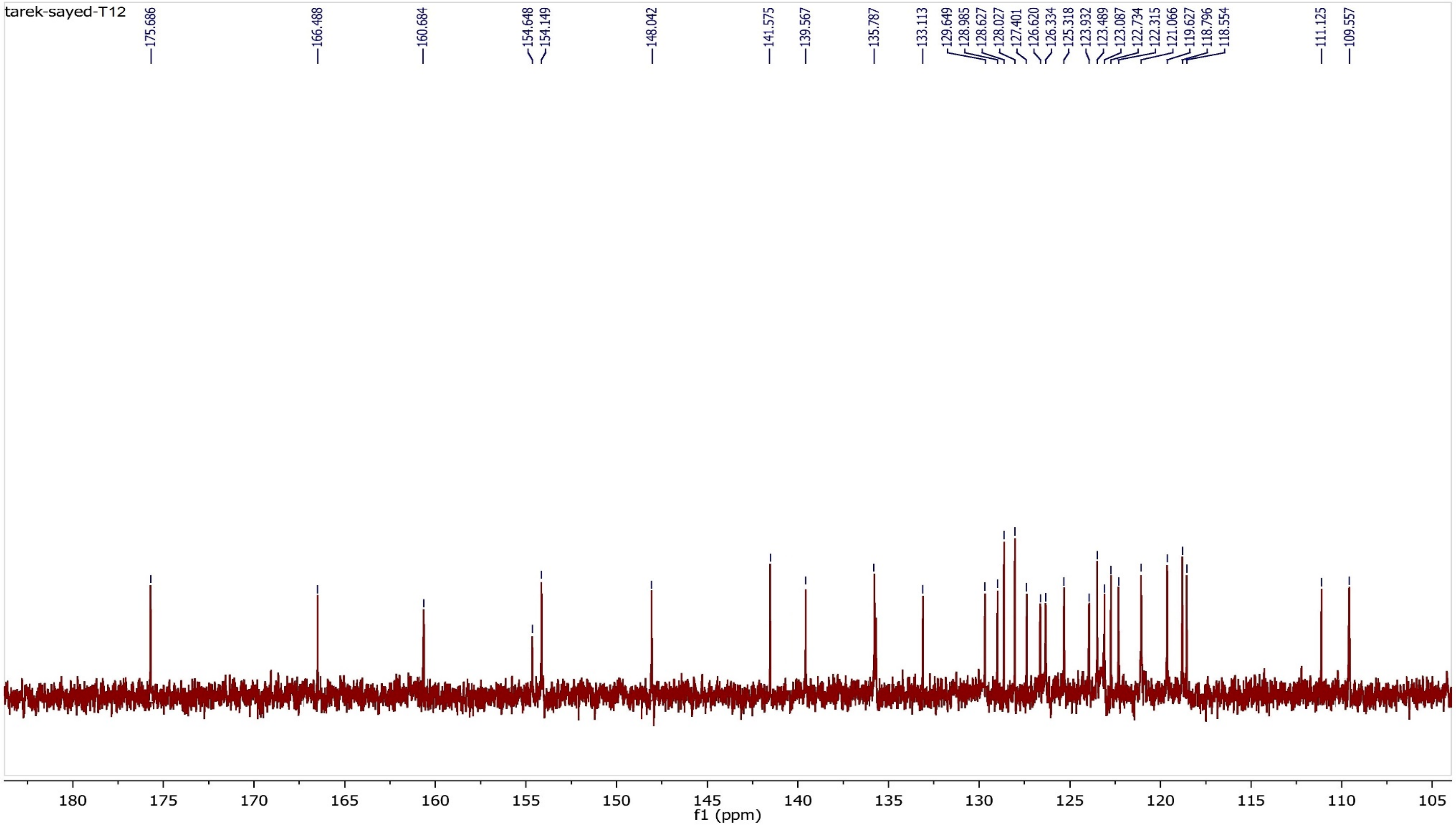

Figure S30: The enlargement of downfield region in ^{13}C-NMR spectrum of compound **4d**.

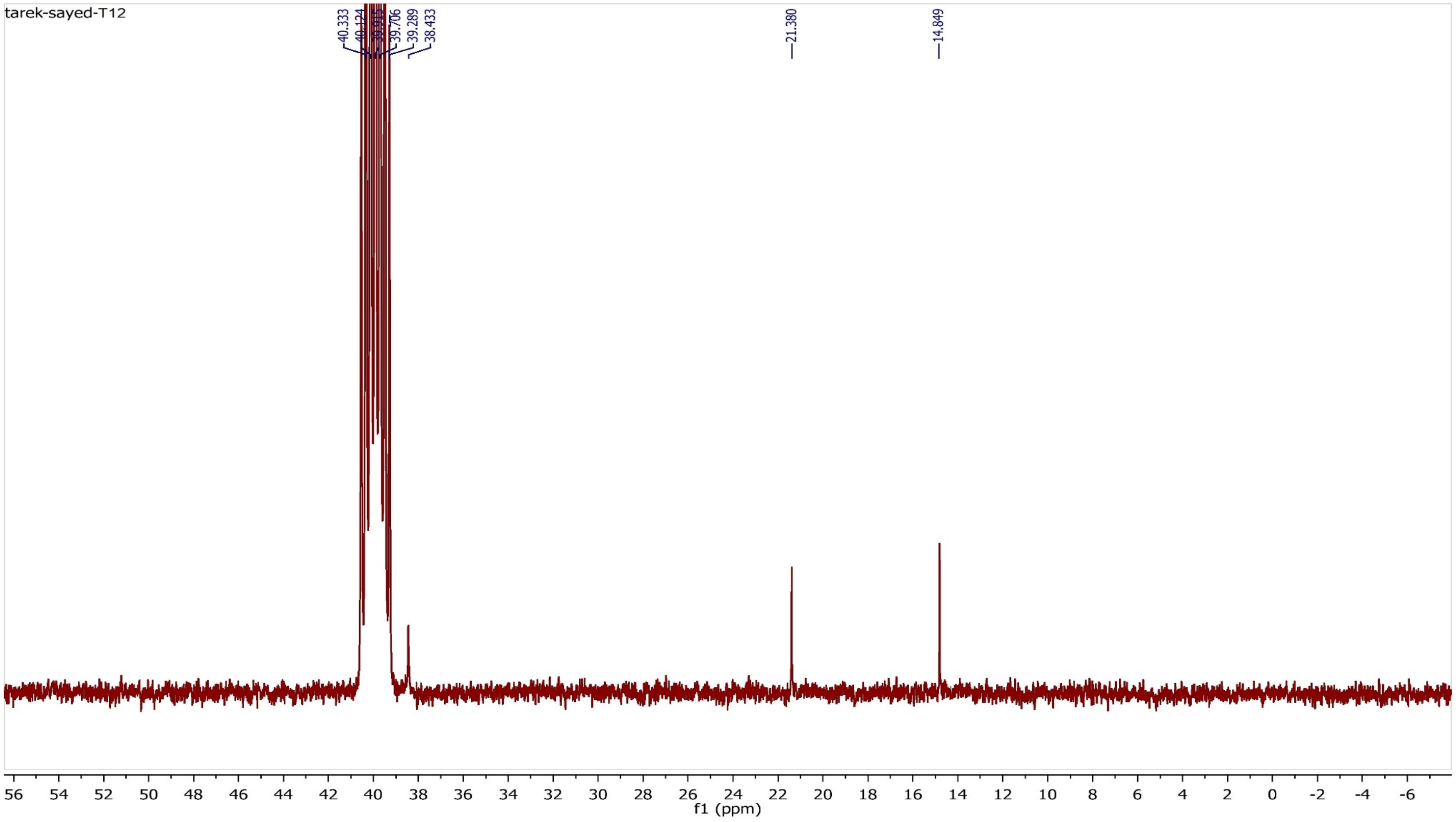

Figure S31: The enlargement of upfield region in ^{13}C-NMR spectrum of compound **4d**.

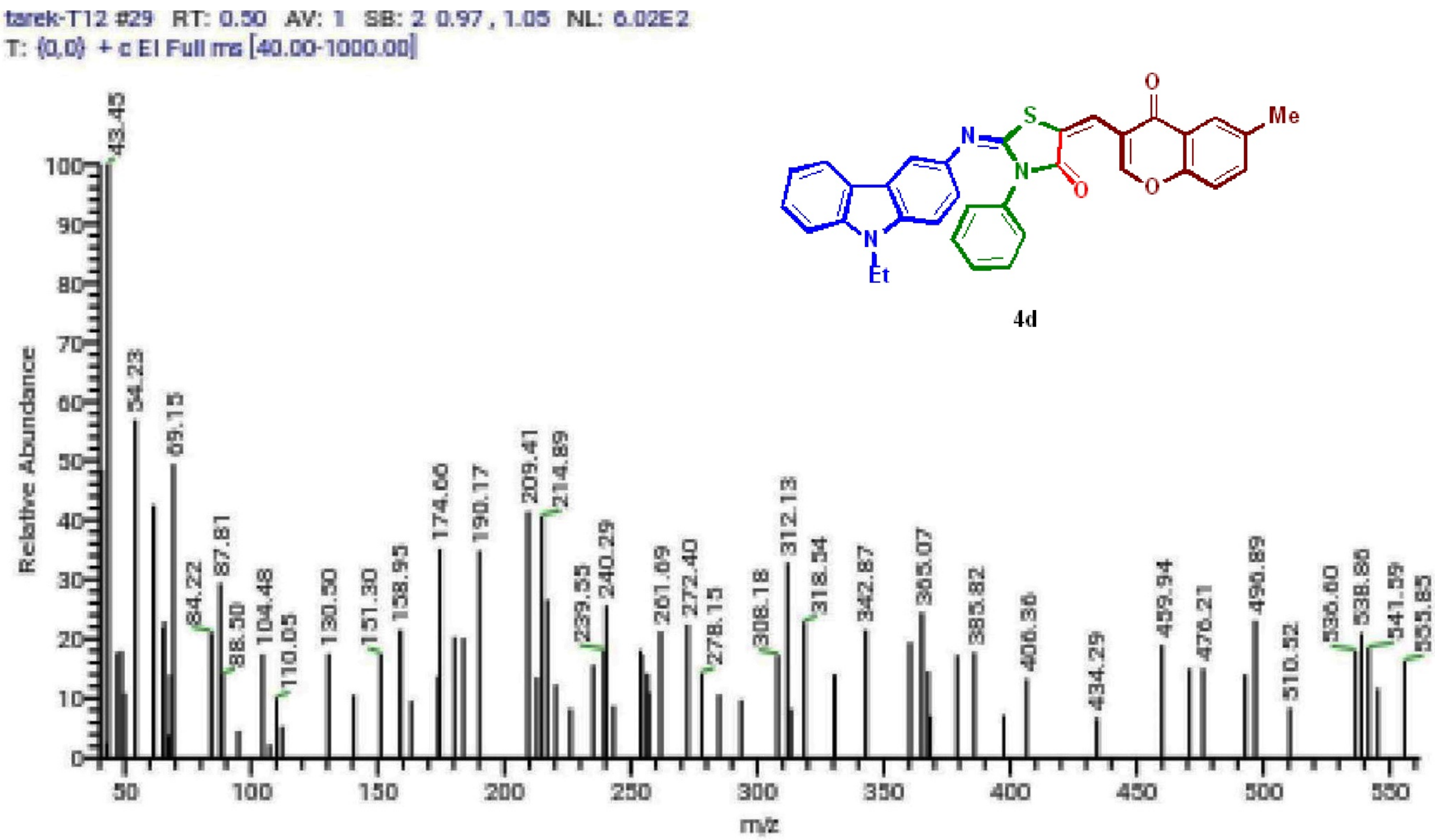

Figure S32: The mass spectrum of compound **4d**.

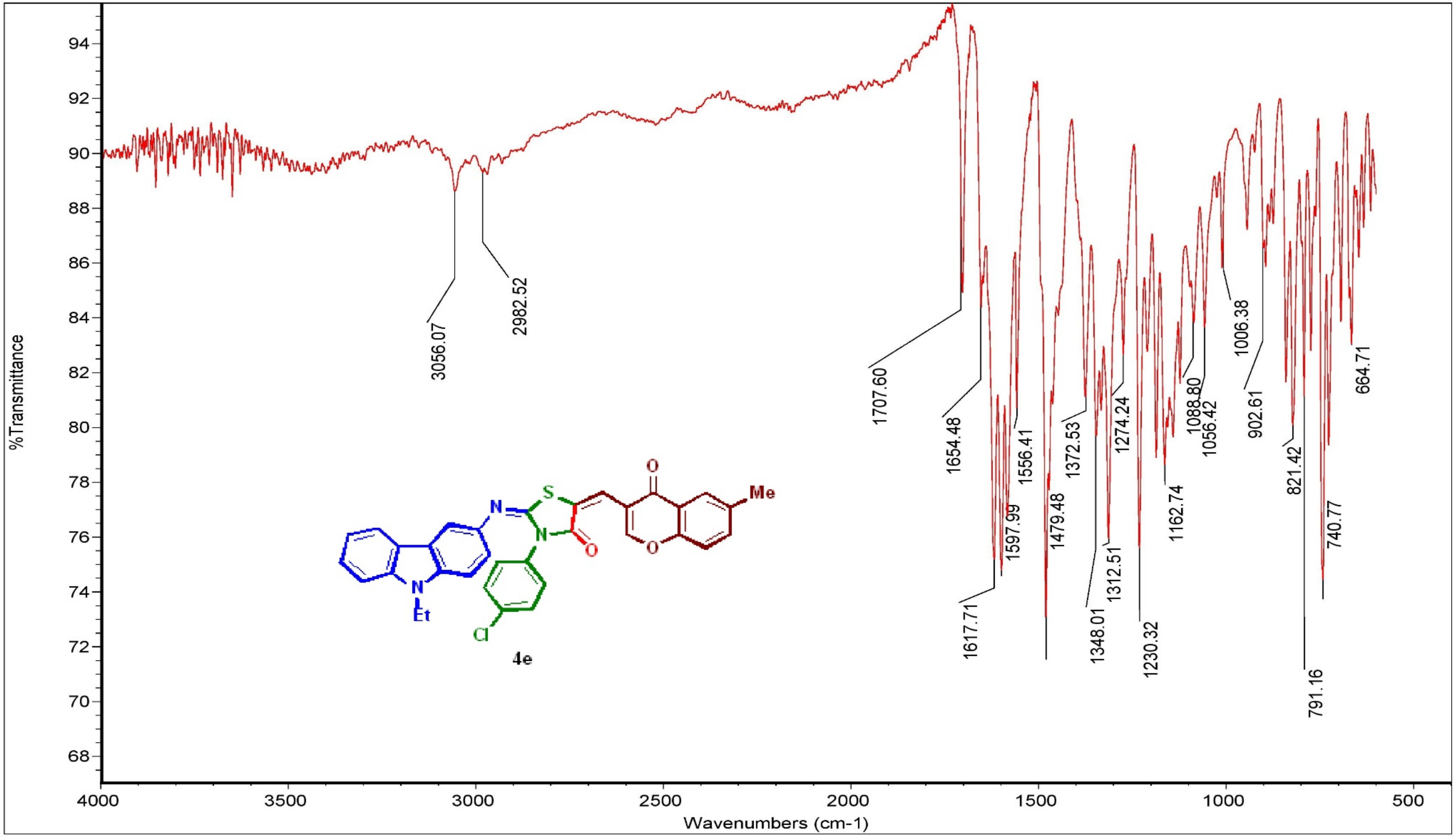

Figure S33: The IR spectrum of compound **4e**.

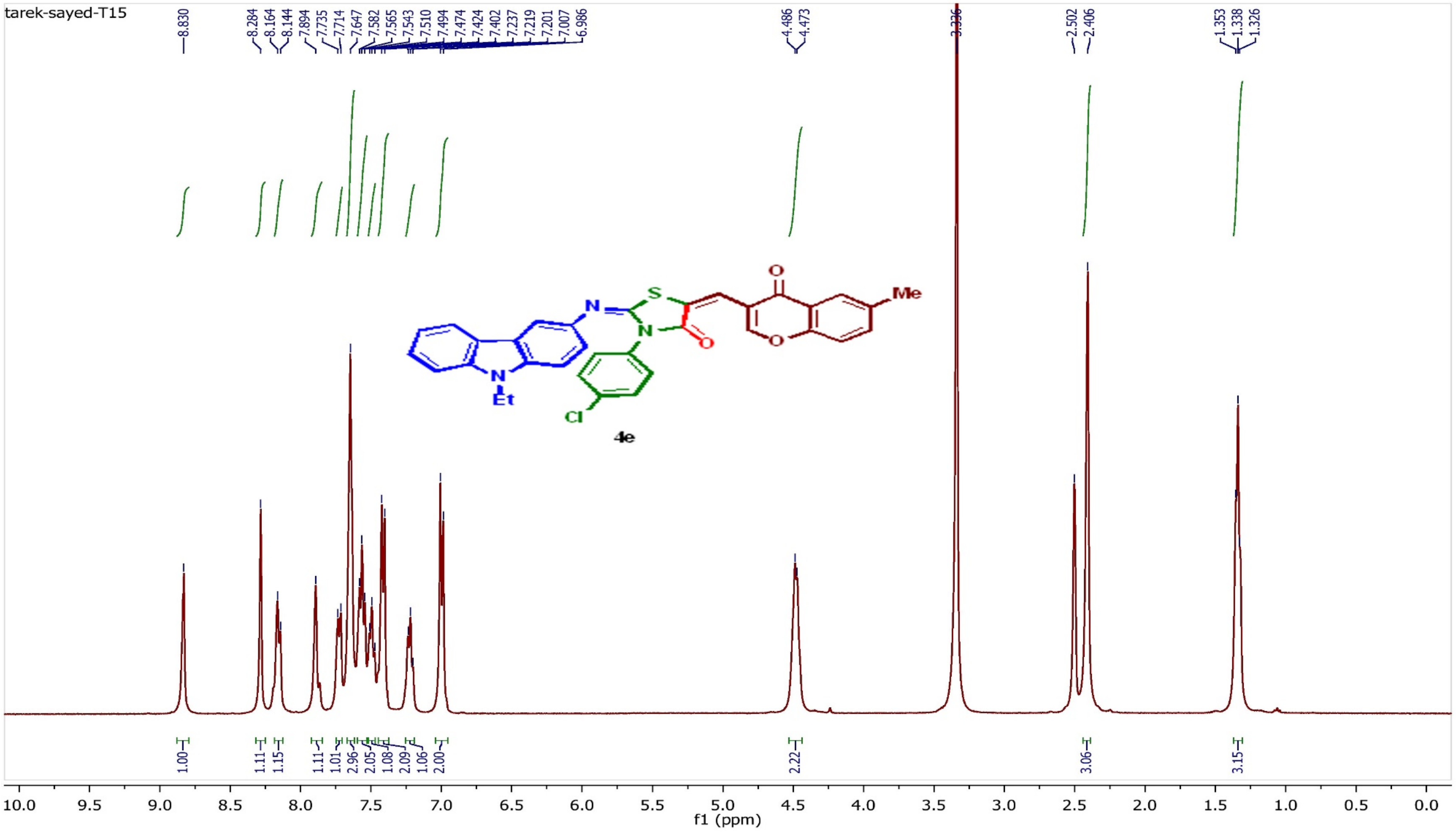

Figure S34: The ^{1}H-NMR spectrum of compound **4e**.

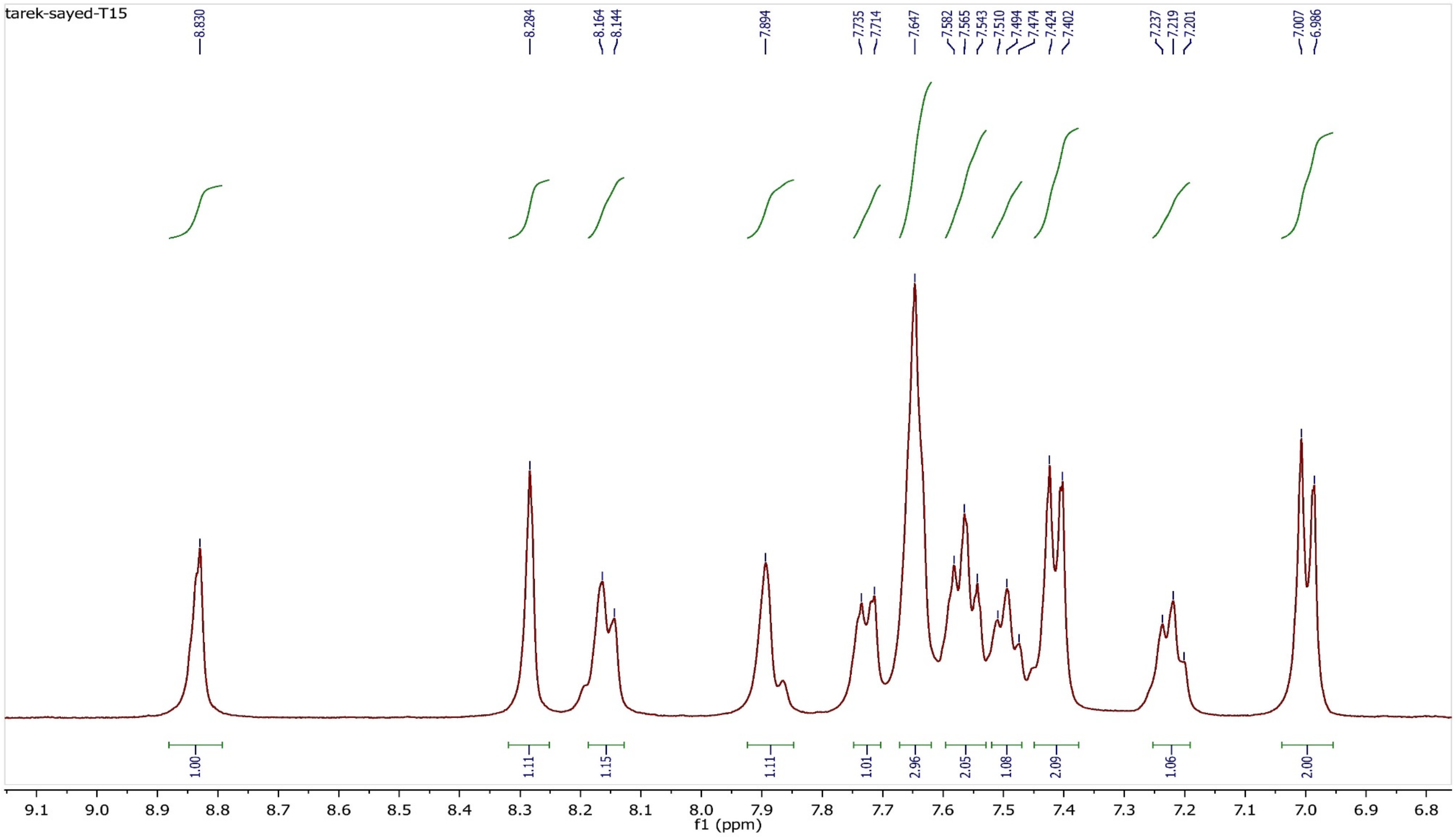

Figure S35: The enlargement of downfield region in ^{1}H-NMR spectrum of compound **4e**.

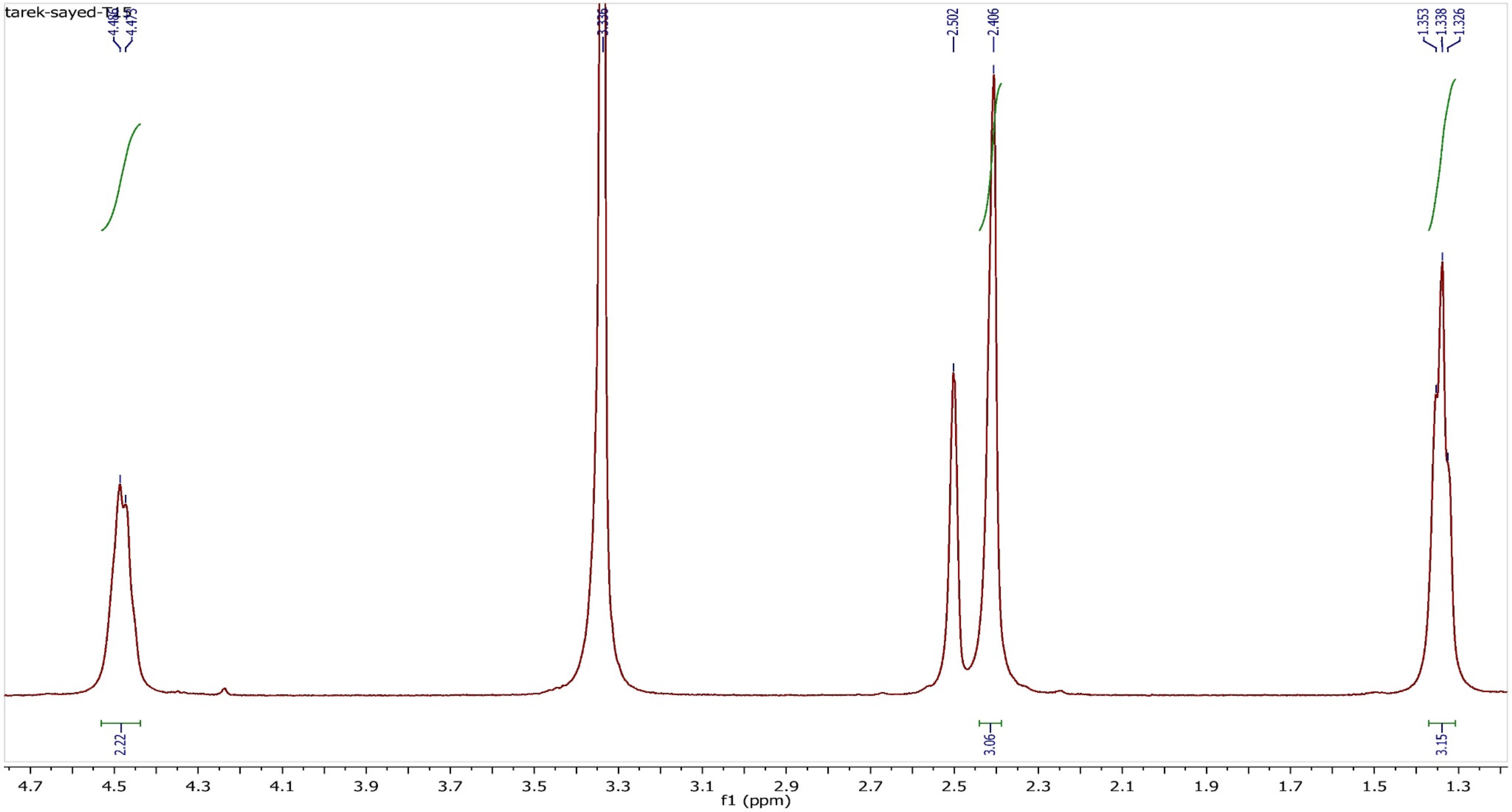

Figure S36: The enlargement of upfield region in ^{1}H-NMR spectrum of compound **4e**.

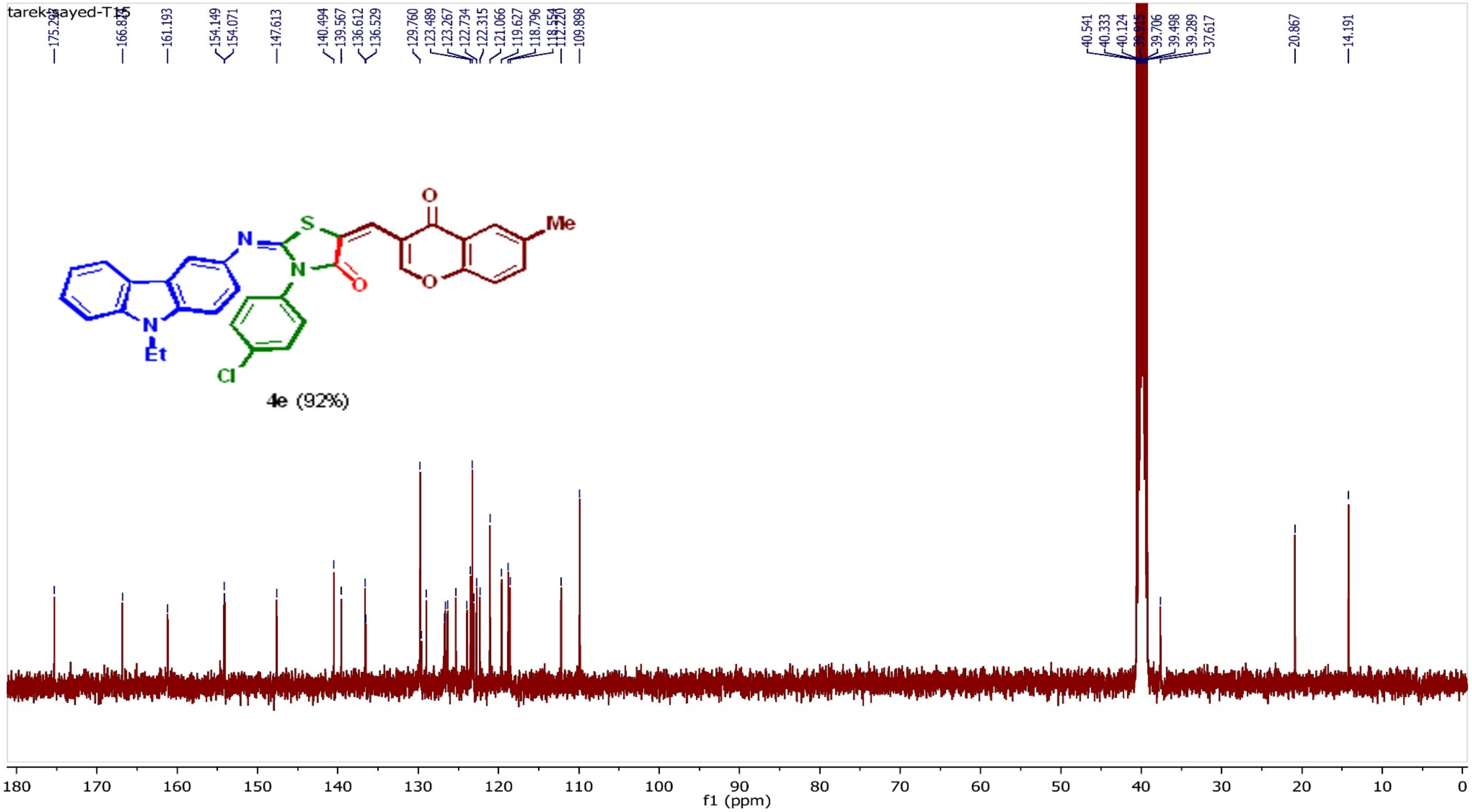

Figure S37: The ^{13}C-NMR spectrum of compound **4e**.

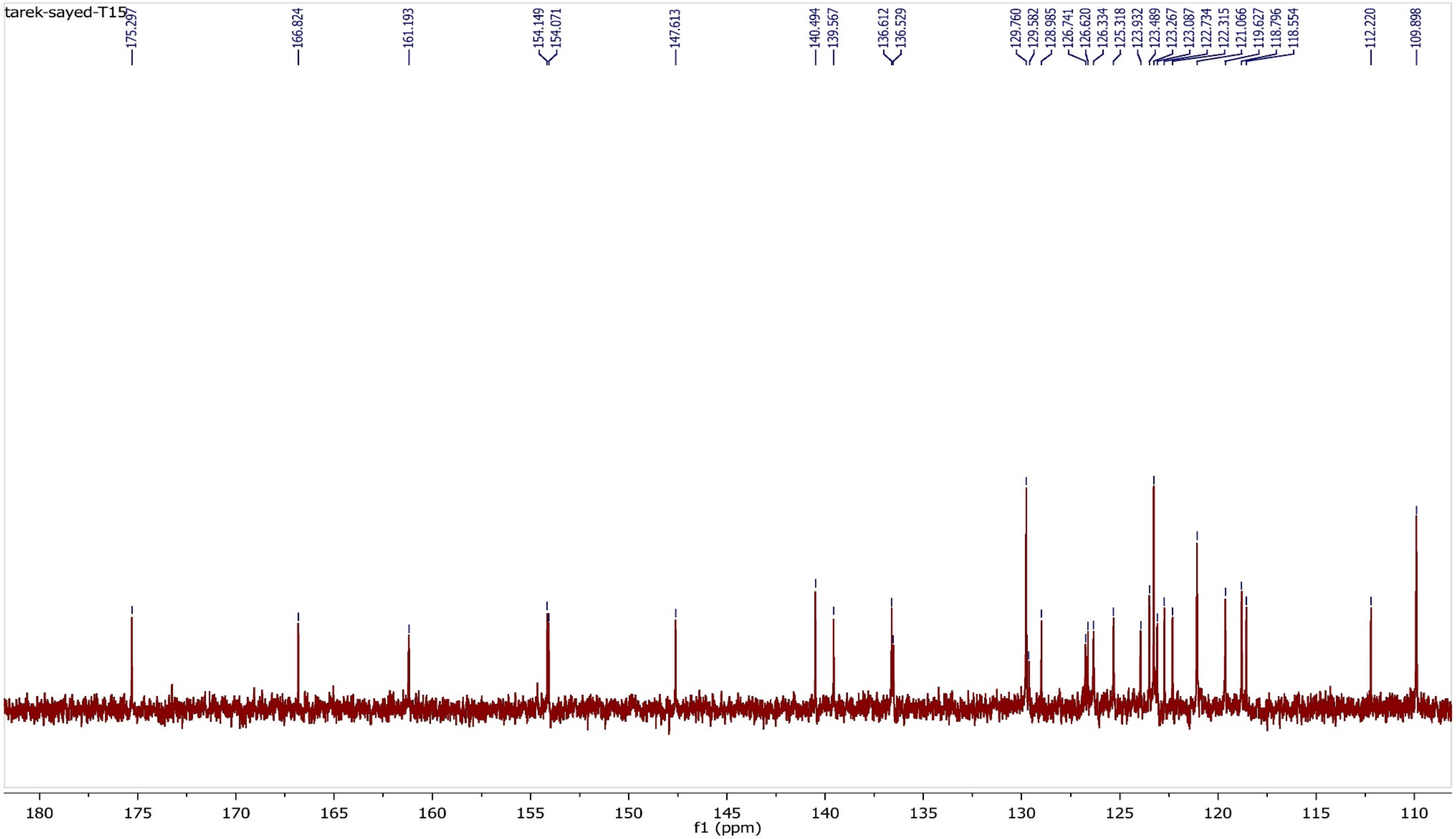

Figure S38: The enlargement of downfield region in ^{13}C-NMR spectrum of compound **4e**.

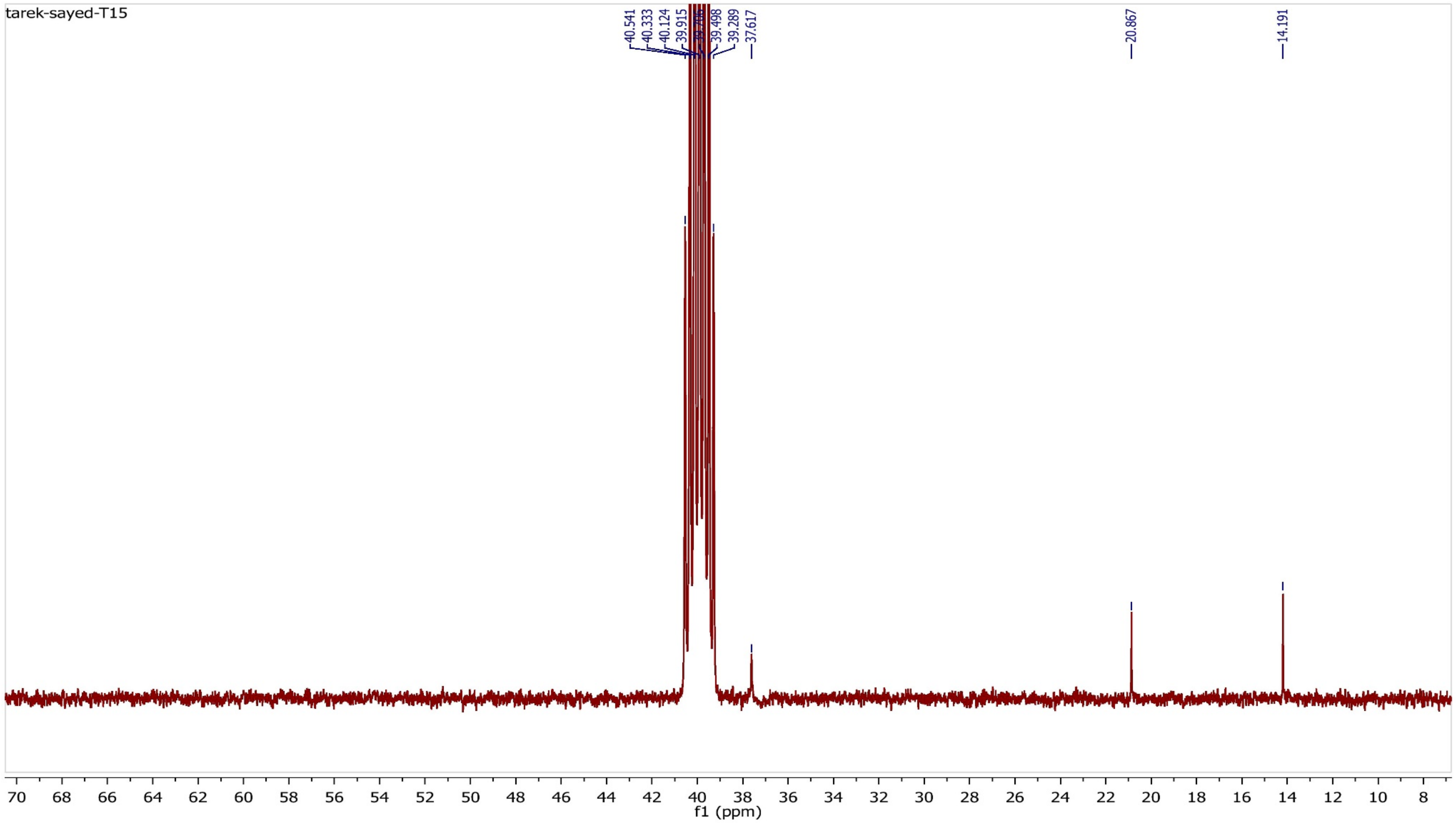

Figure S39: The enlargement of upfield region in ^{13}C-NMR spectrum of compound **4e**.

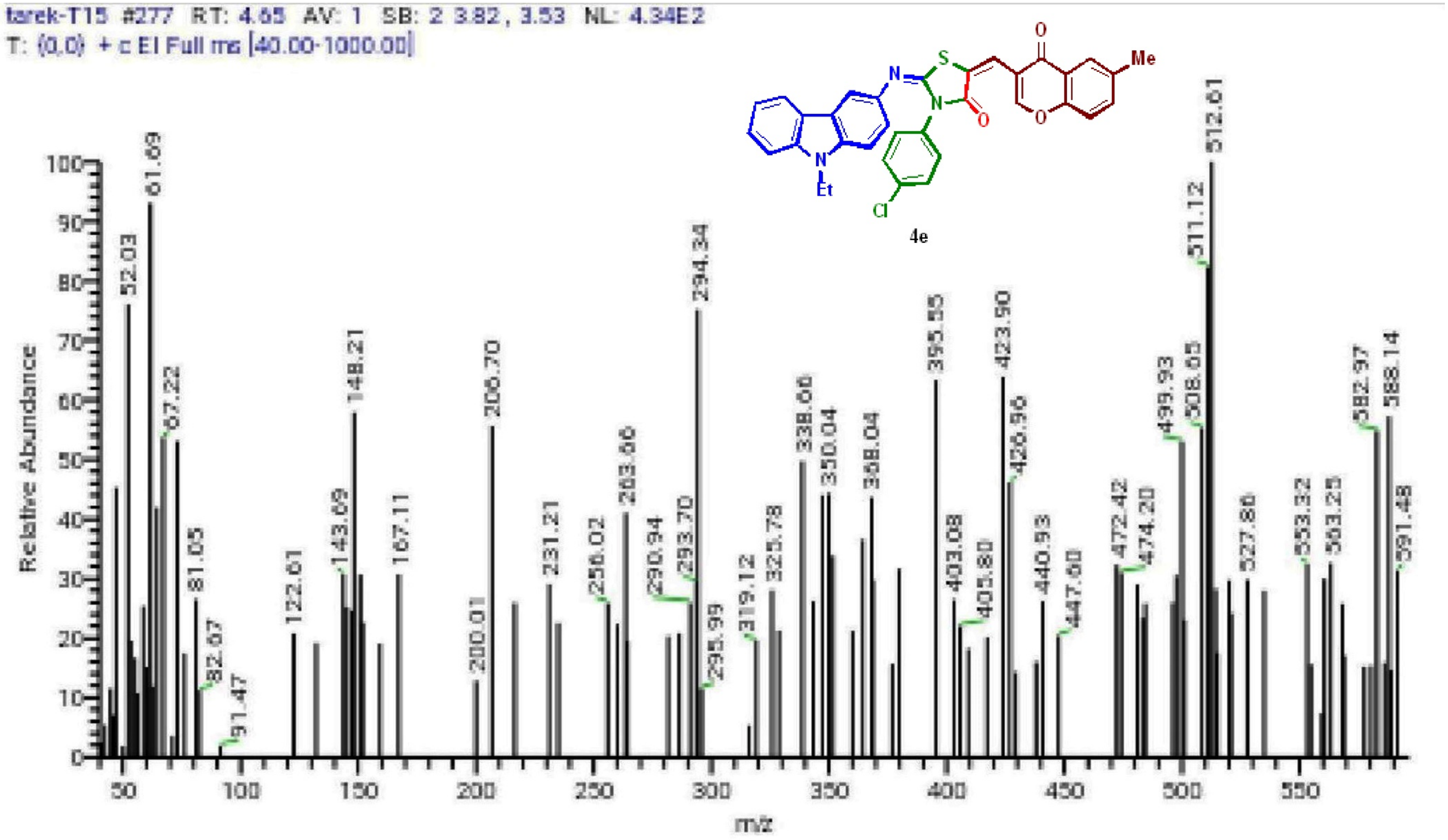

Figure S40: The mass spectrum of compound **4e**.

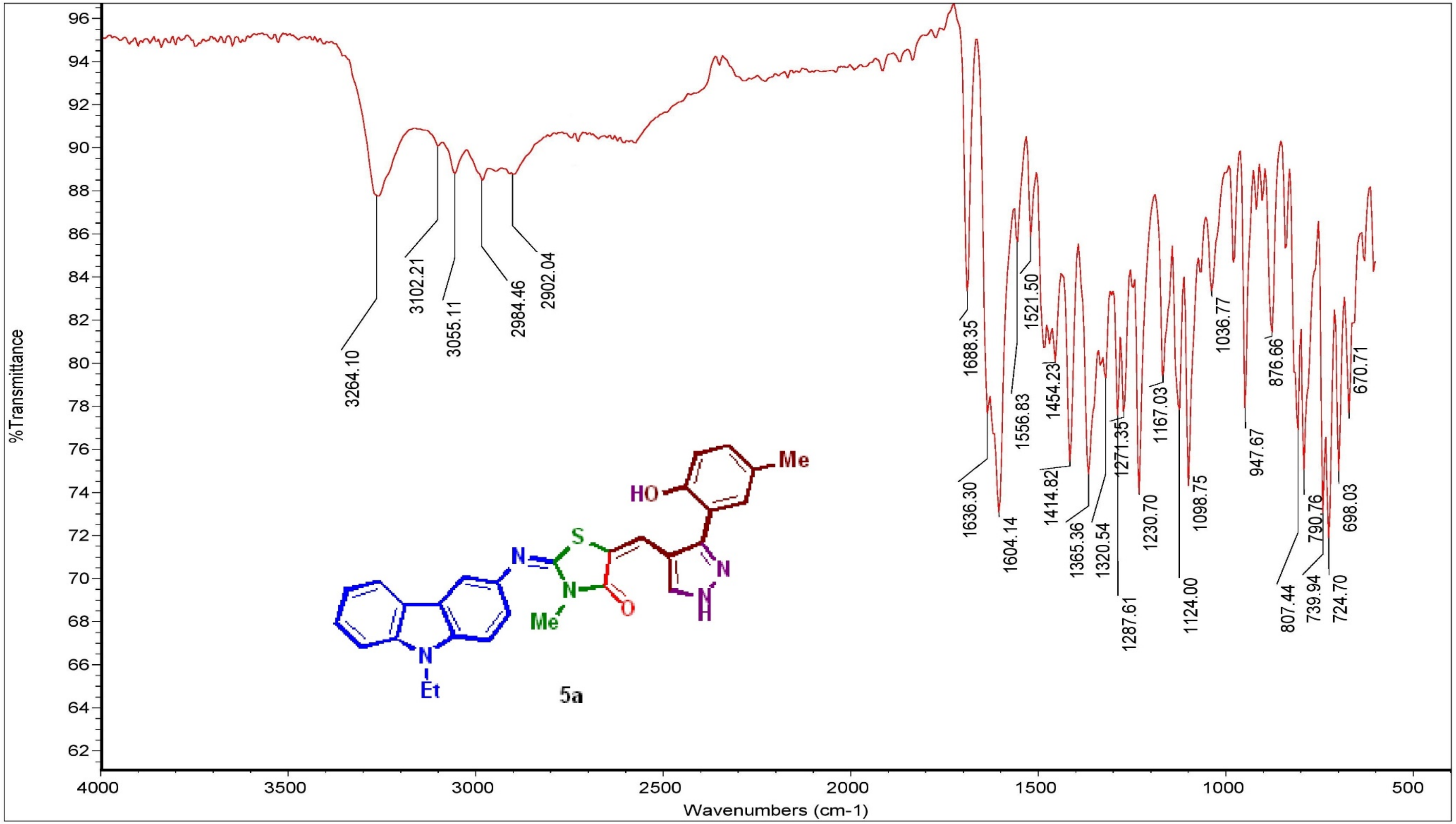

Figure S41: The IR spectrum of compound **5a**.

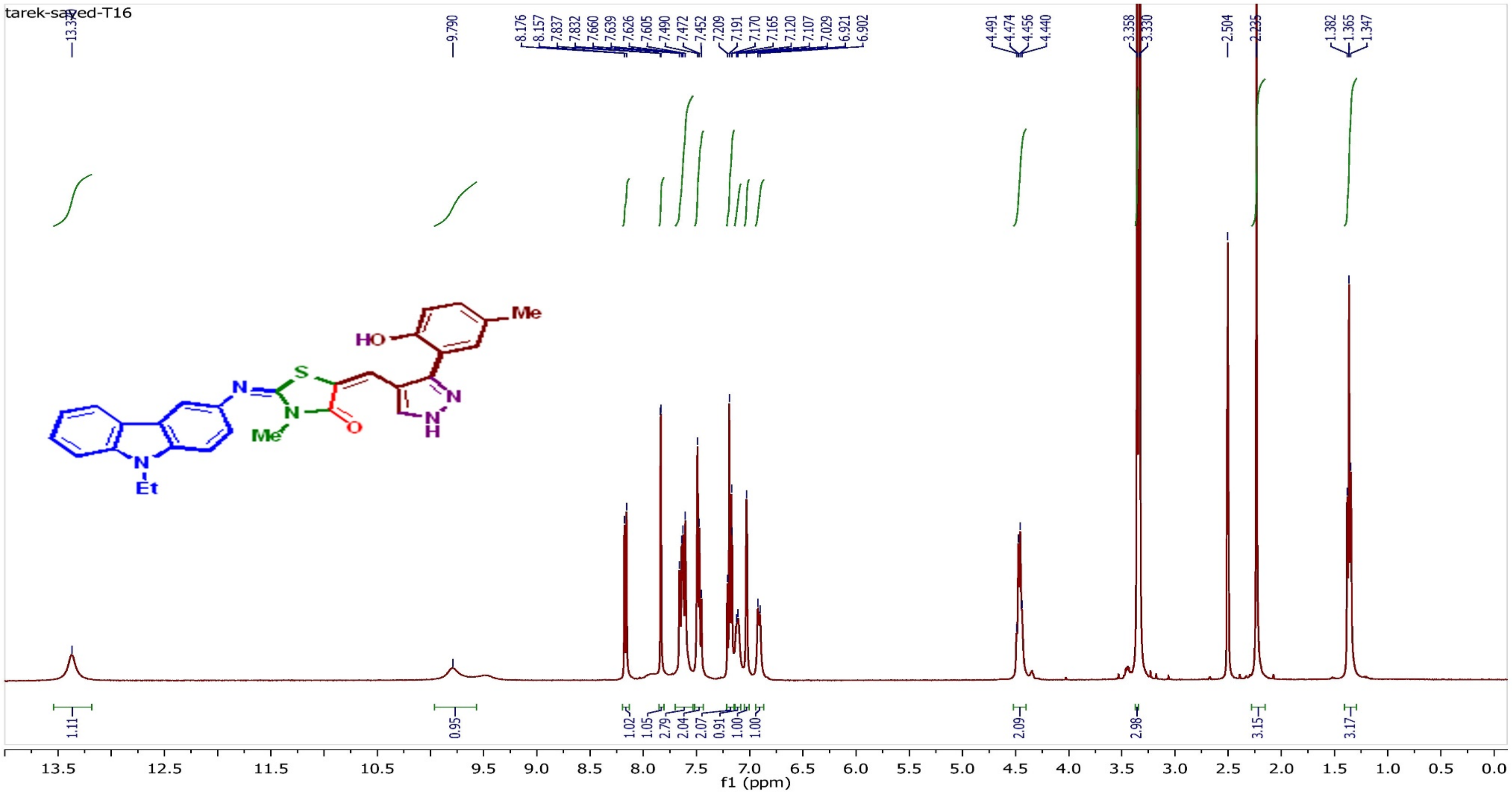

Figure S42: The ^{1}H-NMR spectrum of compound **5a**.

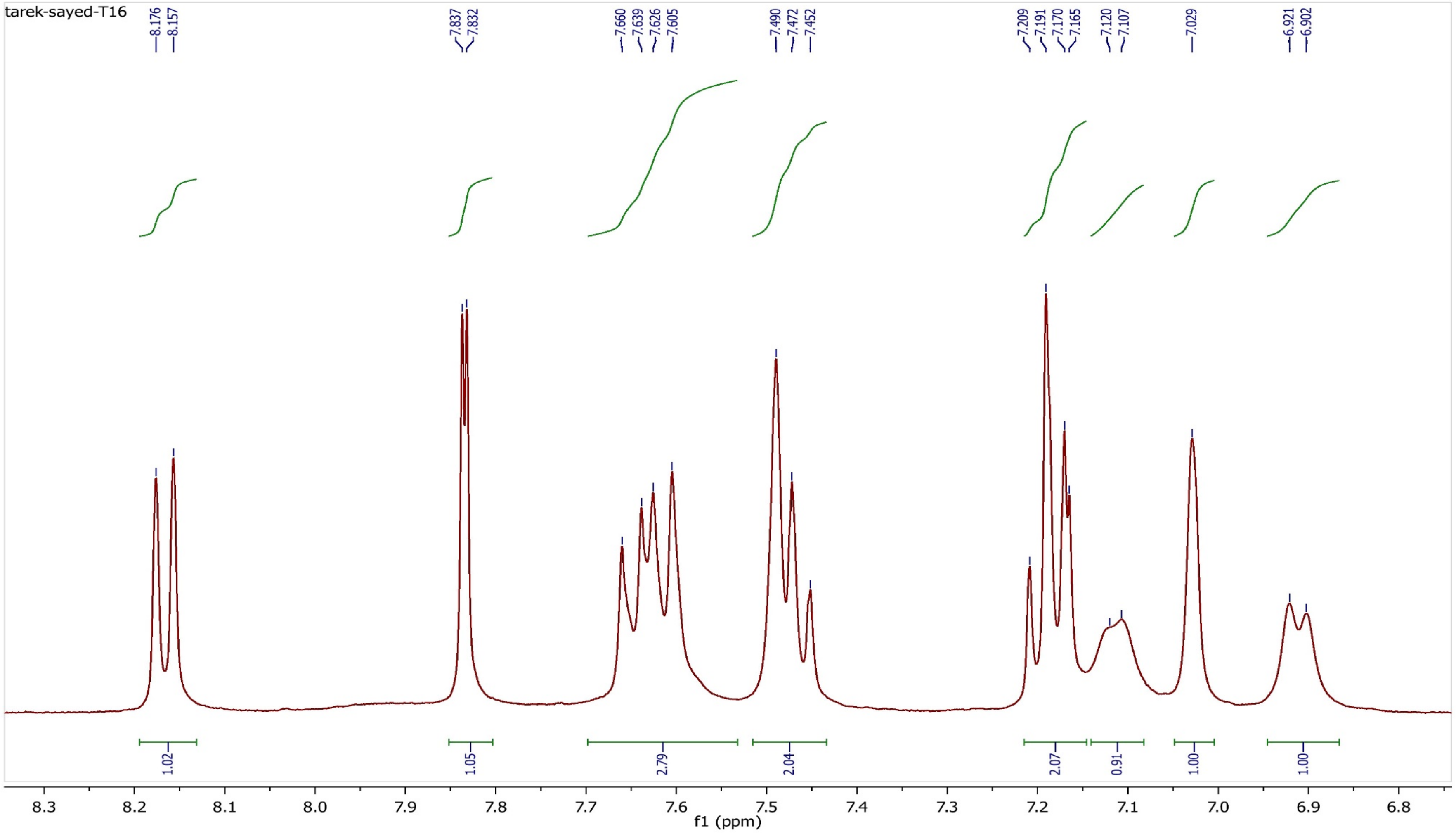

Figure S43: The enlargement of downfield region in ^{1}H-NMR spectrum of compound **5a**.

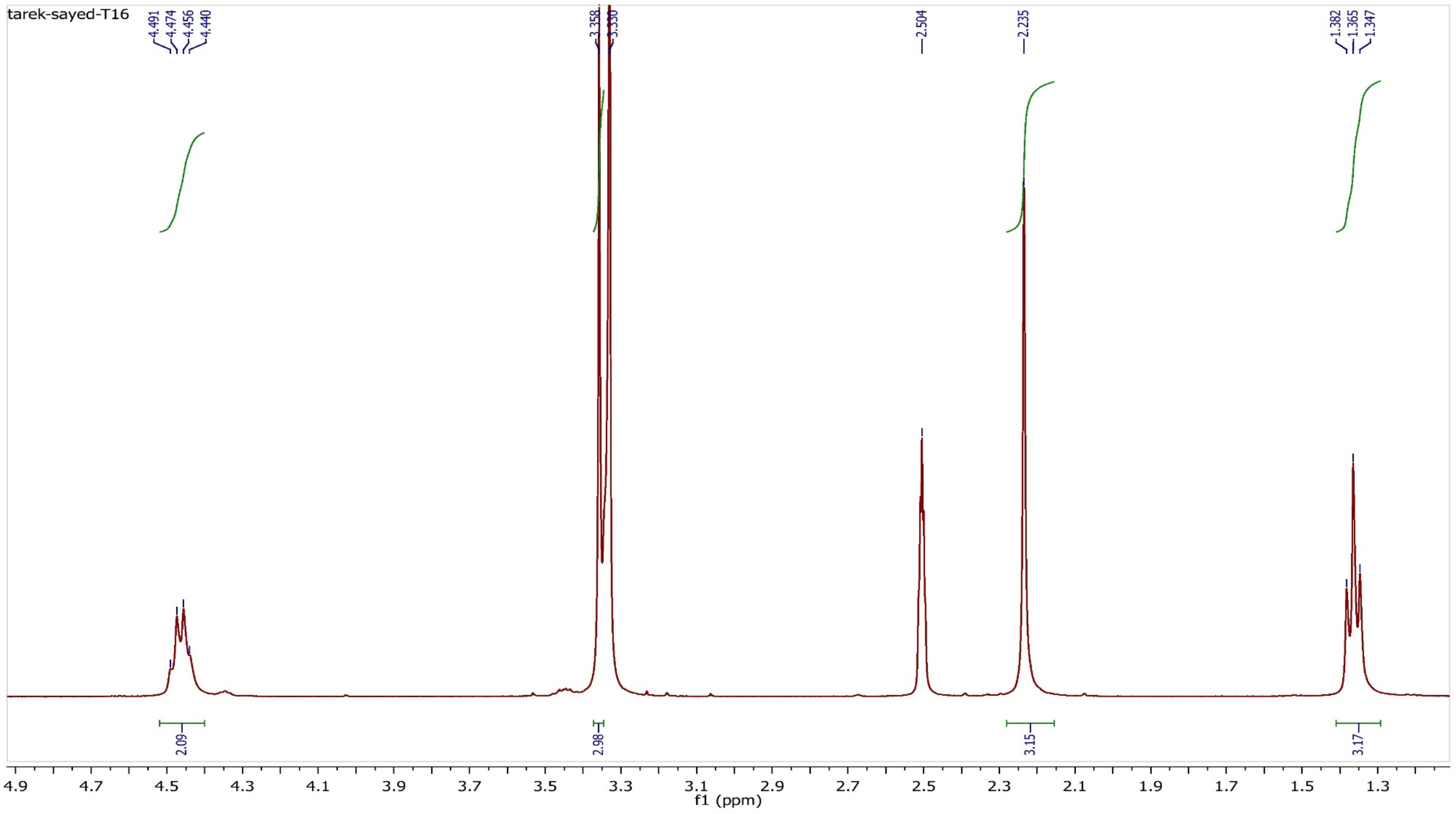

Figure S44: The enlargement of upfield region in ^{1}H-NMR spectrum of compound **5a**.

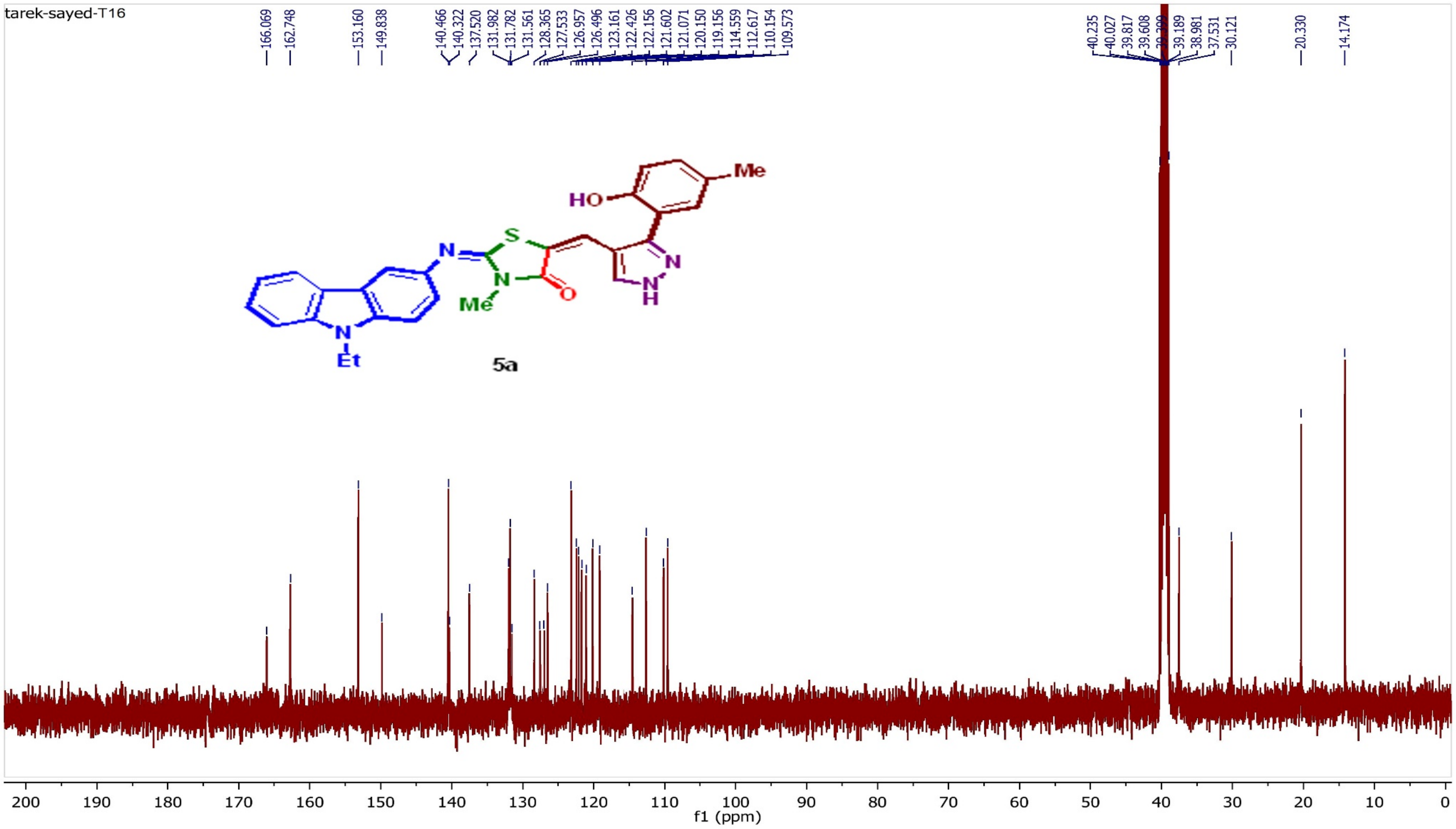

Figure S45: The ^{13}C-NMR spectrum of compound **5a**.

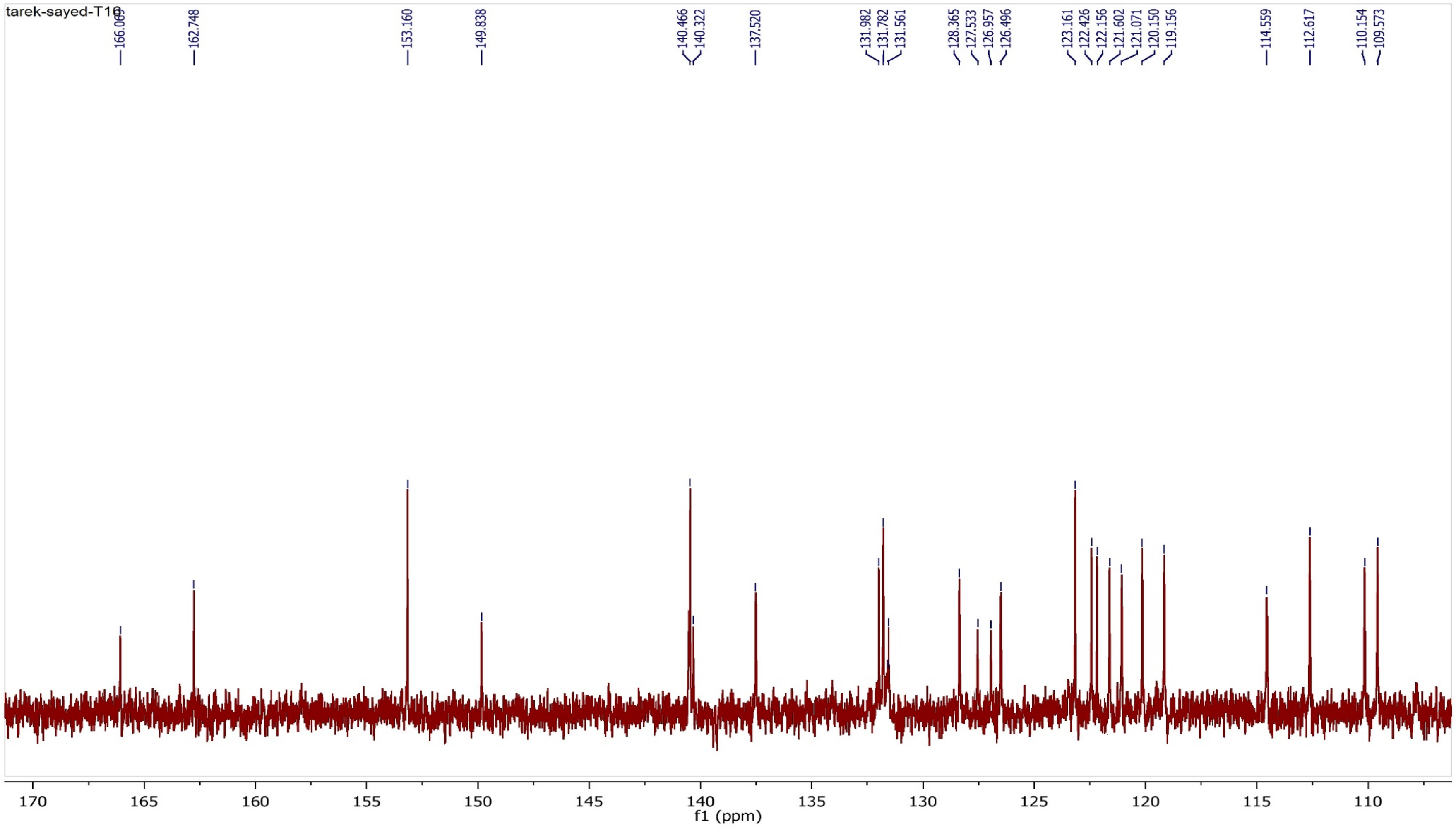

Figure S46: The enlargement of downfield region in ^{13}C-NMR spectrum of compound **5a**.

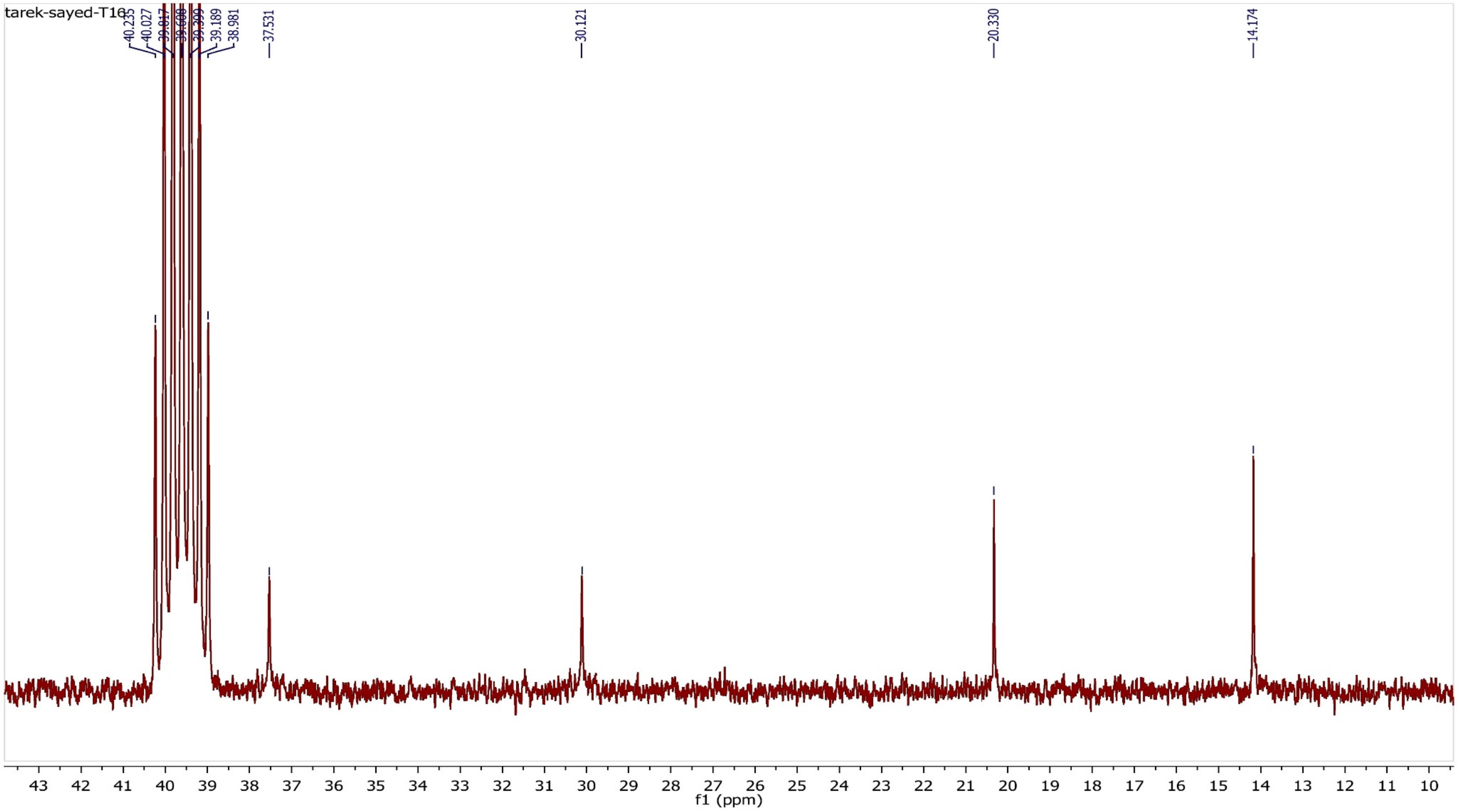

Figure S47: The enlargement of upfield region in ^{13}C-NMR spectrum of compound **5a**.

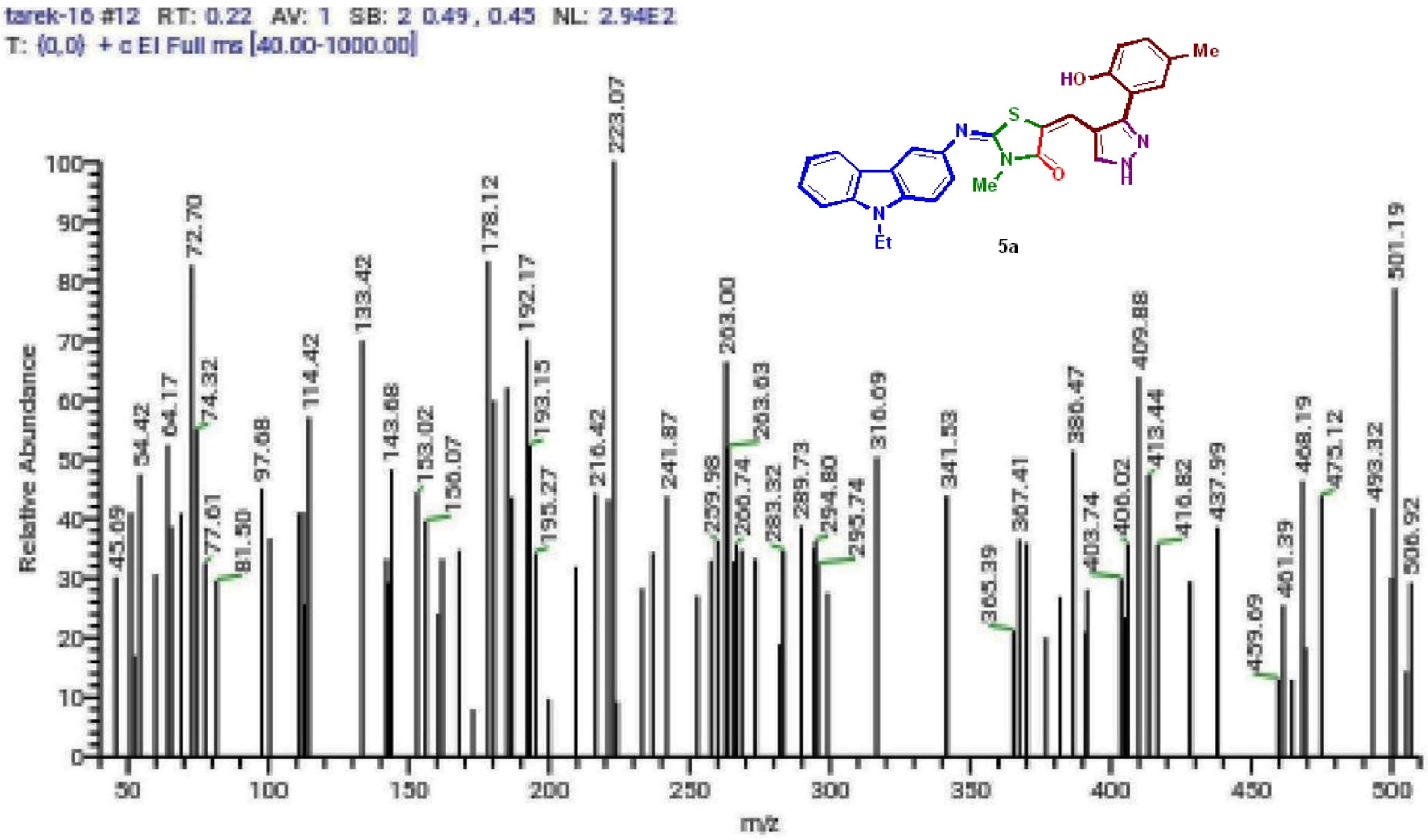

Figure S48: The mass spectrum of compound **5a**.

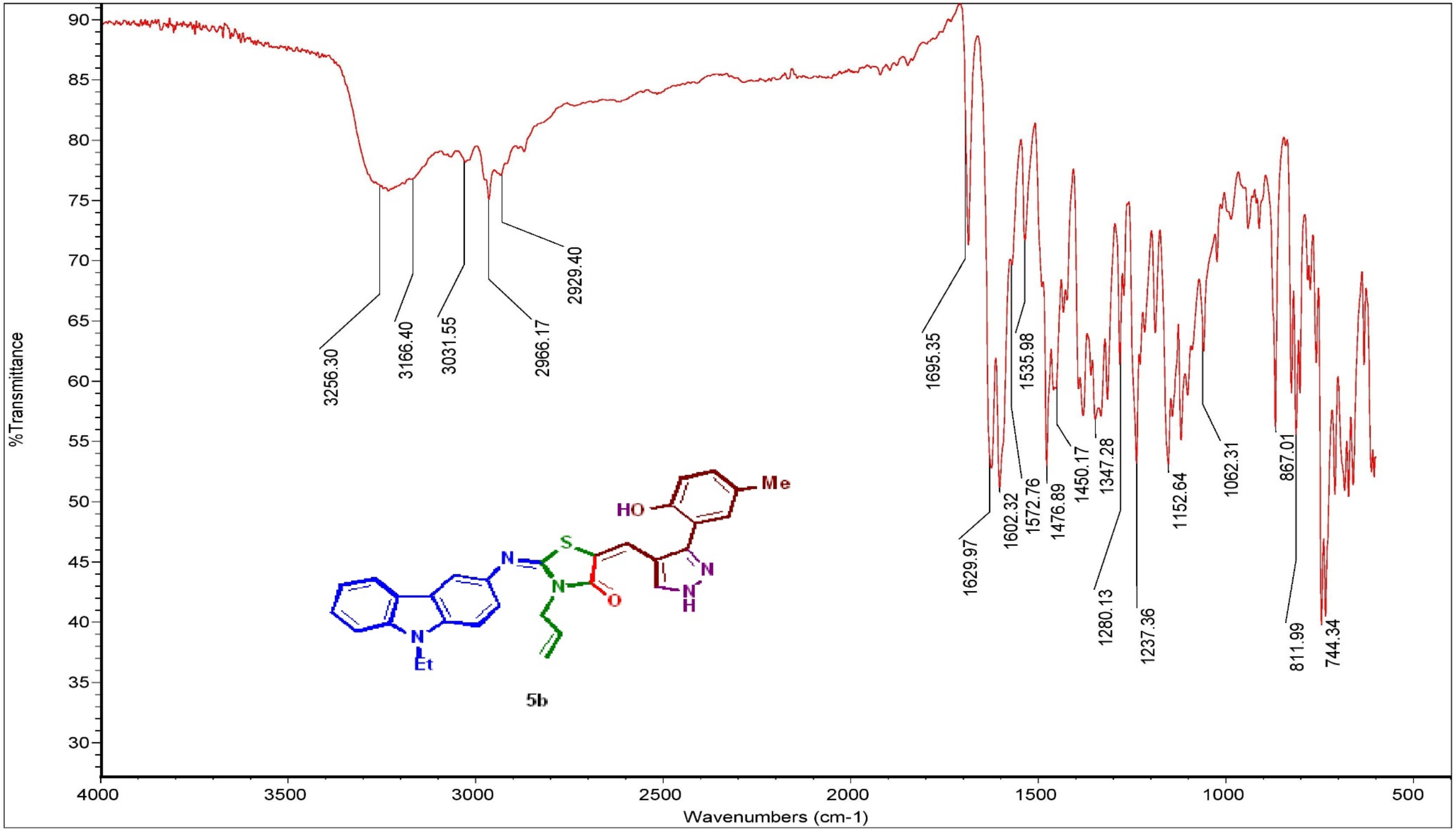

Figure S49: The IR spectrum of compound **5b**.

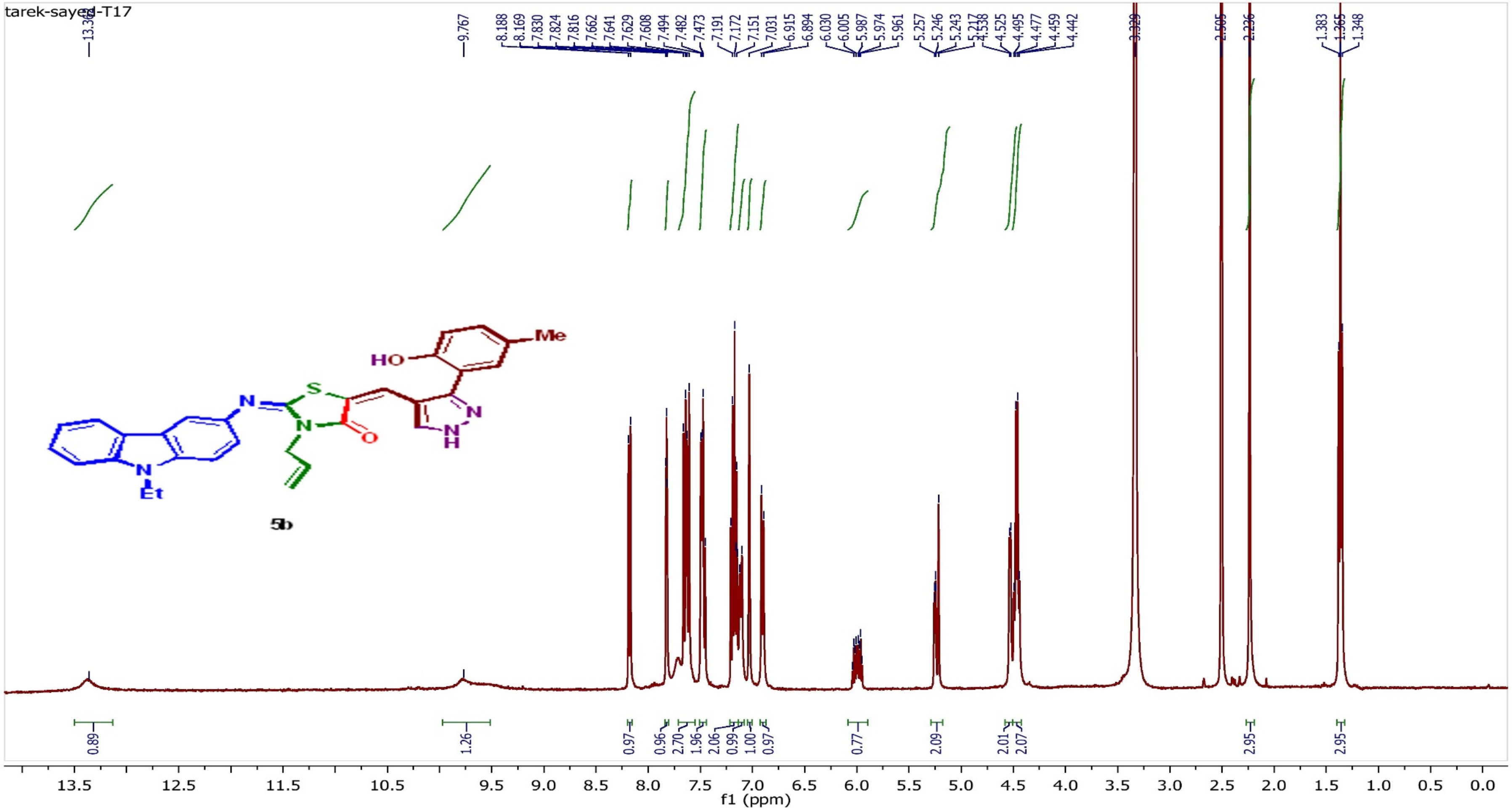

Figure S50: The ^{1}H-NMR spectrum of compound **5b**.

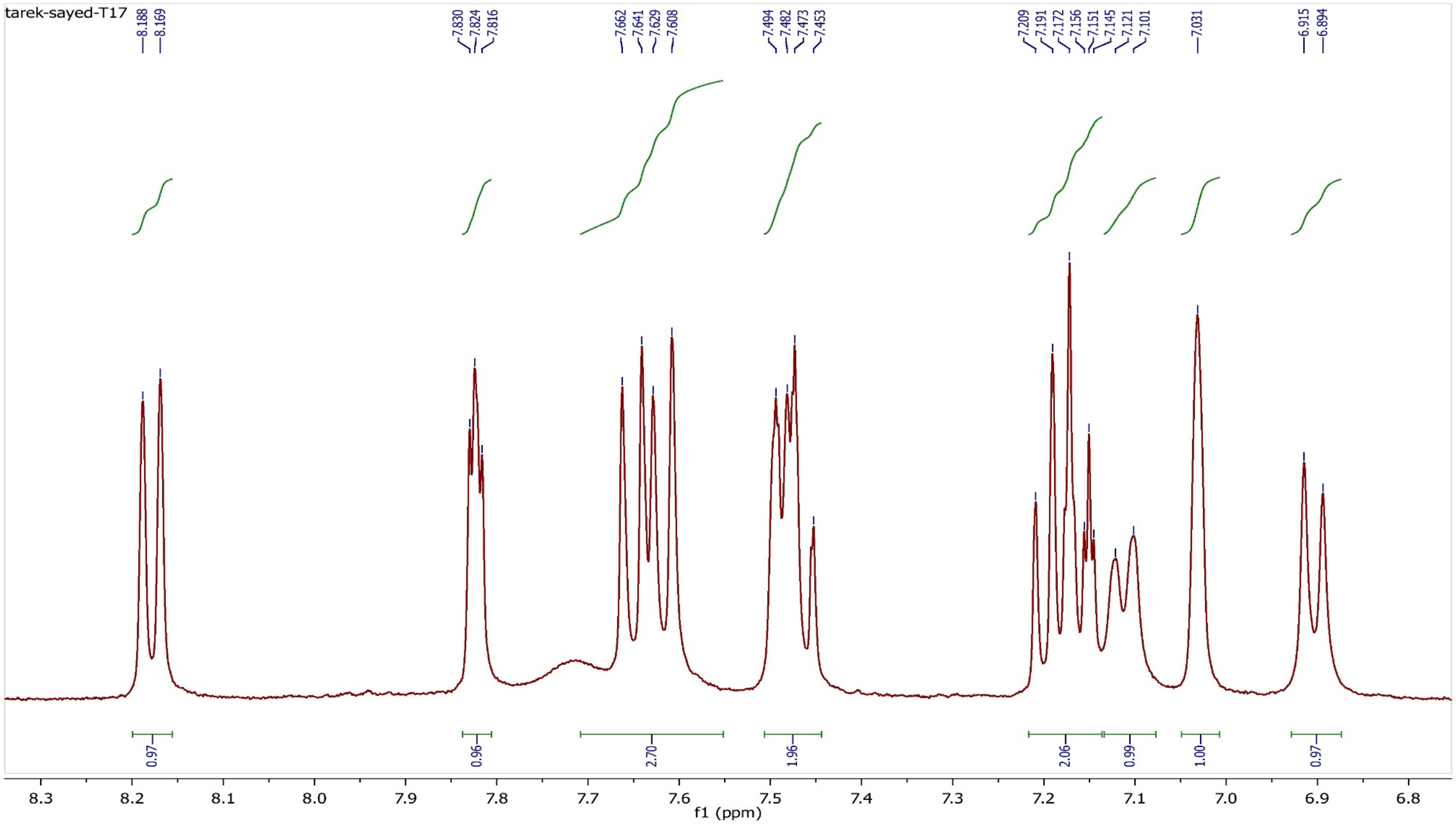

Figure S51: The enlargement of downfield region in ^{1}H-NMR spectrum of compound **5b**.

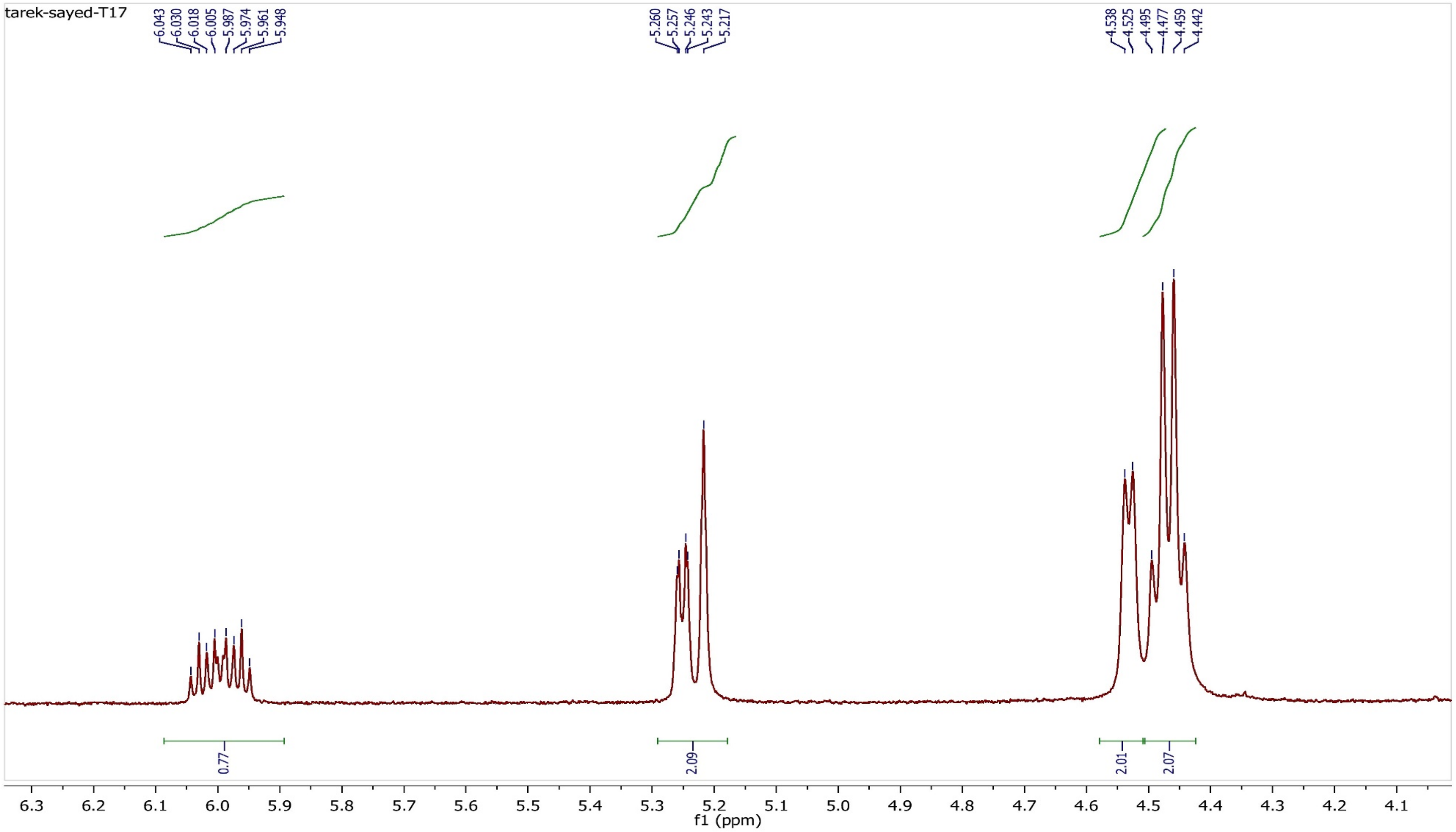

Figure S52: The enlargement of upfield region in ^{1}H-NMR spectrum of compound **5b**.

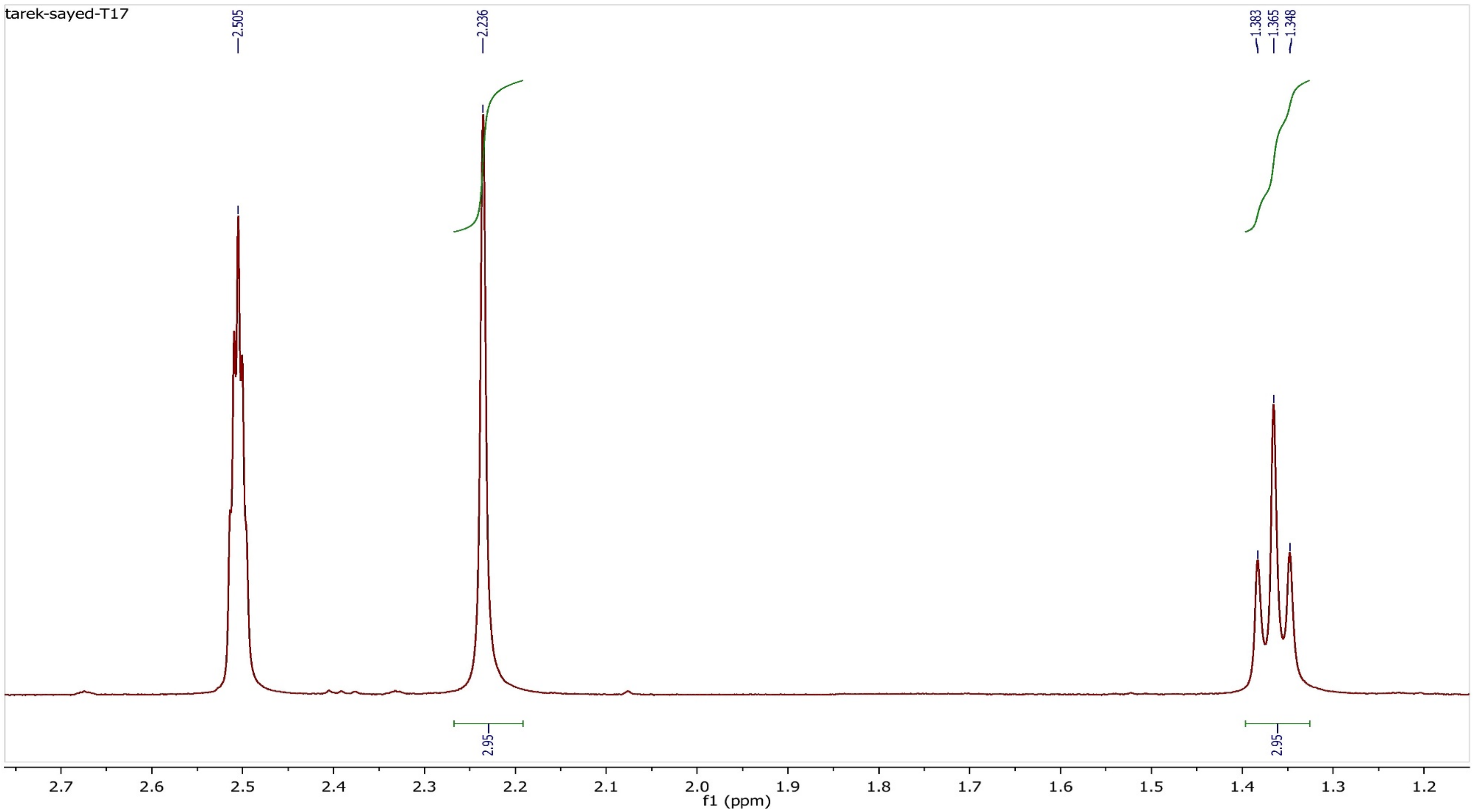

Figure S53: The enlargement of upfield region in ^{1}H-NMR spectrum of compound **5b**.

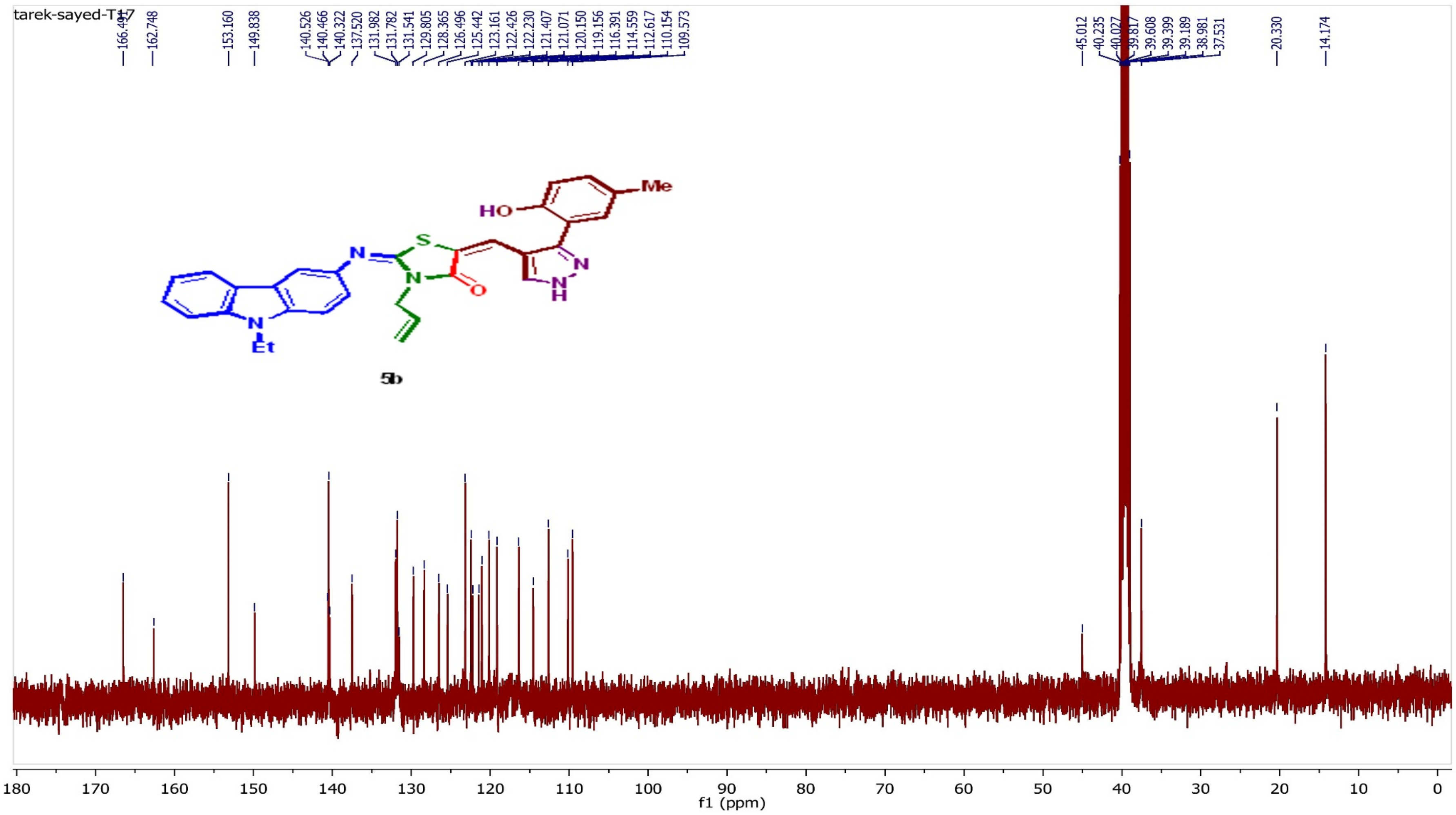

Figure S54: The ^{13}C-NMR spectrum of compound **5b**.

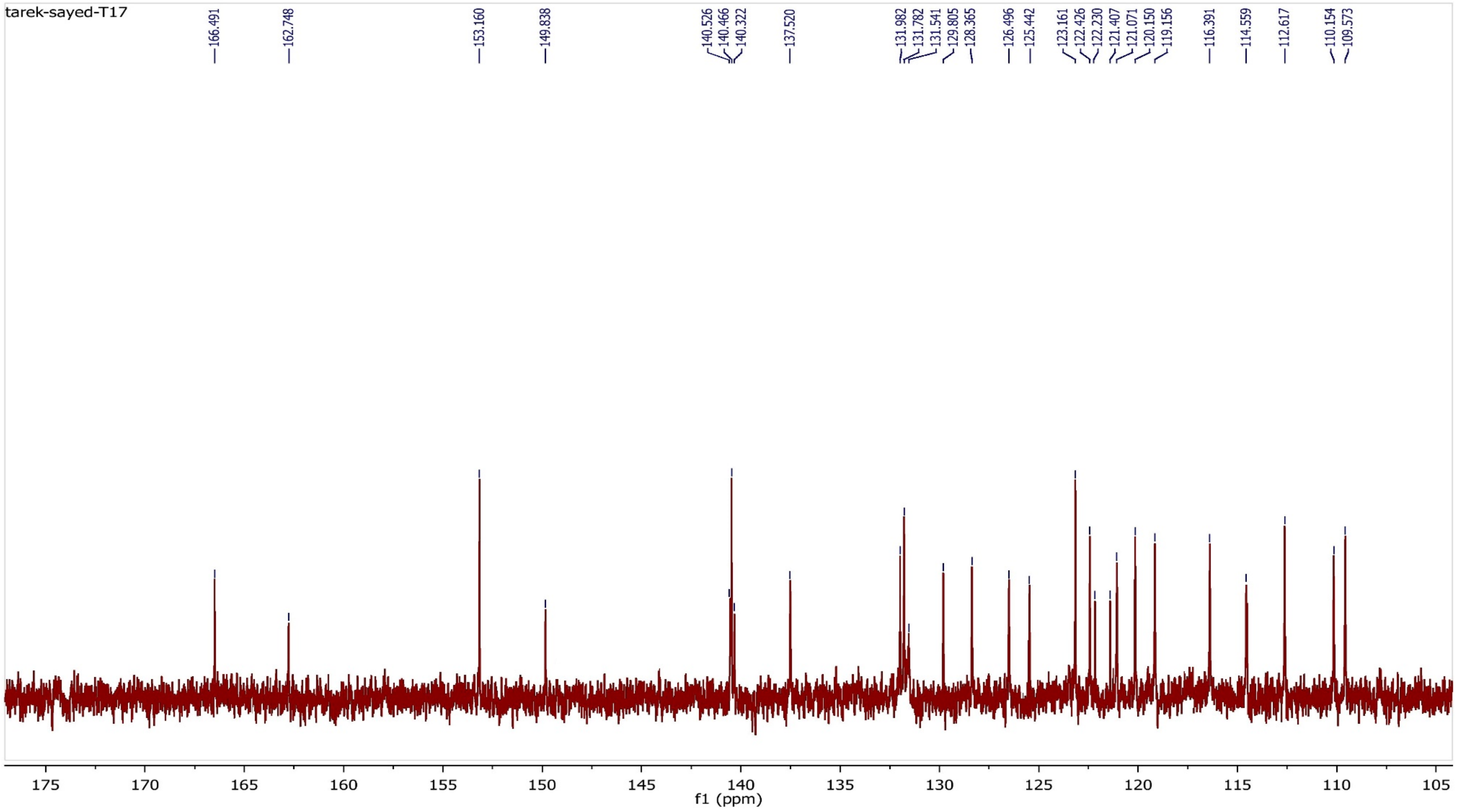

Figure S55: The enlargement of downfield region in ^{13}C-NMR spectrum of compound **5b**.

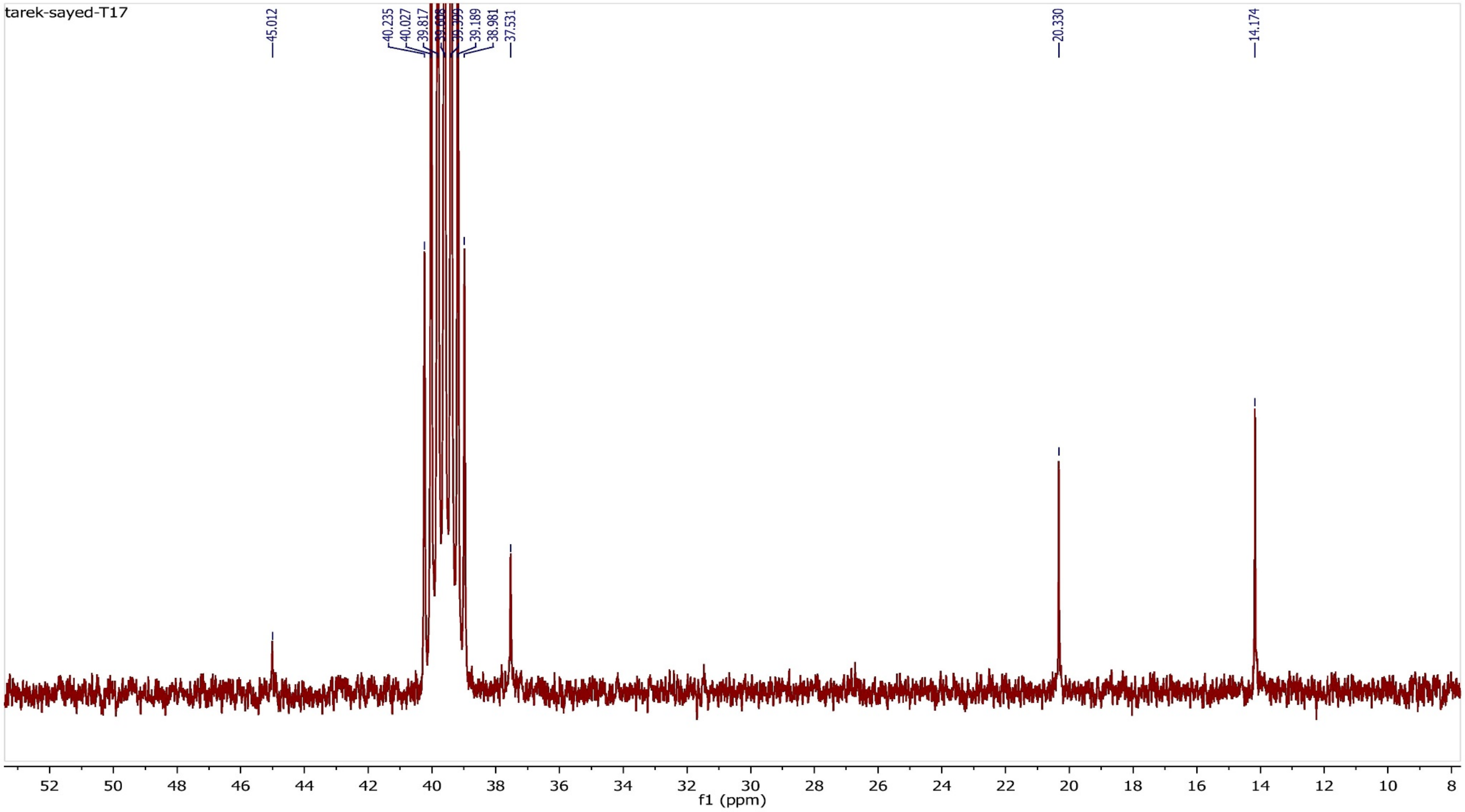

Figure S56: The enlargement of upfield region in ^{13}C-NMR spectrum of compound **5b**.

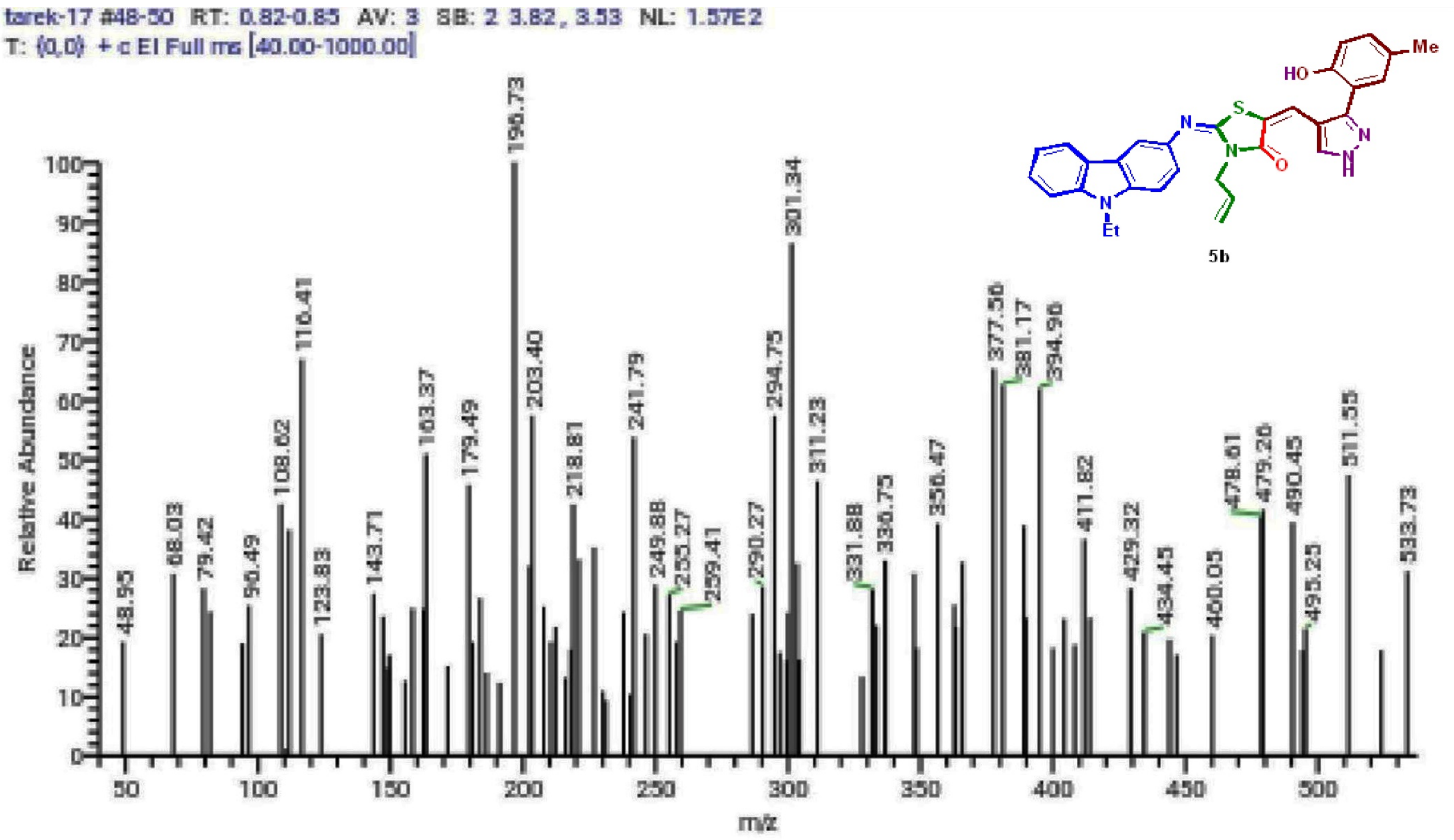

Figure S57: The mass spectrum of compound **5b**.

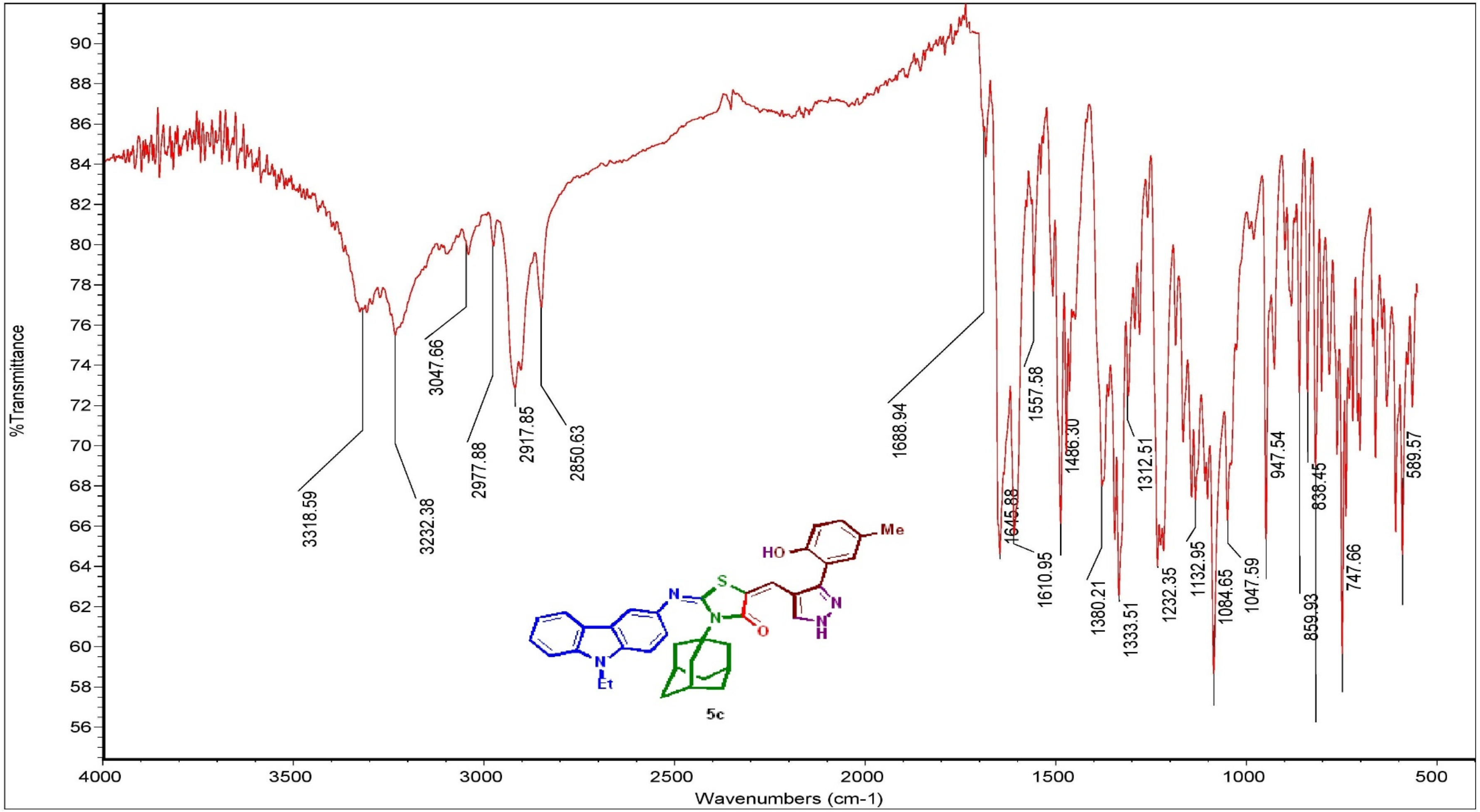

Figure S58: The IR spectrum of compound **5c**.

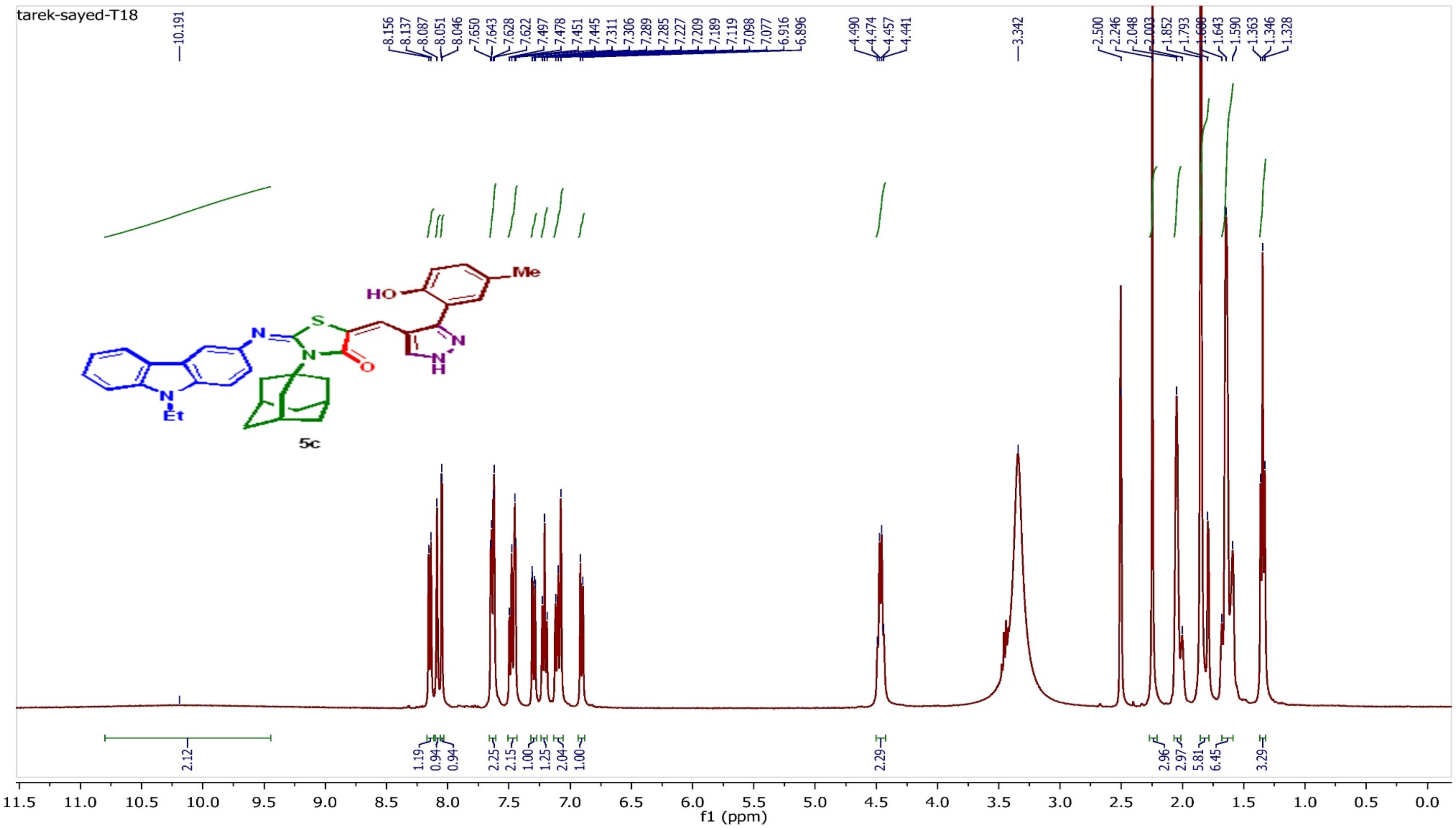

Figure S59: The ^{1}H-NMR spectrum of compound **5c**.

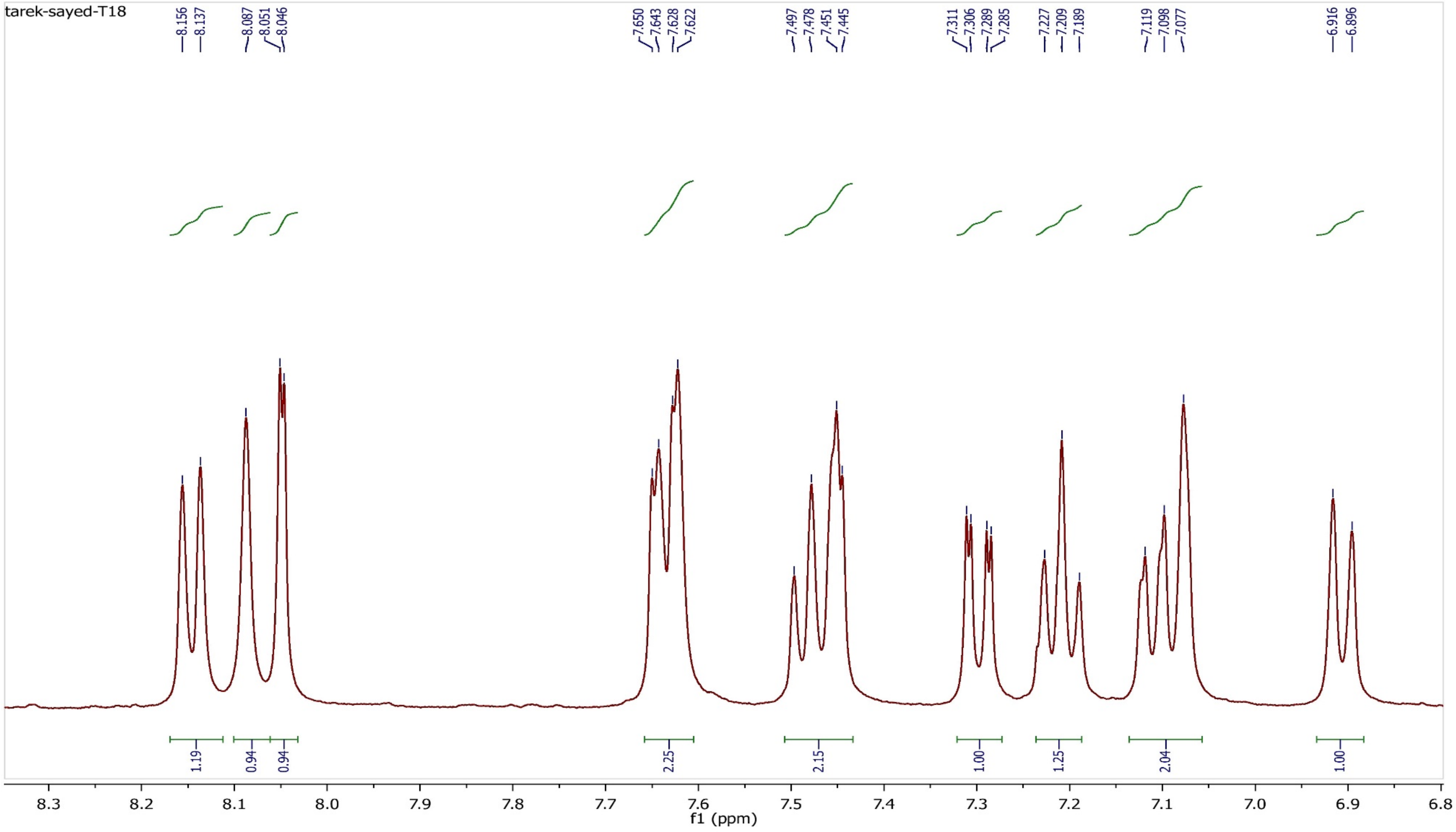

Figure S60: The enlargement of downfield region in ^{1}H-NMR spectrum of compound **5c**.

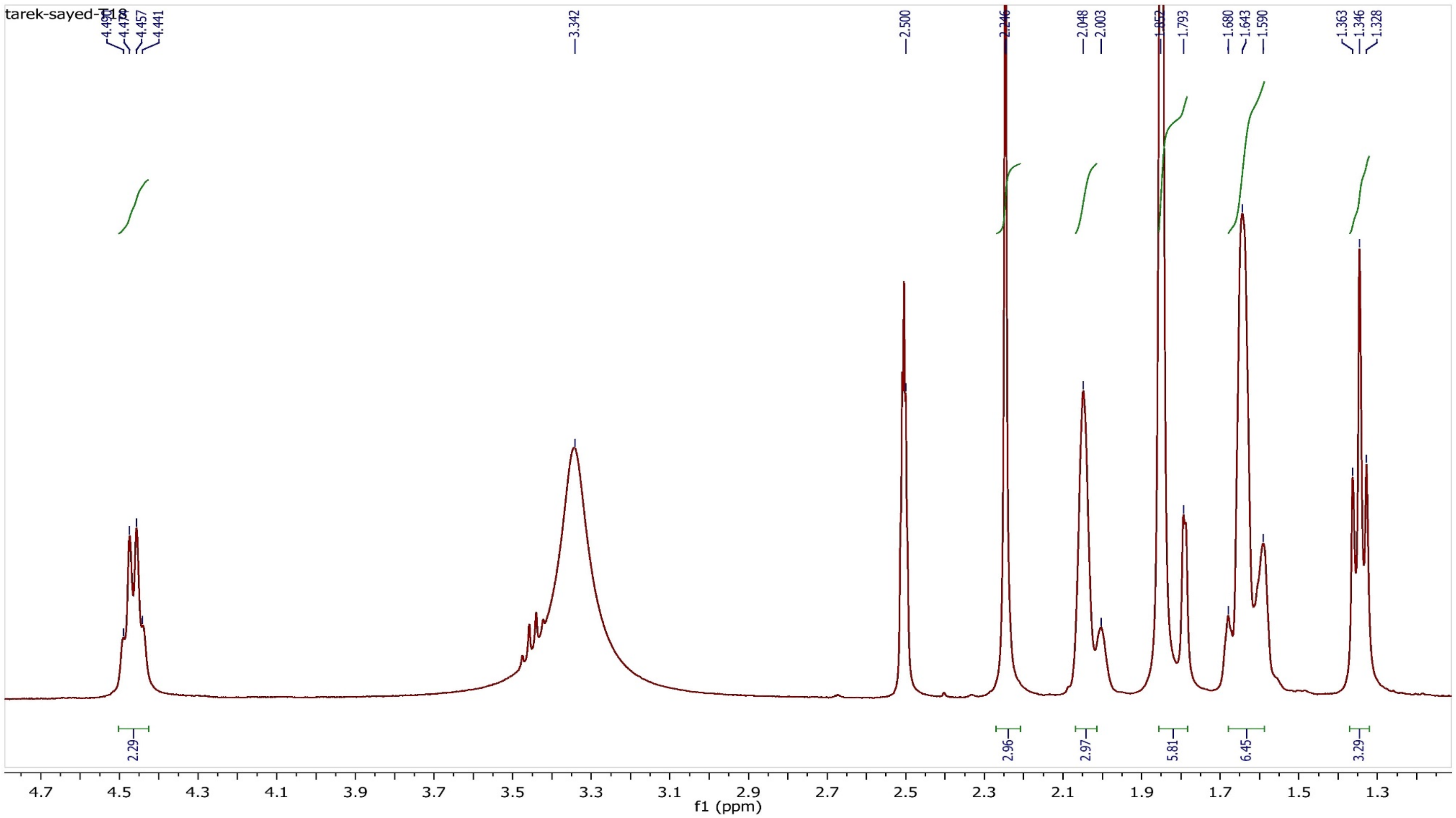

Figure S61: The enlargement of upfield region in ^{1}H-NMR spectrum of compound **5c**.

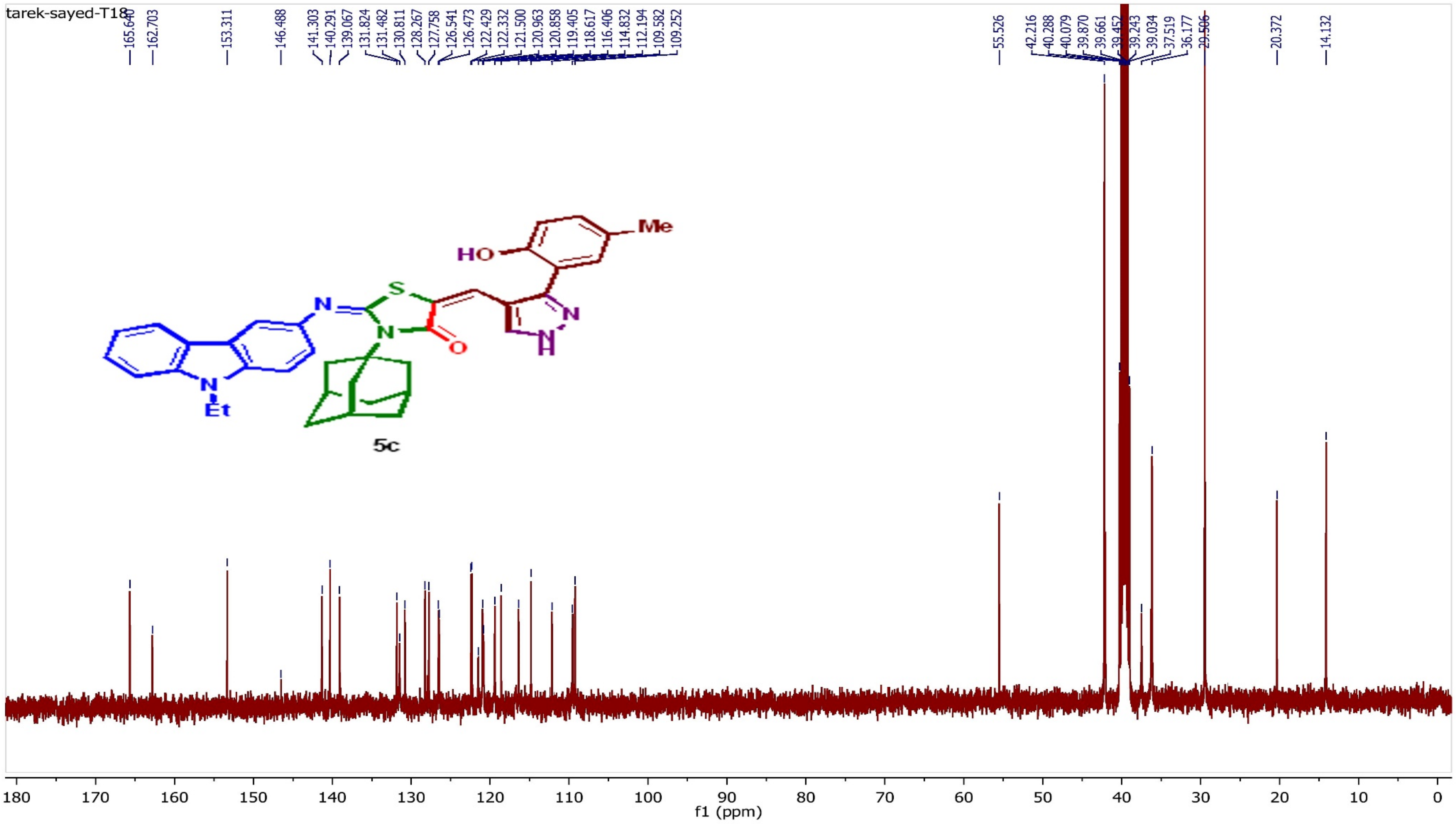

Figure S62: The ^{13}C-NMR spectrum of compound **5c**.

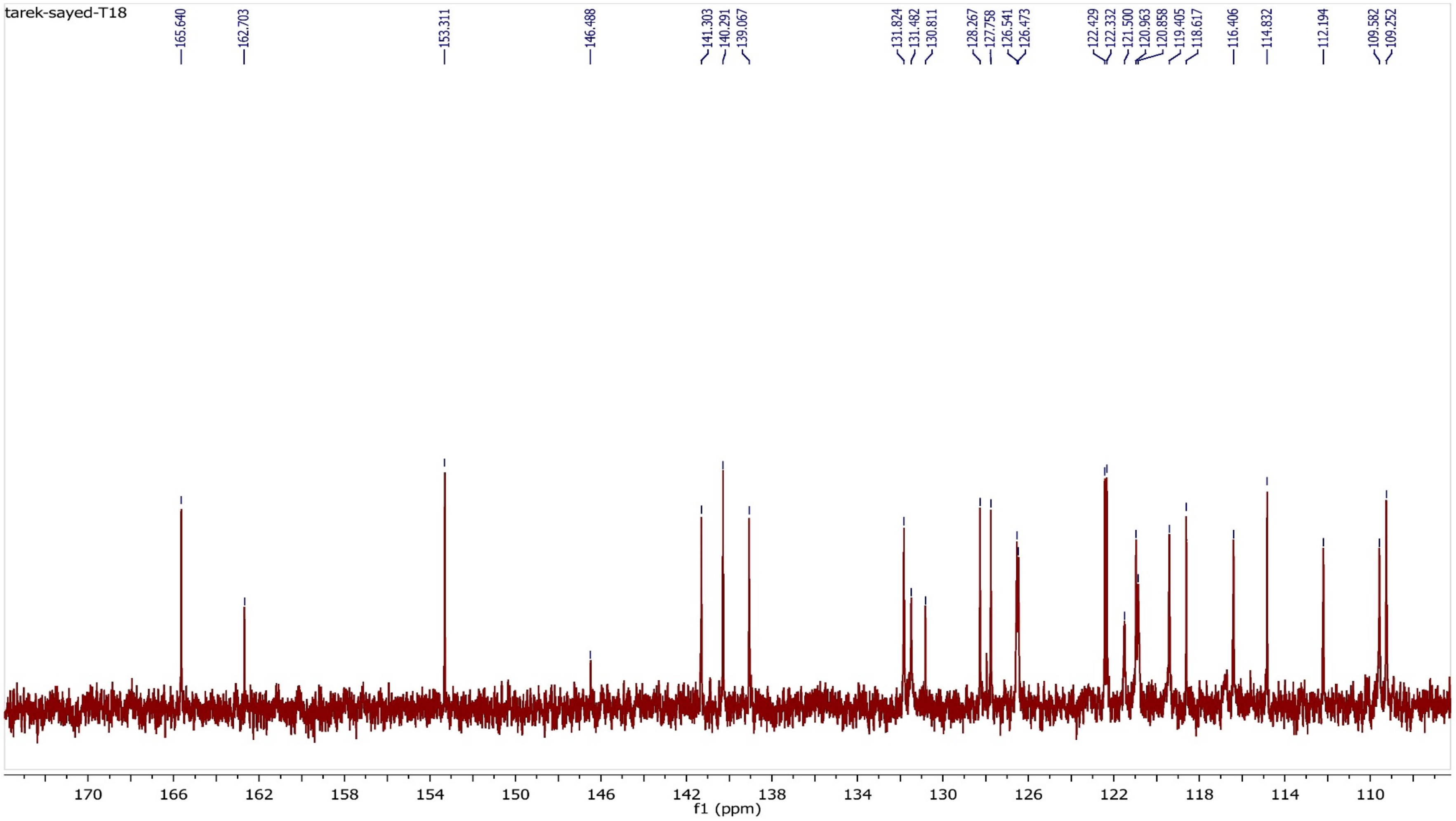

Figure S63: The enlargement of downfield region in ^{13}C-NMR spectrum of compound **5c**.

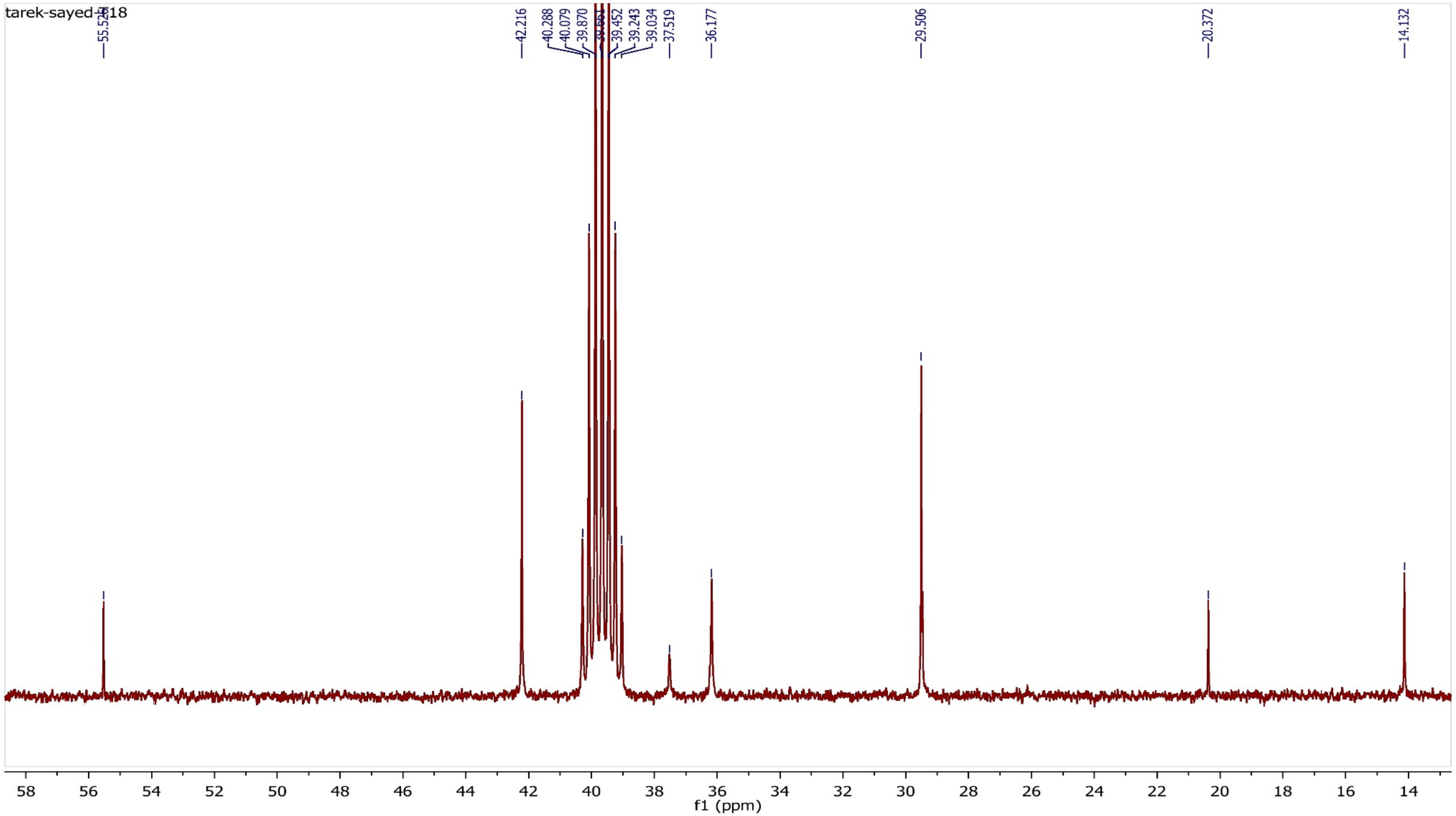

Figure S64: The enlargement of upfield region in ^{13}C-NMR spectrum of compound **5c**.

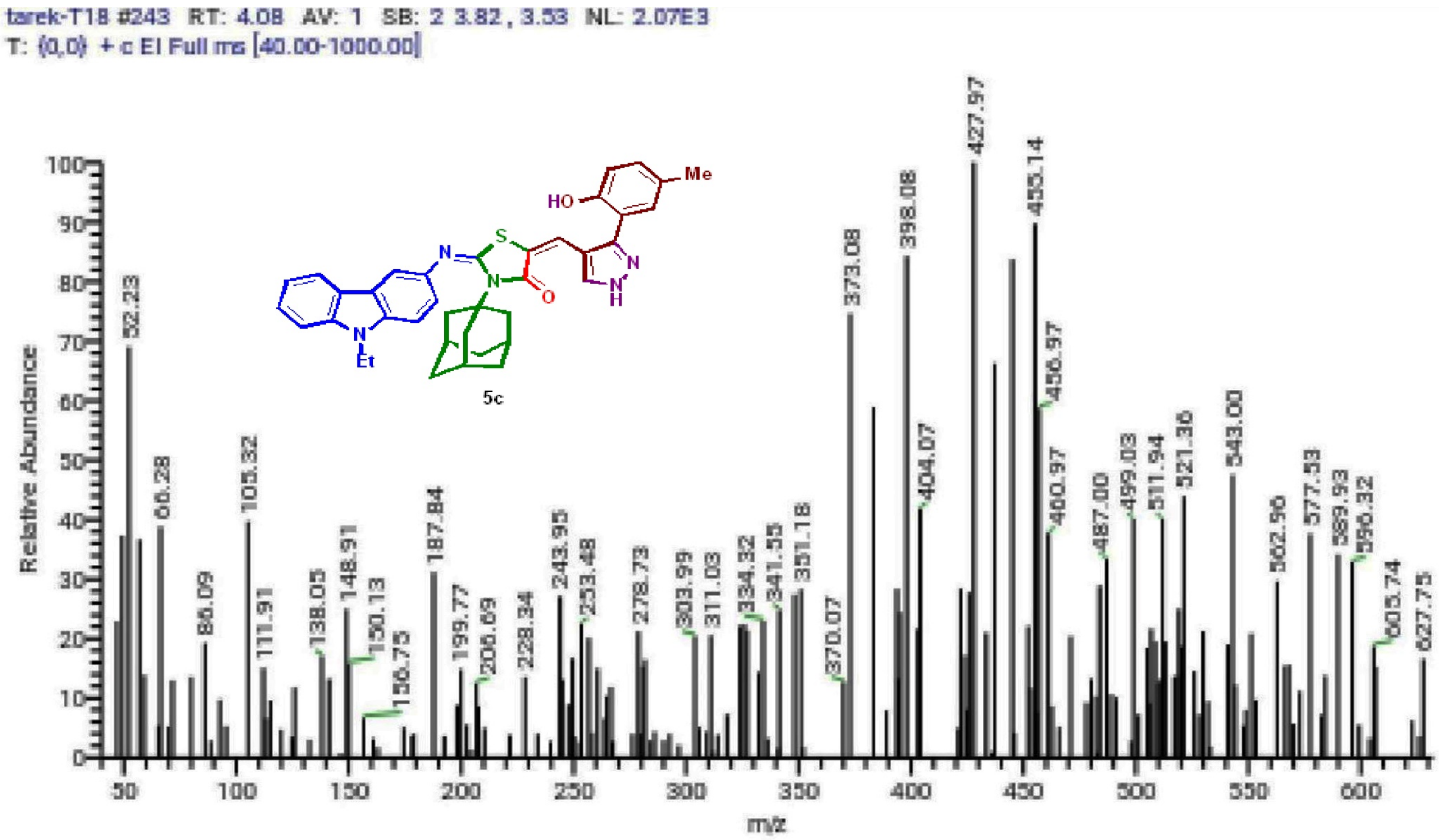

Figure S65: The mass spectrum of compound **5c**.

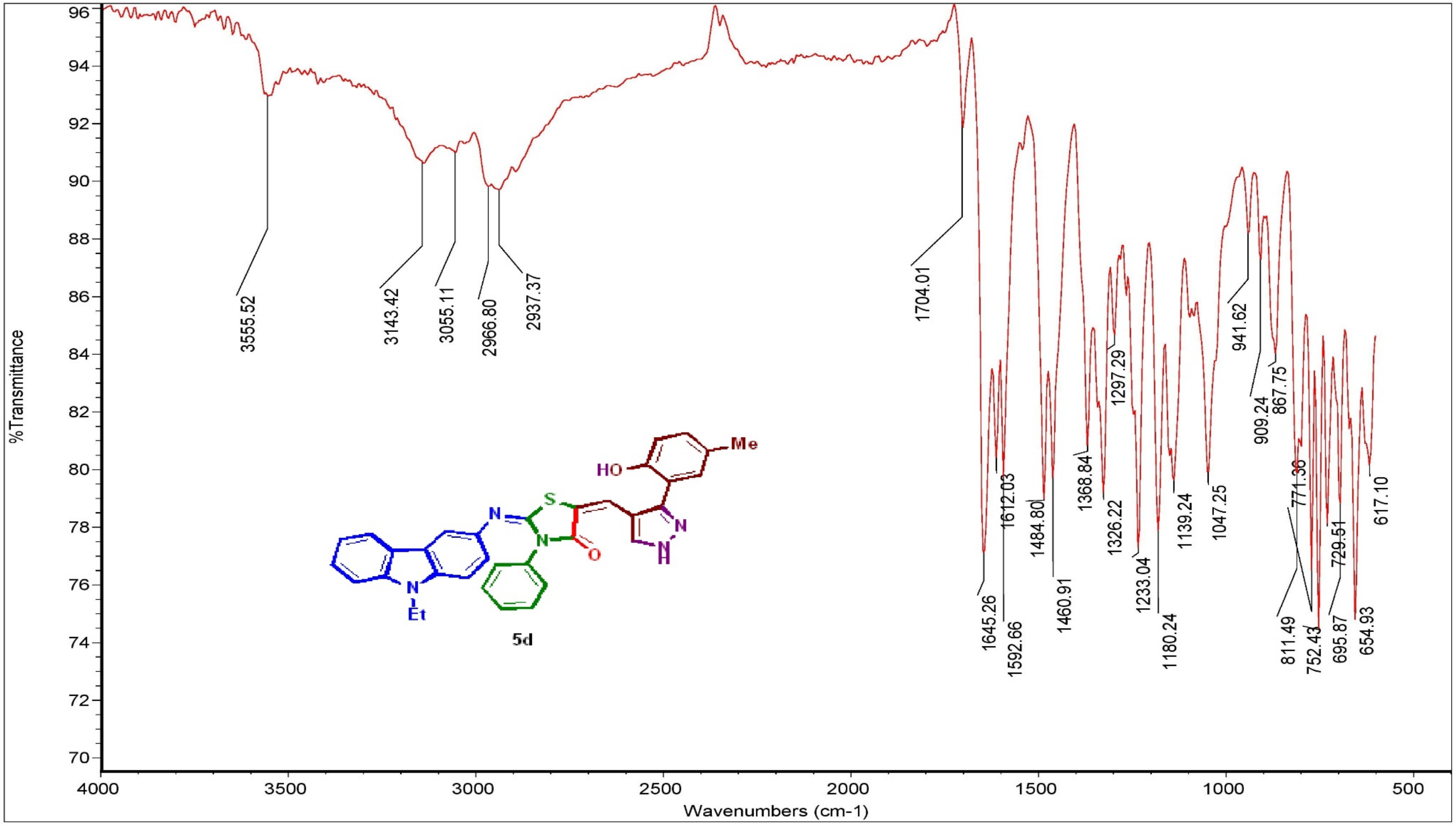

Figure S66: The IR spectrum of compound **5d**.

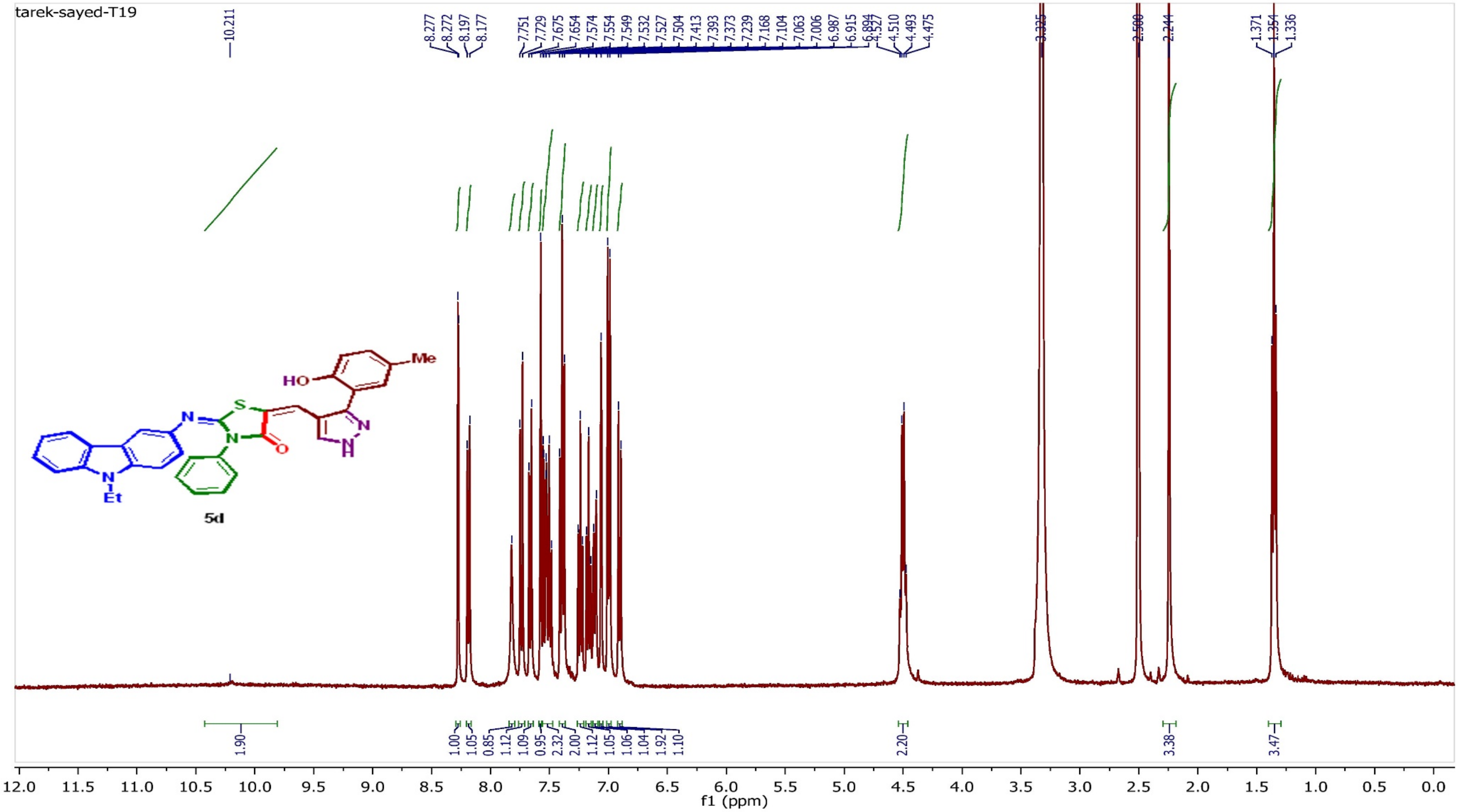

Figure S67: The ^{1}H-NMR spectrum of compound **5d**.

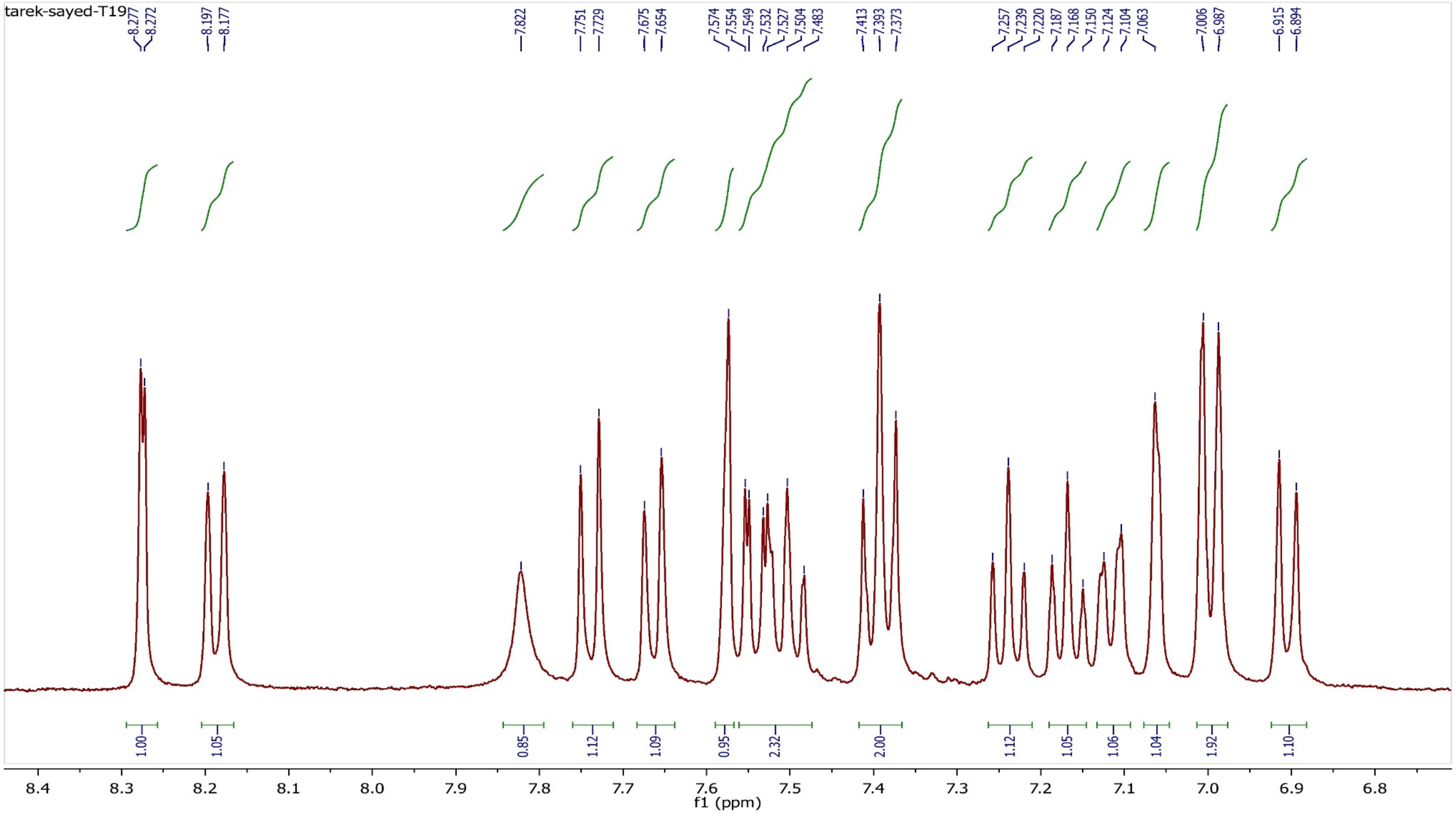

Figure S68: The enlargement of downfield region in ^{1}H-NMR spectrum of compound **5d**.

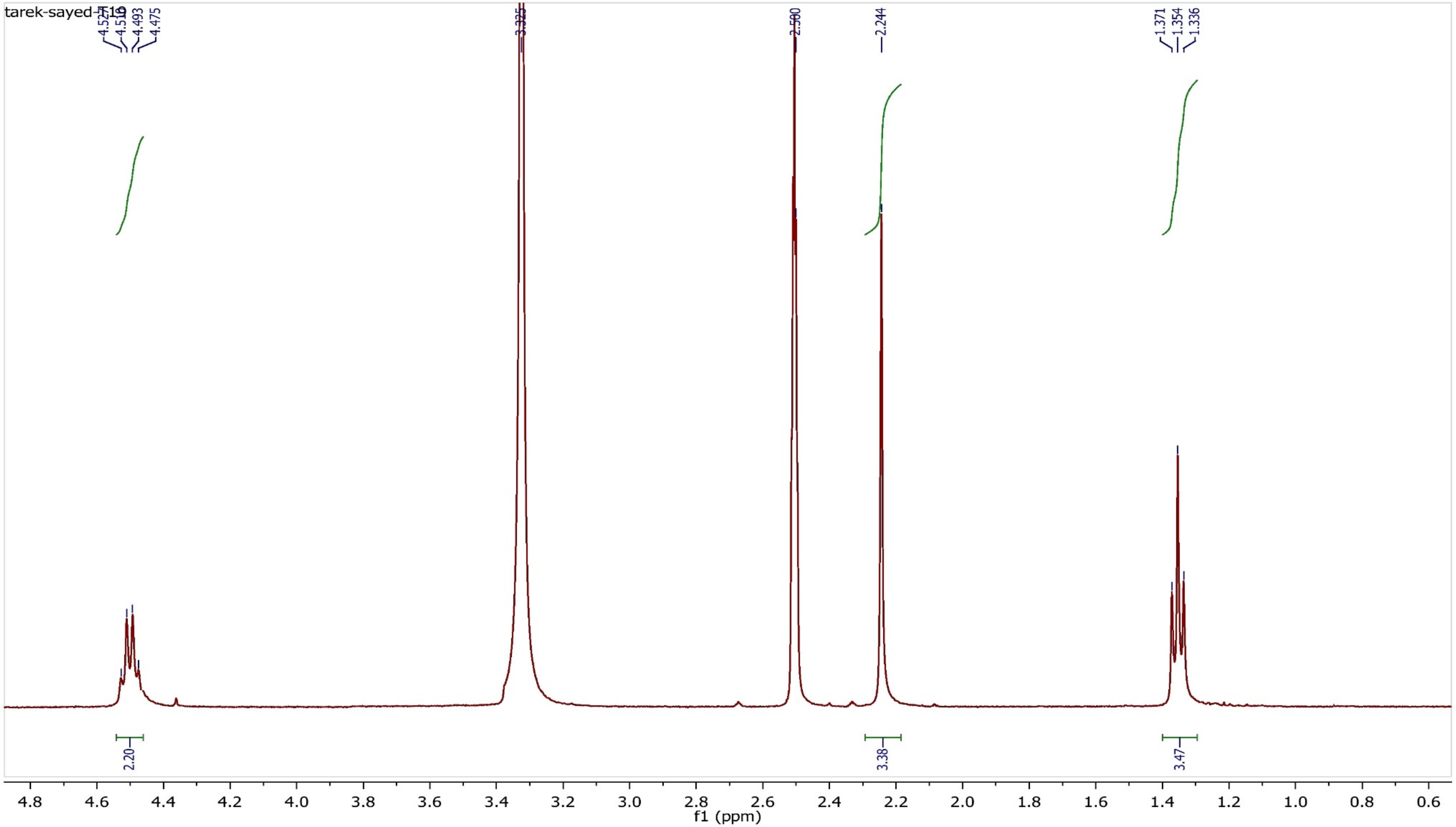

Figure S69: The enlargement of upfield region in ^{1}H-NMR spectrum of compound **5d**.

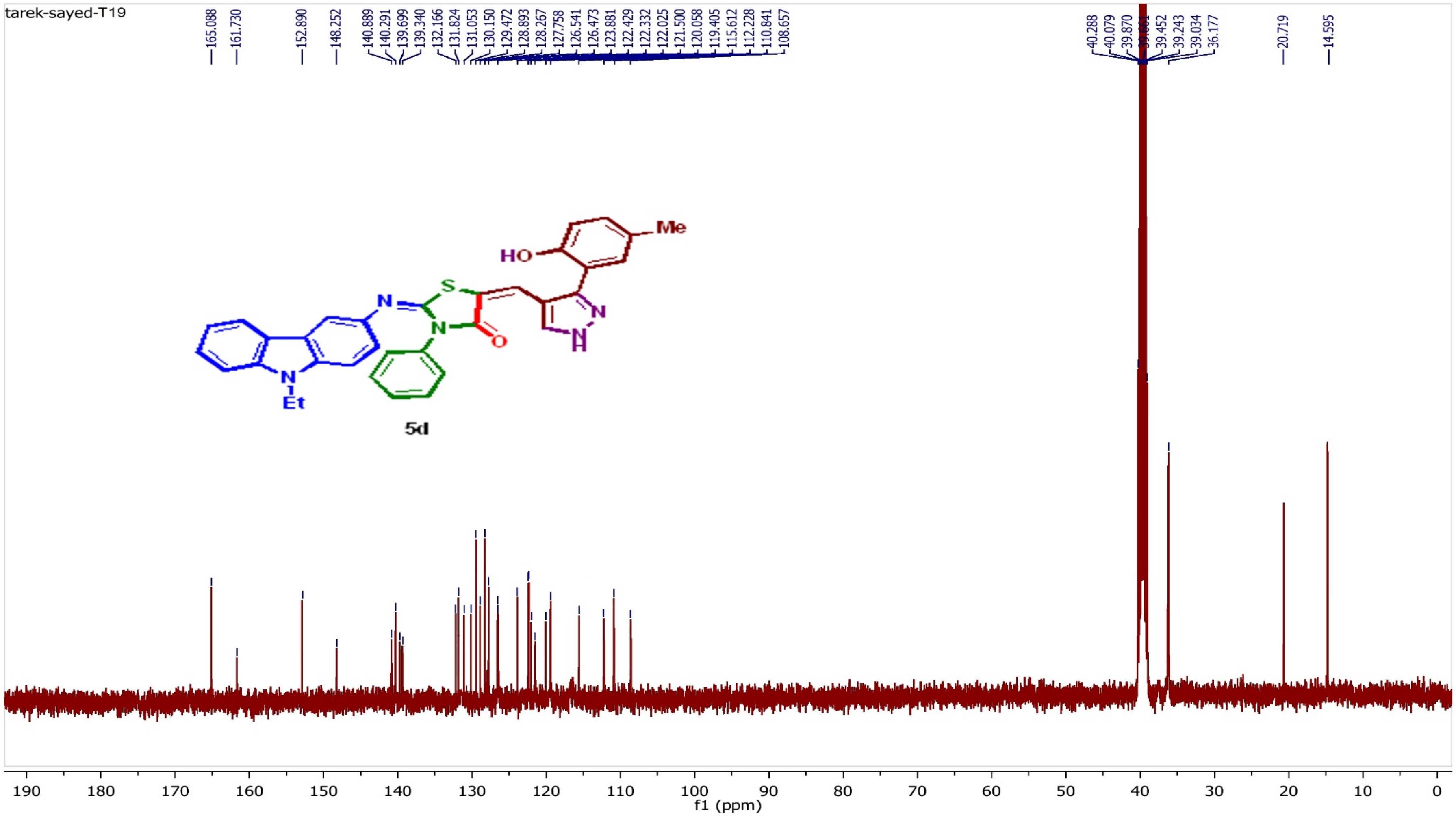

Figure S70: The ^{13}C-NMR spectrum of compound **5d**.

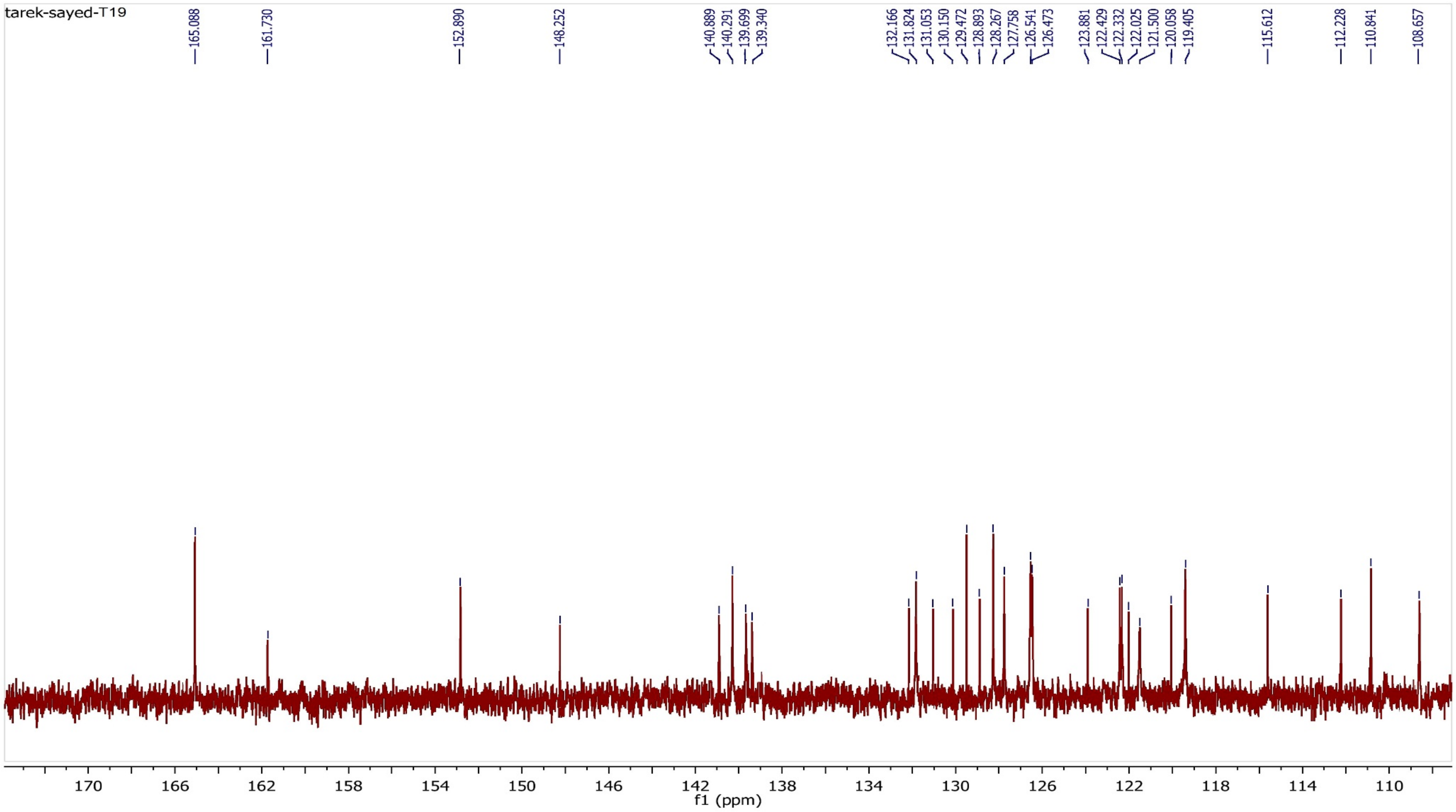

Figure S71: The enlargement of downfield region in ^{13}C-NMR spectrum of compound **5d**.

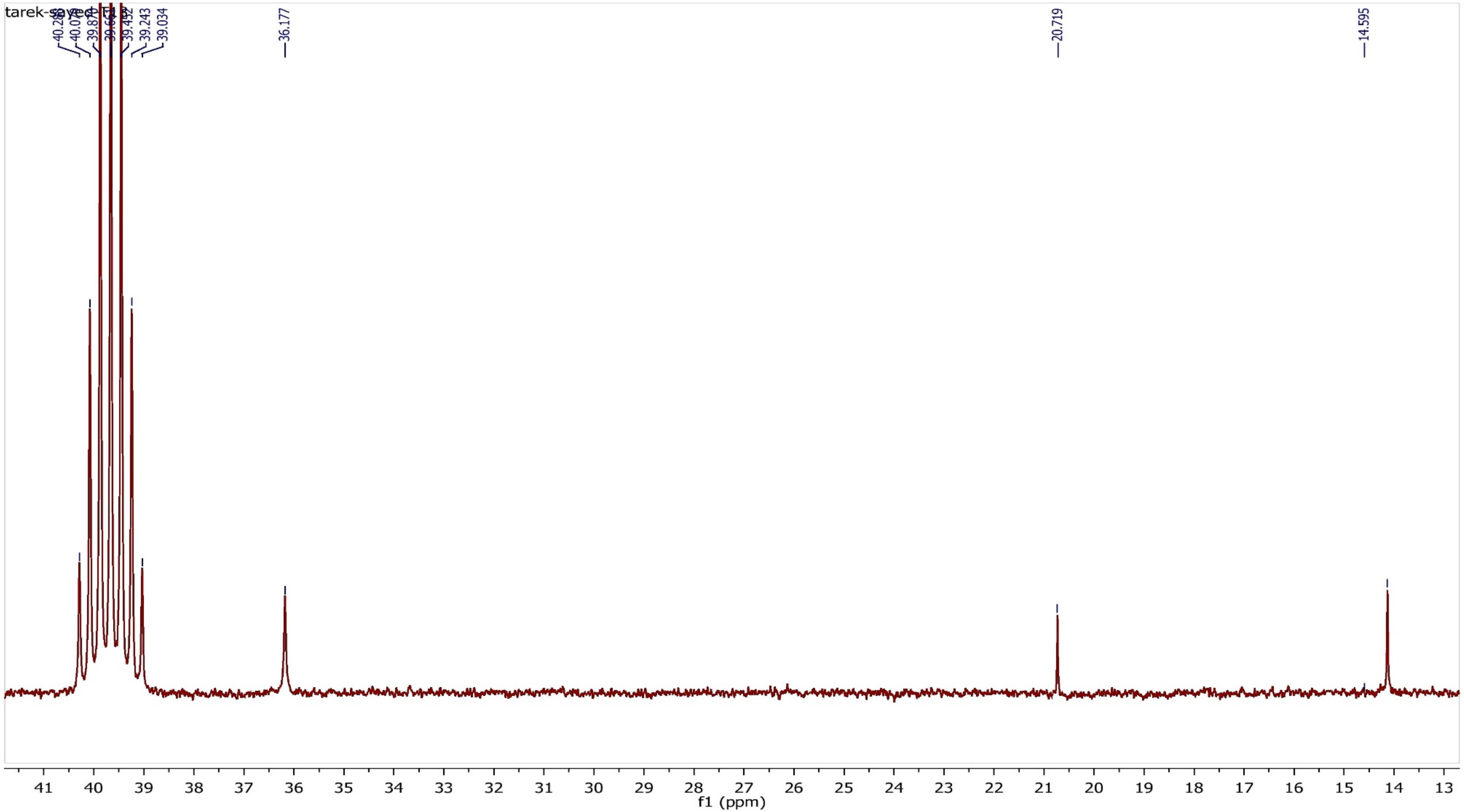

Figure S72: The enlargement of upfield region in ^{13}C-NMR spectrum of compound **5d**.

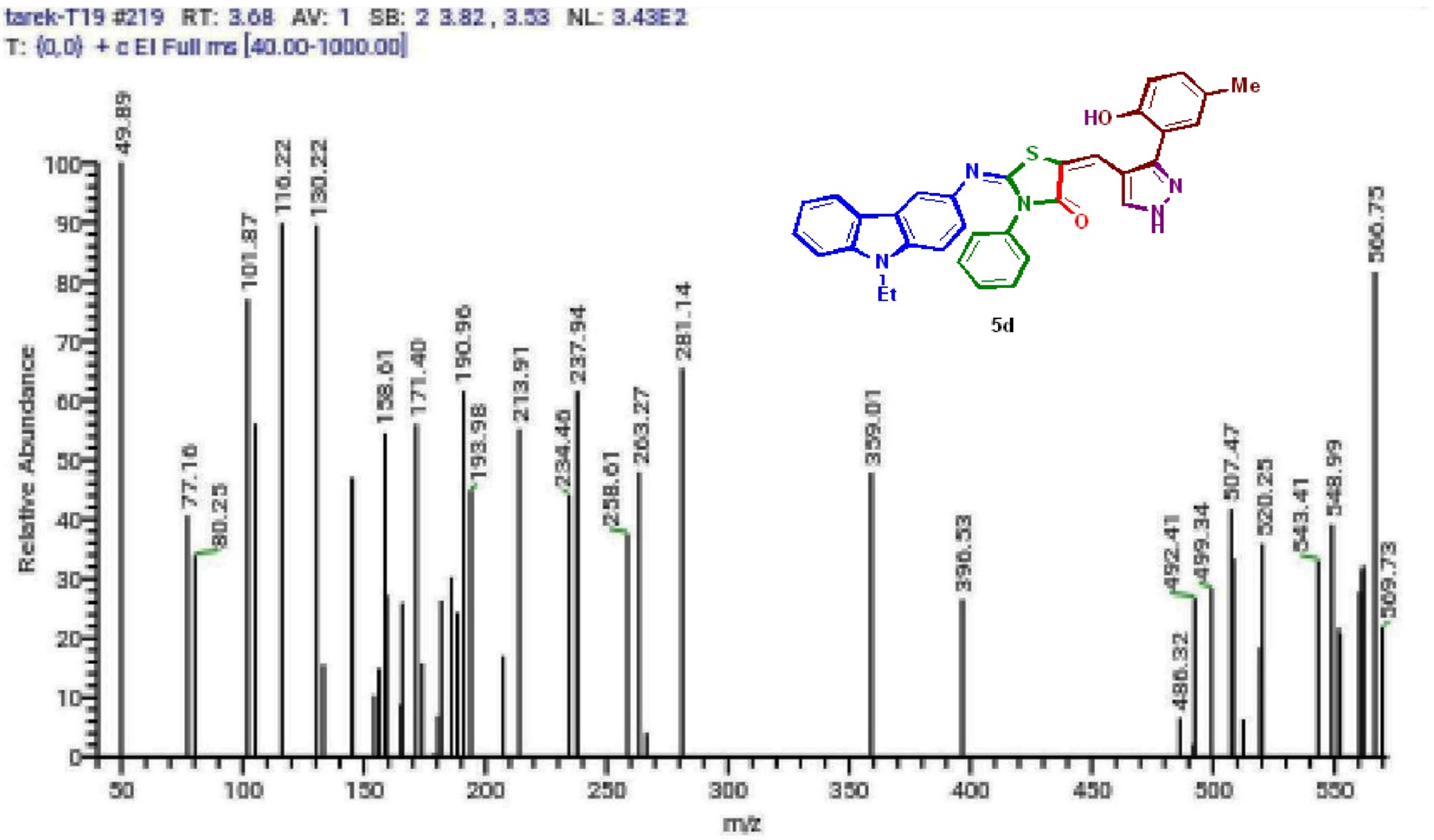

tarek-T19 #219 RT: 3.68 AV: 1 SB: 2 3.82 , 3.53 NL: 3.43E2
T: {0,0} + c EI Full ms [40.00-1000.00]
Relative Abundance
m/z
49.89
77.16
80.25
101.87
116.22
130.22
158.61
171.40
190.96
193.98
213.91
234.46
237.94
258.61
263.27
281.14
359.01
396.53
486.32
492.41
499.34
507.47
520.25
543.41
548.99
566.75
569.73
HO
Me
S
N
O
NH
Et
5d

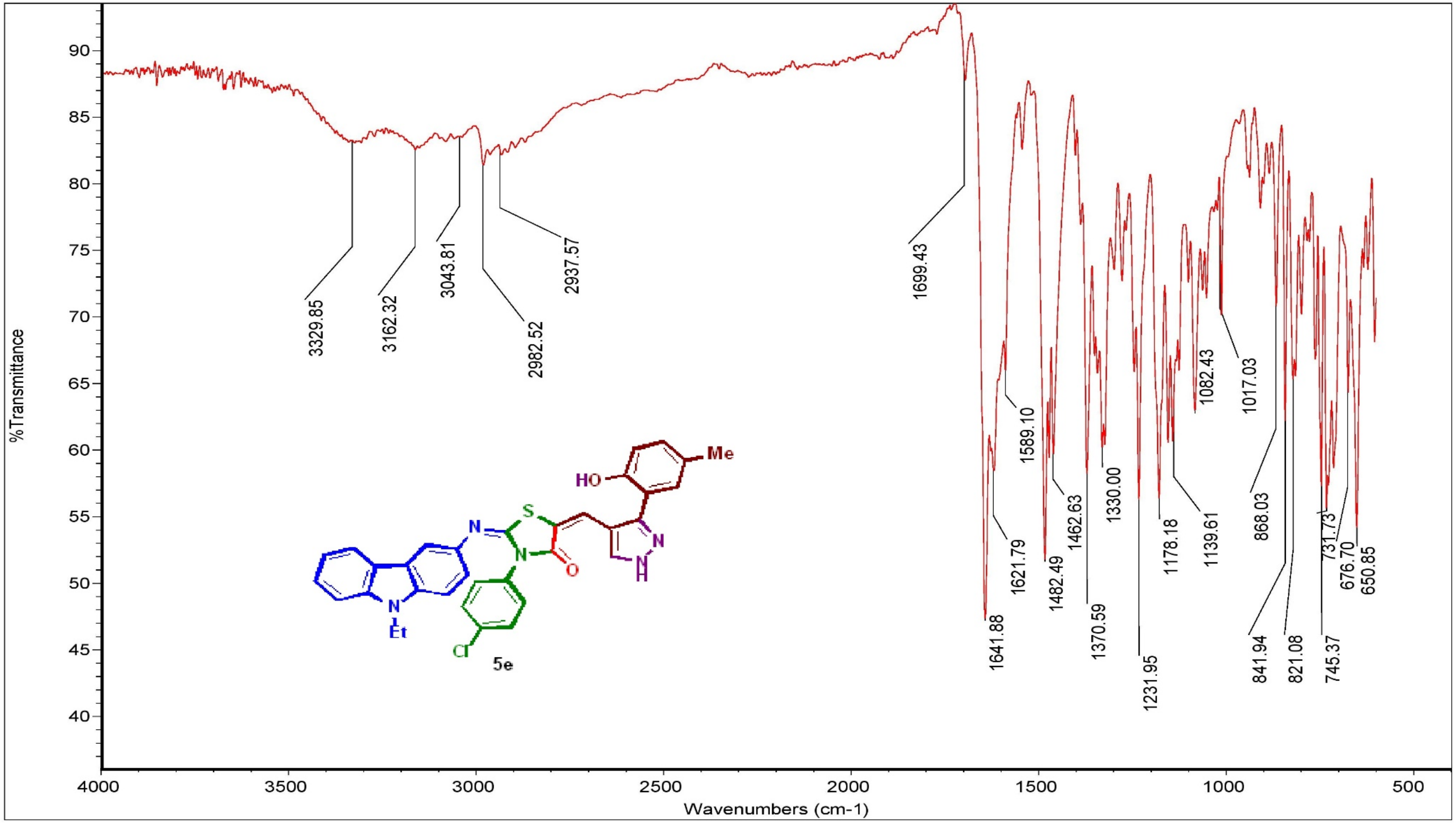

Figure S74: The IR spectrum of compound **5e**.

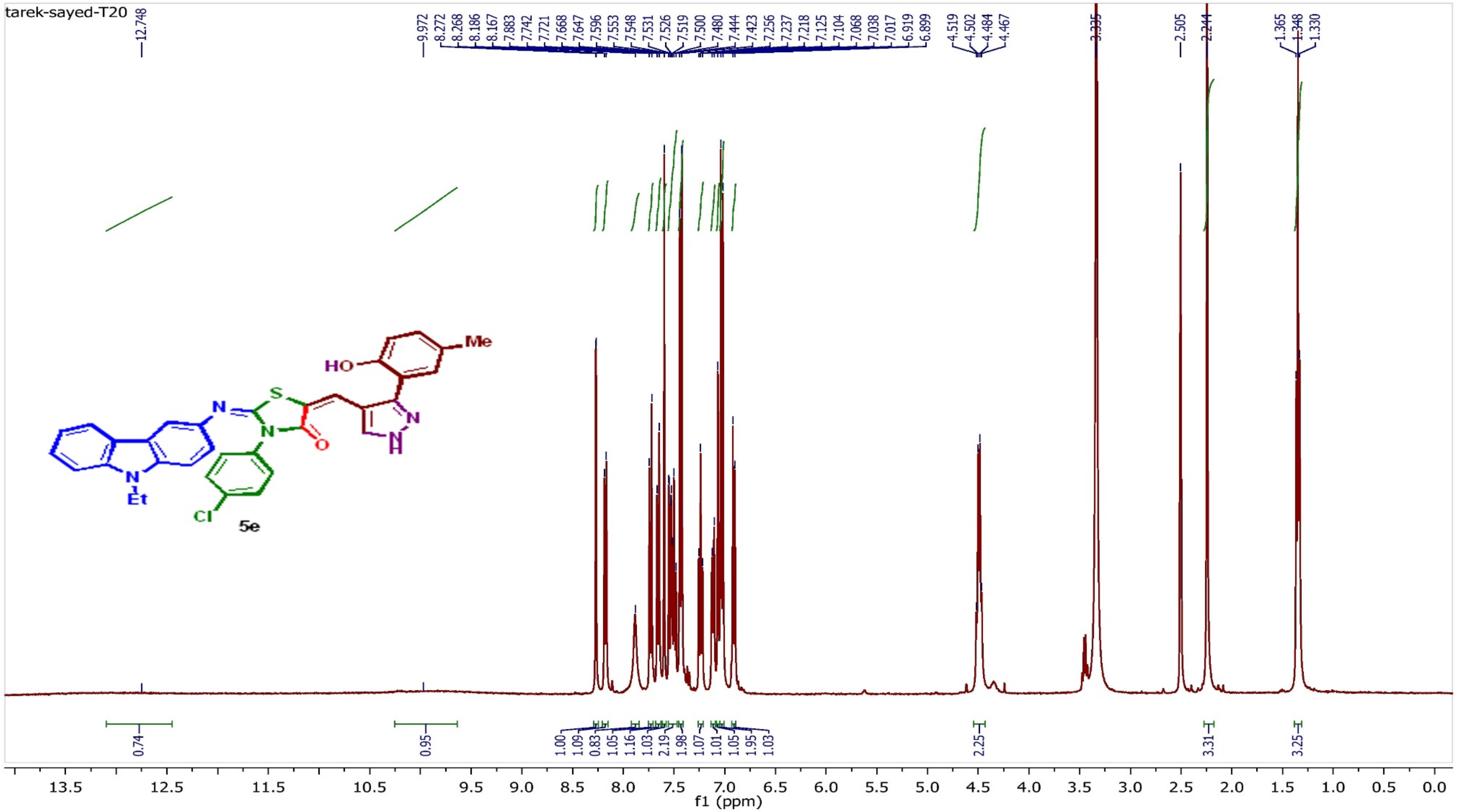

Figure S75: The ^{1}H-NMR spectrum of compound **5e**.

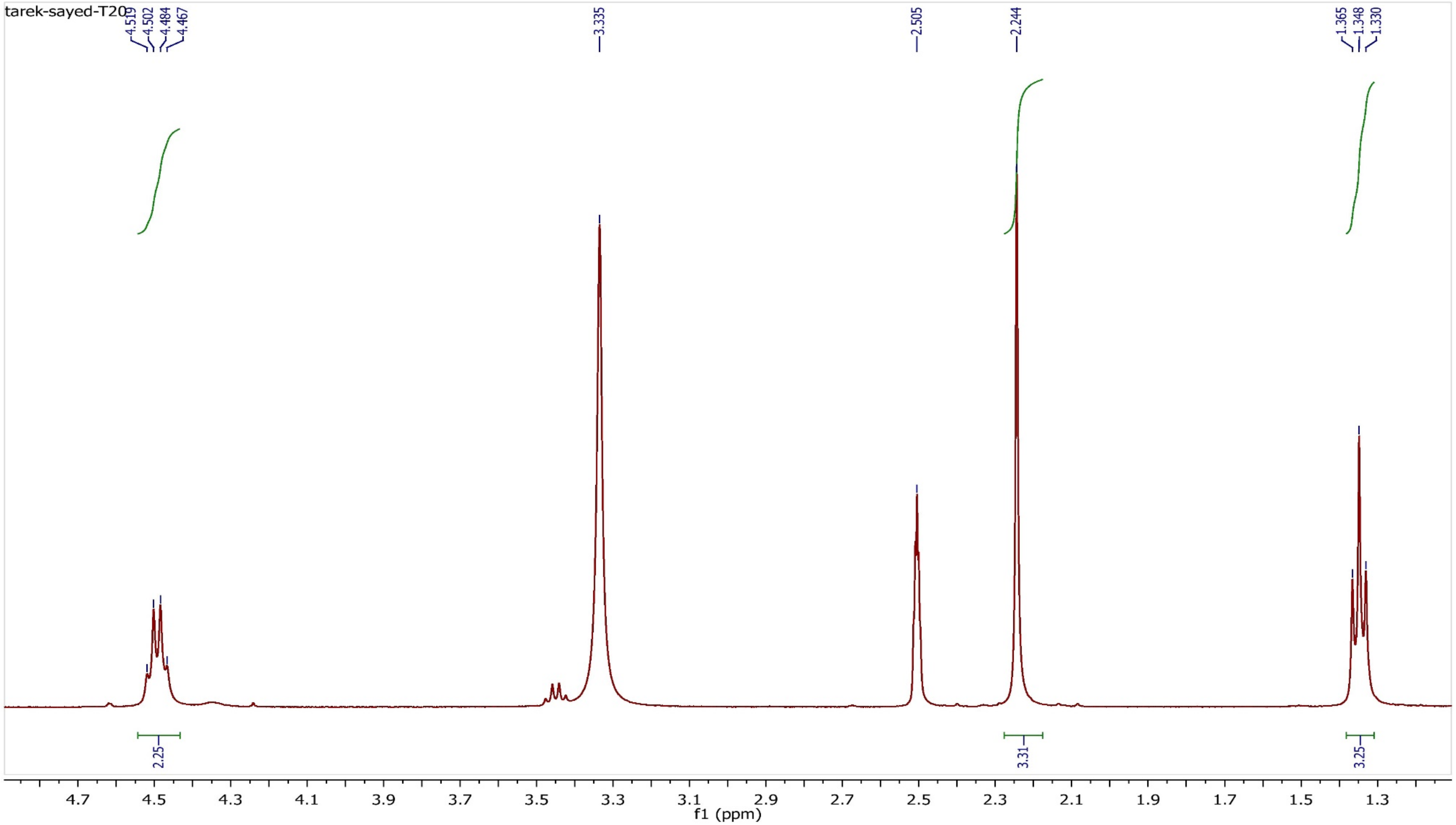

Figure S76: The enlargement of upfield region in ^{1}H-NMR spectrum of compound **5e**.

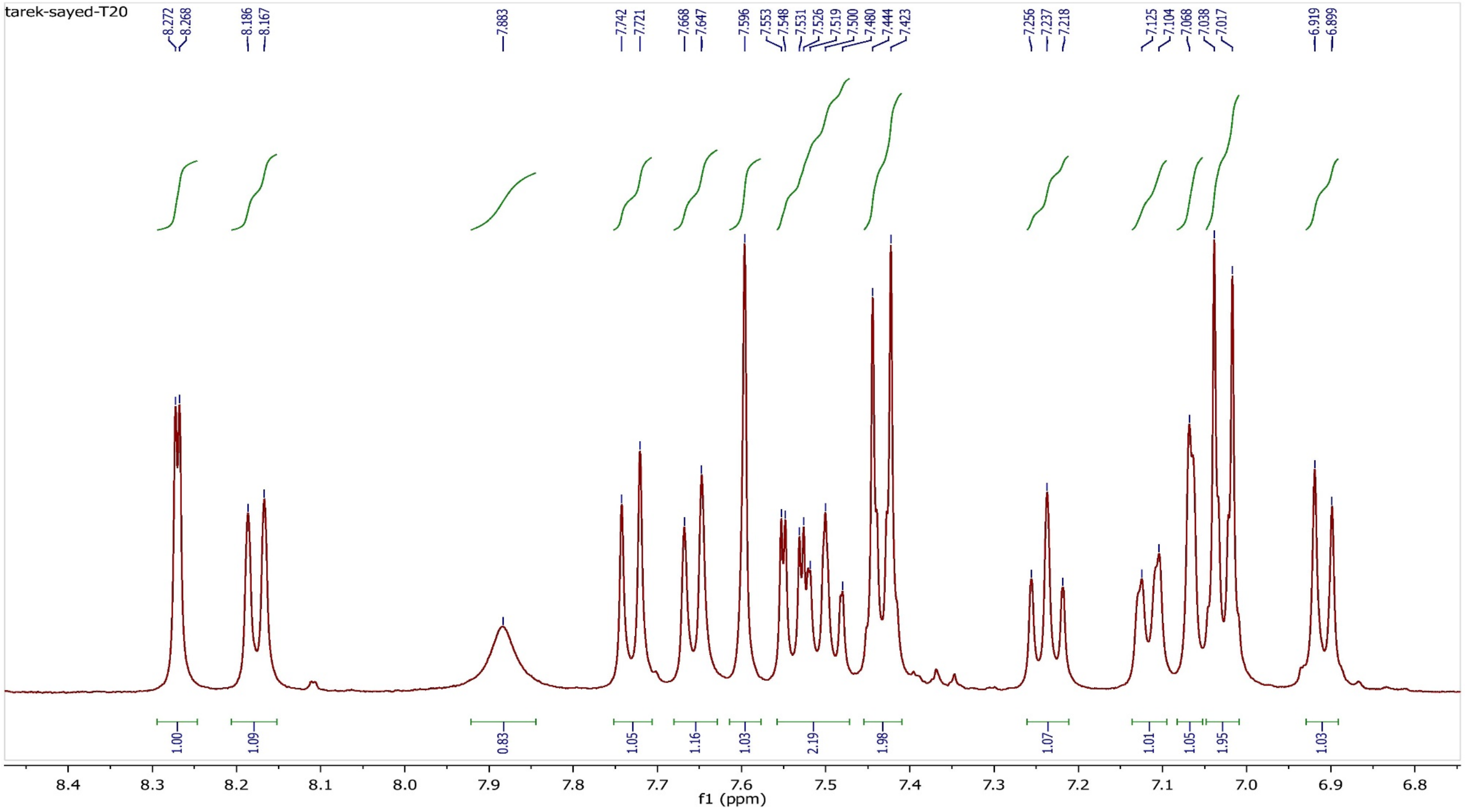

Figure S77: The enlargement of downfield region in ^{1}H-NMR spectrum of compound **5e**.

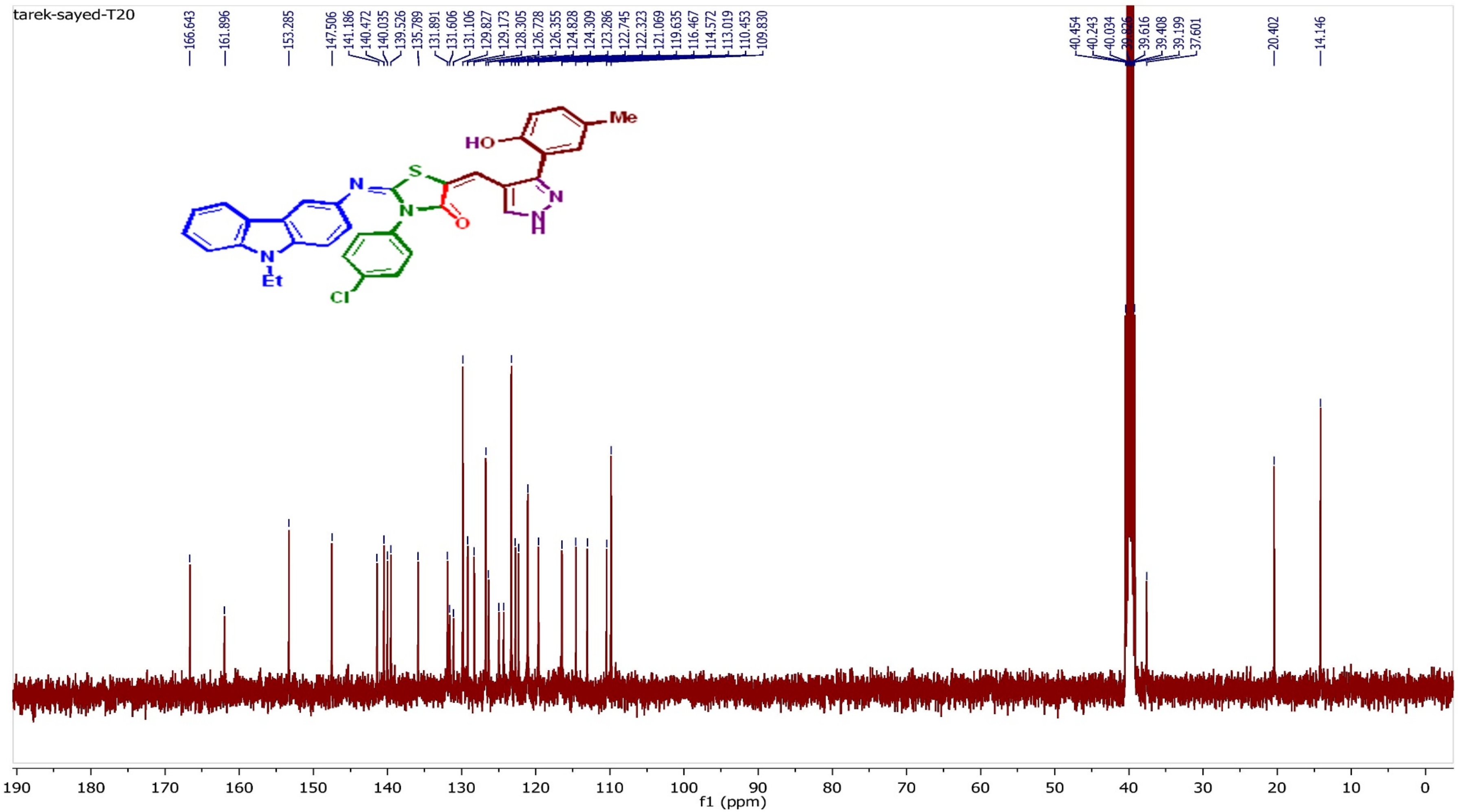

Figure S78: The ^{13}C-NMR spectrum of compound **5e**.

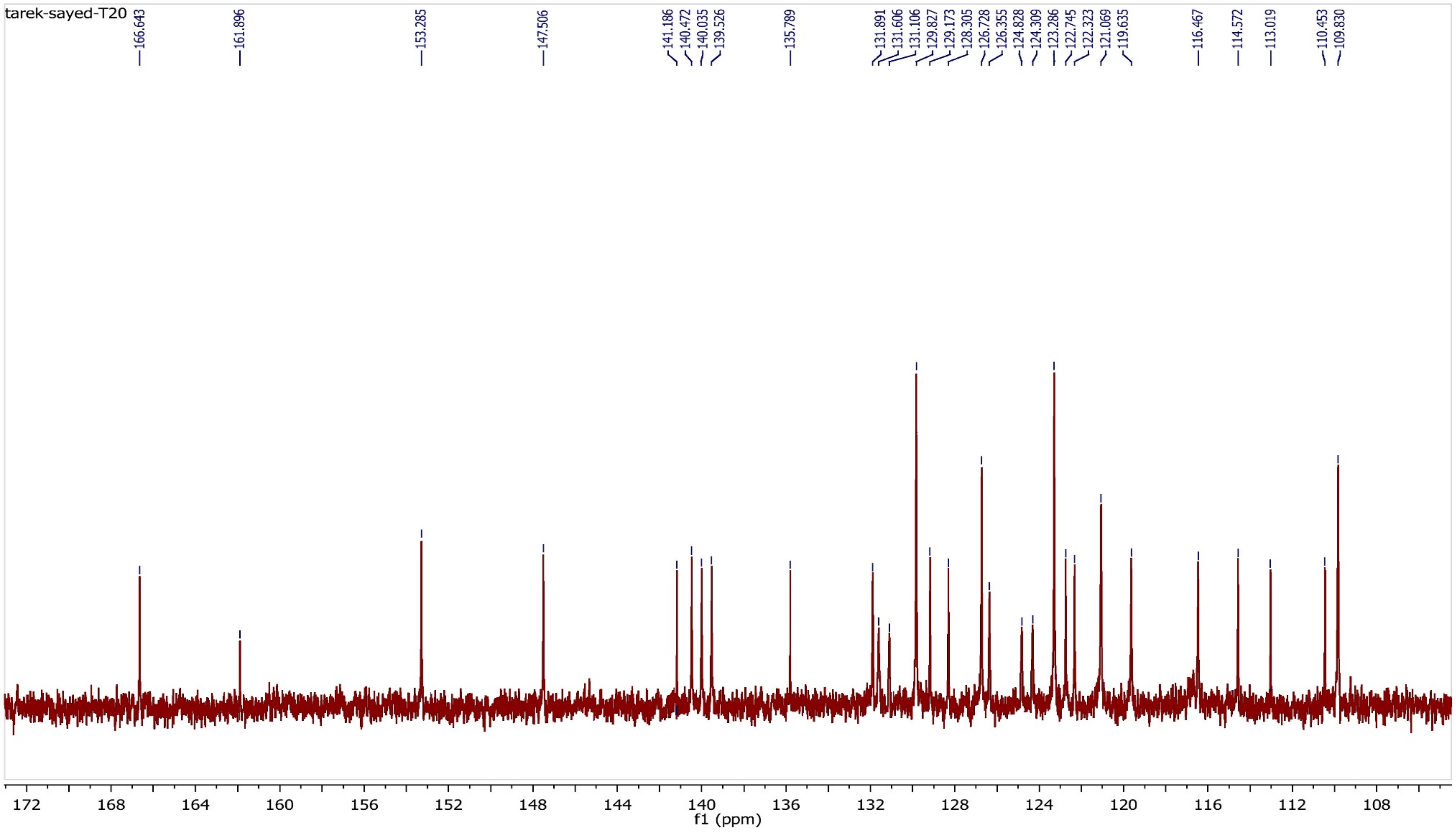

Figure S79: The enlargement of downfield region in ^{13}C-NMR spectrum of compound **5e**.

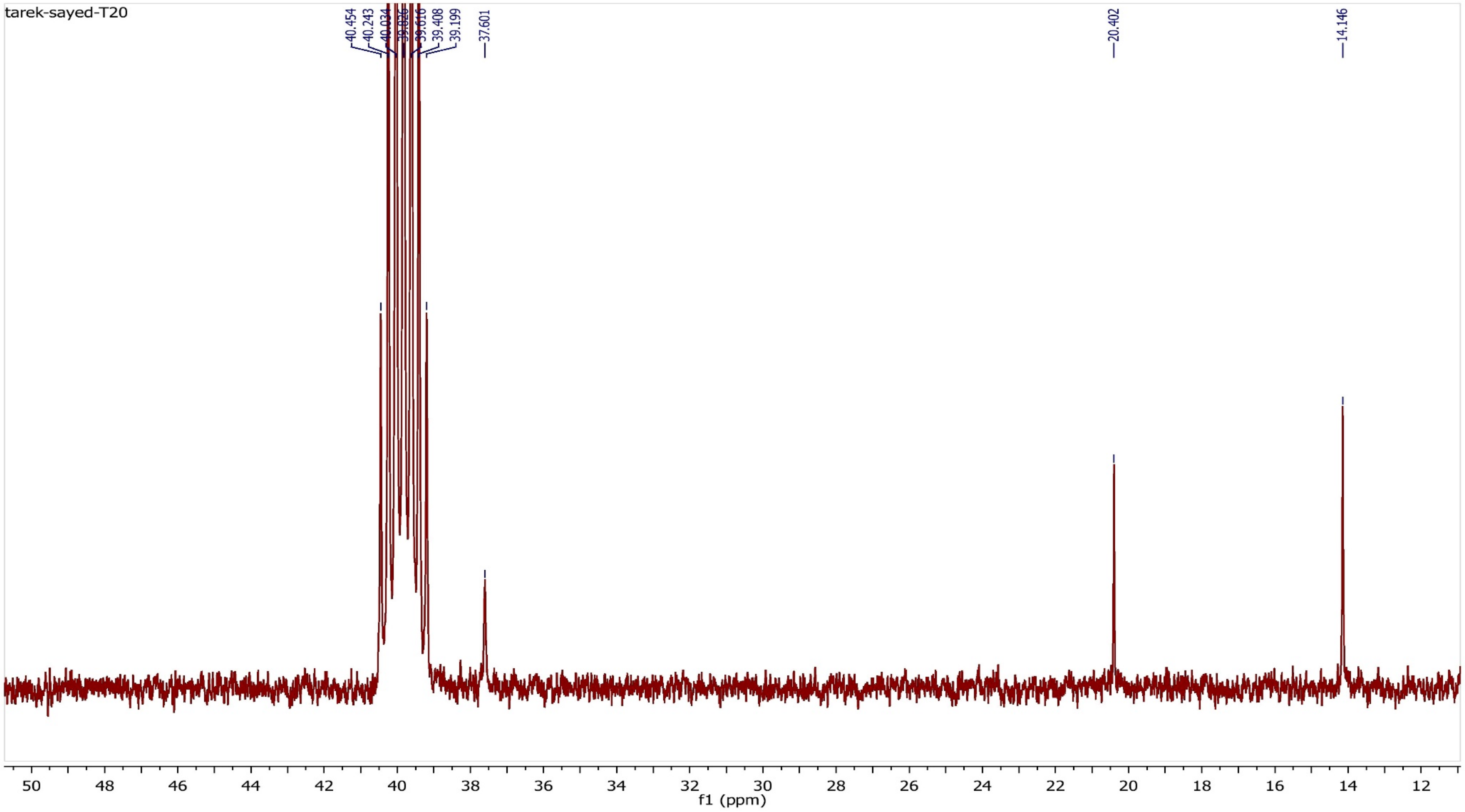

Figure S80: The enlargement of upfield region in ^{13}C-NMR spectrum of compound **5e**.

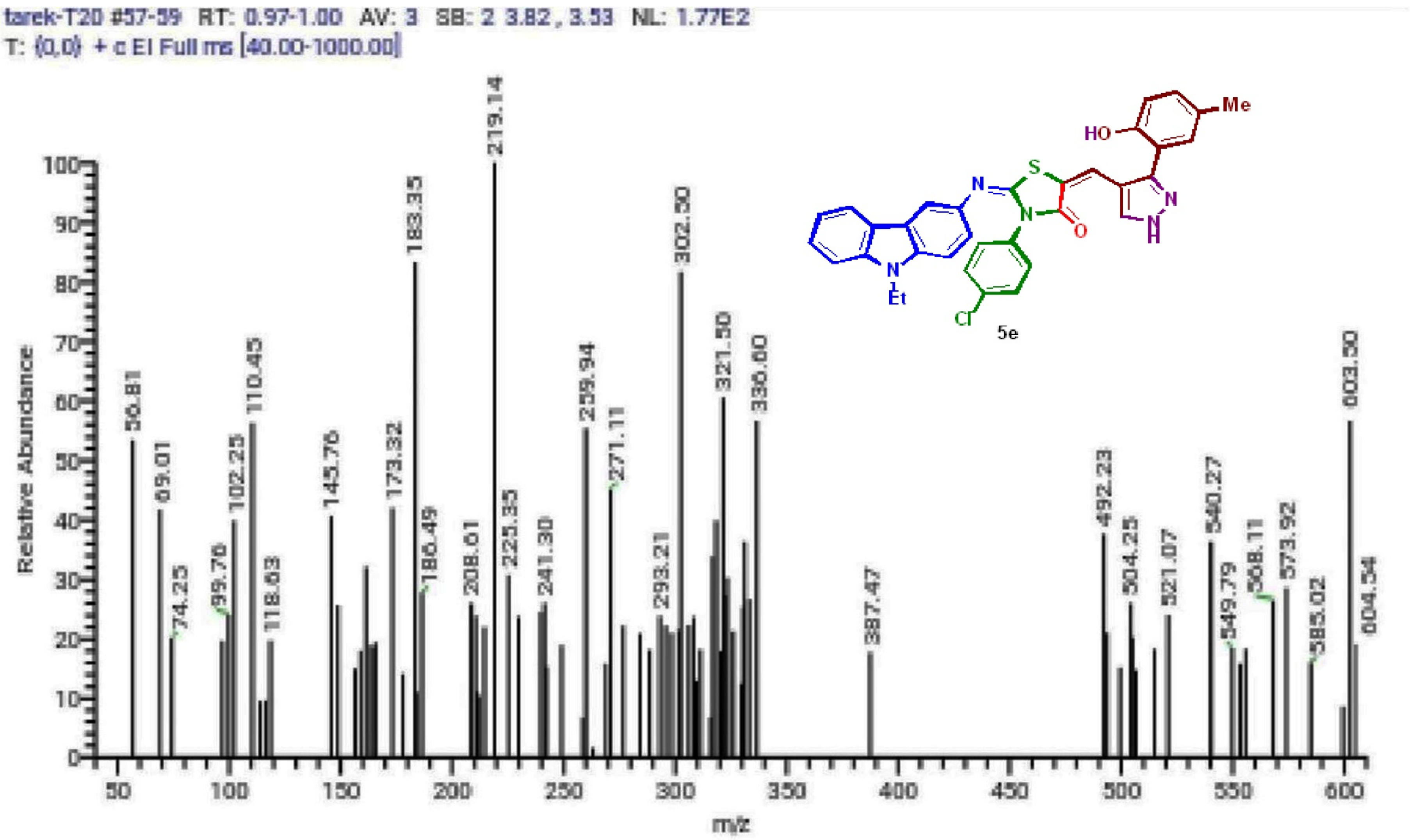

Figure S81: The mass spectrum of compound **5e**.
